# Automated Chemical Profiling of Wine by Solution NMR Spectroscopy: A Demonstration for Outreach and Education

**DOI:** 10.1021/acs.jchemed.5c00652

**Published:** 2026-01-06

**Authors:** Lily Capeci, Ruoqing Jia, Mary E. Peek, Miriam K. Simma, Elizabeth A. Corbin, F. N. U. Vidya, Hongwei Wu, Johannes E. Leisen, Andrew C. McShan

**Affiliations:** School of Chemistry and Biochemistry, Georgia Institute of Technology, Atlanta, Georgia 30332, United States

**Keywords:** general public, first-year undergraduate/general, public understanding/outreach, biochemistry, inquiry-based/discovery learning, biophysical chemistry, agricultural chemistry, NMR spectroscopy

## Abstract

Profiling complex chemical mixtures provides a unique opportunity for outreach and education. Here, we develop a teaching module using one-dimensional ^1^H nuclear magnetic resonance (NMR) spectroscopy to study wine. The demonstration is delivered at the Georgia Tech NMR Center during the Atlanta Science Festival to 127 visitors from diverse backgrounds, including families with K–12 children. Participants interact with wine samples through cognitive and sensory experiences. Visitors engage with a live demonstration of a high-field NMR spectrometer, including real-time measurement and automated analysis of ^1^H NMR spectra for 14 wines. The module enables instructors to showcase how an NMR spectrometer works, quantify 70 wine metabolites, and discuss how chemical composition relates to wine characteristics. Practical examples highlight how ^1^H NMR can detect wine fraud. An exit survey suggests that the module increases public interest and excitement for research. Adults report learning factors affecting wine characteristics and the basics for how NMR is applied to research. K–12 students retain information about wine composition and enjoy sensory aspects of the activity. A description of the demonstration’s design and implementation is provided to facilitate its adoption in outreach efforts and introductory chemistry courses.

## Graphical abstract

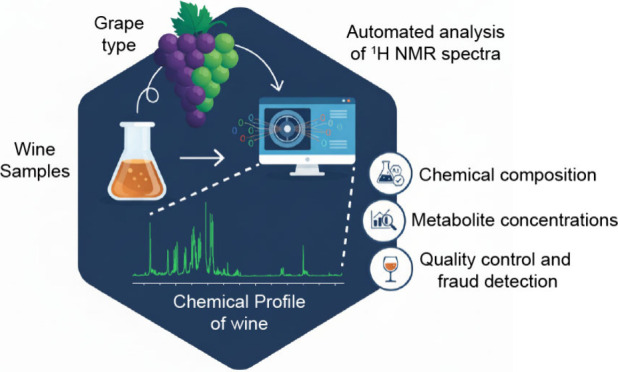

## Introduction

Nuclear magnetic resonance (NMR) spectroscopy is a technique for analyzing the chemical composition of complex mixtures. The most common form is one-dimensional (1D) ^1^H solution NMR. In 1D ^1^H NMR, signals from hydrogen atoms appear at unique positions in the NMR spectrum depending on the type of atom they are bonded to and the surrounding chemical environment.^1^ Peak positions and splitting patterns provide information on molecular identity, while signal intensity quantifies concentration.^2^ Teaching NMR theory is challenging because it requires students to grasp abstract quantum mechanical principles, apply advanced mathematics, integrate knowledge across disciplines, learn an extensive vocabulary, and extract meaning from complex spectral patterns.^3–5^ Several strategies for teaching NMR theory have been developed, including product operator formalism^6,7^ and simplified vector-based depictions of pulse sequences.^8,9^ However, these approaches require a strong foundation in mathematics, physics, and chemistry, and are not strictly necessary to be able to acquire and interpret 1D ^1^H NMR data.

Rather than theory, practical NMR spectroscopy is more commonly taught through lectures or laboratory courses.^10–13^ In these settings, instructors focus on interpreting NMR spectra and linking peak positions, splitting patterns, through-bond correlations, and nuclear Overhauser effects to sample composition and molecular structure.^14–16^ Surveys indicate that practical knowledge is considered by both students and instructors to be more essential for routine NMR than theory.^17^ Practical NMR has been taught using computer modeling,^18,19^ problems solving Web sites,^20–23^ and hands-on analysis of real-world data.^24–28^ A practical solution NMR course-based undergraduate research experience to study protein–protein interactions was developed by the Traaseth lab as a module for a biochemistry laboratory.^29^ The module provides a formalized framework for students to develop hypotheses, perform research, and analyze real-world NMR data to address outstanding problems in human health. The incorporation of alternative teaching methods that emphasize active learning and collaboration, such as guided-inquiry,^30^ team-based gaming,^31^ and cooperative learning,^32^ has made practical NMR accessible to students from diverse backgrounds. Together, these efforts support that real-world examples can be a powerful approach to teach practical NMR to students from all types of backgrounds, even in the absence of a robust theoretical description of the technique.

Beyond a formal classroom setting, opportunities to expose the general public to practical NMR are rare, and most individuals have never encountered the technology. However, NMR can serve as a powerful tool for STEM outreach. In an outreach context, rather than emphasizing theory, interactive modules can engage participants to illustrate how NMR can help solve research problems.^33,34^ One example is the National High Magnetic Field Laboratory’s Open House in Tallahassee, Florida (https://nationalmaglab.org/openhouse). The public is exposed to demonstrations where they interact with scientists to learn about STEM research through the lens of NMR. This type of event has several impacts across the different types of participants: it promotes community engagement with expert scientists; it ignites curiosity and inspires future scientists; it provides an opportunity to learn about the basic principles of magnetism; and it fosters an inclusive culture of scientific inquiry and collaboration.^35–38^ Efforts have also introduced NMR to high school students through low-field NMR instruments, where the ability to observe an instrument in operation and analyze samples enhanced learning through active engagement and real-world applicability.^39,40^ Another example is the introduction of NMR structures of biomolecules through virtual reality headsets that showcase atom connectivity in 3D space.^41^ More progress is needed to develop new NMR-based demonstrations and learning modules for outreach activities, chemical education initiatives, and university coursework.

1D ^1^H NMR is one of the most widely used analytical techniques for characterizing the chemical composition of complex mixtures, including biological, environmental, and industrial samples.^42,43^ Recent advances in NMR methodologies, including novel pulse sequences, instrument hardware, and automated analysis software, have enabled high-throughput workflows to define sample composition and quantify concentrations.^44,45^ Review articles highlight the practical application of NMR to food science where researchers uncover insights not only into chemical composition, but into the molecules responsible for taste, aroma, nutritional value, shelf life, and health.^46–48^ Wine profiling provides an excellent opportunity for outreach and chemical education at the intersection of public and scientific interest. Wine is an alcoholic beverage made from fermented grape juice.^49^ The production and sale of wine represent a multibillion-dollar industry.^50,51^ More than 60% of the global population over 18 years of age consumes wine.^51,52^ Different wines exhibit distinct characteristics whose origins are complex, but generally defined the region of production (regionality, climate, soil types, terrain), the fermentation processes, and grape variety.^53–55^ The rich history, chemical diversity, and real-world applicability make wine an ideal tool for outreach and chemical education efforts.^56–61^ Approaches for chemical profiling of wine are well established where analytical chemistry techniques, such as Raman spectroscopy, mass spectrometry, and solution NMR, play a pivotal role in quantifying the more than 70 different components.^62–65^

Wine profiling can be enhanced through automation and machine learning of spectral data.^66–69^ There are several software for automated wine chemical profiling, such as Bruker’s Wine-Profiling 4.0 (requires purchase of a license) and the Wishart lab’s MagMet-W (freely available Web server).^68,70^ To our knowledge, these tools have not yet been applied to outreach or chemical education efforts. One of the major bottlenecks for introducing NMR to nonexperts is the technical nature of data acquisition, processing, and analysis.^71^ We believe that the incorporation of automated workflows via accessible platforms, such as MagMet-W, will enable instructors to present NMR within a framework that is both equitable and accessible to participants of outreach efforts and formal university coursework. We did not identify any published works centered on the automated analysis of 1D ^1^H NMR data to study wine, despite its extensive use in both academic and industrial settings.^72^

## Materials and Procedures

The materials and a complete instructional tutorial to conduct the experiments is available (Tutorial and Materials and Procedures). An instructional YouTube video is provided (Data Availability section). The Georgia Tech Institutional Review Board considered the anonymous surveys exempt from approval requirements as they were normal educational practices in common, established educational settings. We did not collect any identifying information. All participants were informed of the purpose of the survey.

## Hazards and Safety Precautions

Participants should be aware of associated hazard of NMR magnets, including magnetic field hazards, medical implants, wiping of credit cards/cell phones, chemical safety, and cryogens.^73^ Participants should also be aware that while high-field spectrometers are typically harmless, exposure to magnetic objects, especially medical implants like pacemakers, should be avoided. Participants will likely not be directly exposed to cryogens (liquid nitrogen and liquid helium). However, care must be taken if they visit NMR laboratories. Any chemicals should be handled in a separate space away from NMR spectrometer workstations.

Students and instructors should wear appropriate personal protective equipment, including protective clothing, masks, gloves, and goggles. Depending on the implementation of the demonstration, chemical hazards involved could be D_2_O, methanol, lead­(II) acetate, and diethylene glycol. Methanol is highly volatile and flammable. It should be handled under a chemical fume hood with proper supervision. Methanol, lead­(II) acetate, and diethylene glycol compounds pose health hazards (toxicity) when ingested, inhaled, or contacted with skin.^74–76^ The compound 2-chloropyrimidine-5-carboxylic acid (CPCA), used as a phasing standard, is an eye and skin irritant. Wine opened in a chemical laboratory should not be consumed. As per university policy, instructors should make it clear that student consumption of wine is not allowed.

## Results

### Module Design

The design of a module applying 1D ^1^H solution NMR spectroscopy to study wine is outlined in Figure 1 and described below. 1D ^1^H NMR as an ideal approach for wine profiling due to its nondestructive nature, sensitivity, and spectral resolution.^47,55,66,77–80^ The Wishart lab’s MagMet-W Web server served as a foundation due to its ease of use, accessibility (no cost), quantification accuracy, and quick run time (∼7 min per wine).^68,70^ MagMet-W enables nonexperts to process and analyze NMR data in real-time for outreach demonstrations or on-the-fly analysis in university coursework. To inform the design approach, we surveyed the STEM outreach literature. Reported efforts include Duke Chemistry Outreach, Native Schools in Europe, and University of Texas Health San Antonio’s Science Nights.^36–38^ Several consistent themes emerged: using visuals and sensory experiences to make abstract ideas concrete; tailoring content to audience background with simplified, jargon-free explanations; relating the material to everyday life; and incorporating feedback for iterative improvement. Instructors often assessed effectiveness through surveys or informal conversations with participants. Surveys of STEM outreach efforts indicate that common goals of chemistry outreach are learning core science concepts and having fun.^81^ These reports informed our selection of features to spark excitement across age groups, from K–12 students to undergraduates to adults. Several demonstration elements were designed as “wow factors”. Wow factors are teaching elements designed to elicit a strong reaction from participants through visual, cognitive, or sensory experiences and are associated with increased engagement, excitement, and content retention.^82^ Examples wow factors are described in the Module Implementation section.

**Figure 1:**
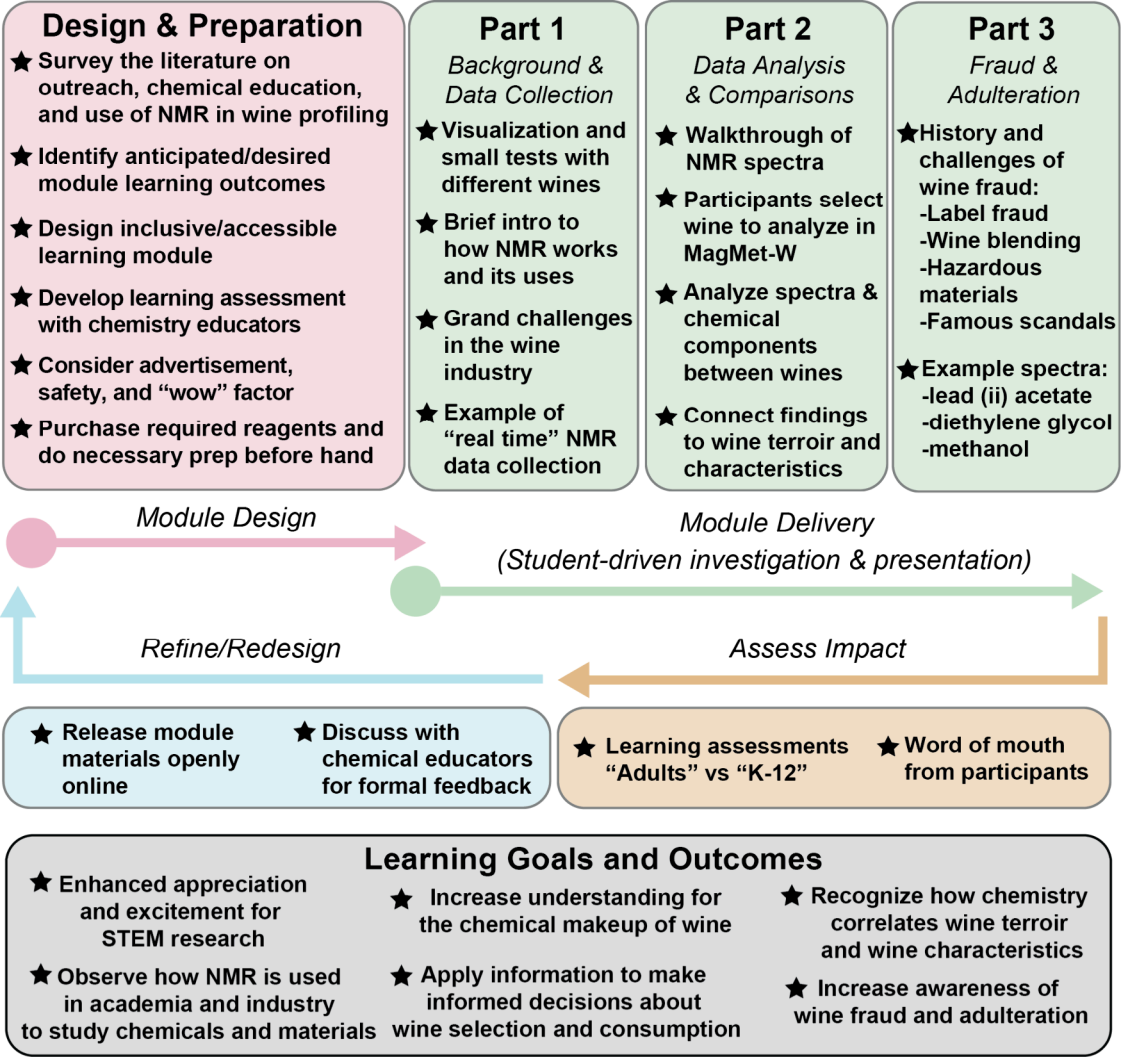
Design and implementation of a learning module for outreach and chemical education centered on the application of 1D ^1^H NMR and MagMet-W to automatically profile wine. A cartoon schematic of the workflow for design and implementation of the demonstration on the use of one-dimensional ^1^H solution NMR to study wine. The demonstration provides students who lead the activity the opportunity to present complex chemical topics to a lay audience. Moreover, the demonstration exposes the public (both K–12 and adult participants) to the background and history of wine profiling and cutting-edge NMR spectroscopy instruments. The schematic also summarizes the intended learning goals and outcomes of the demonstration as well as efforts to iteratively assess impact and refine the demonstration. A similar workflow could be used to implement the activity as a learning module in a formal classroom or laboratory setting. In our implementation, the demonstration focuses on highlighting the application of NMR as a tool to study wine rather than a focus on teaching NMR theory.

### Module Implementation

As a model opportunity to deliver the module, members of the public were engaged as part of the 2025 Atlanta Science Festival, an annual two-week event that promotes inclusive science communication.^83,84^ The event was advertised online and through promotional flyers distributed across campus and the Atlanta metro area (Figure S1). A total of 127 participants attended, typically visiting in groups of 6 to 20. Attendees represented a broad age range (5–75 years old) and educational backgrounds from elementary school to Ph.D. degrees. A group of Scouts of America students participated and fulfilled requirements for a Chemistry Merit Badge. Most attendees had limited knowledge of chemistry or physics, though all were familiar with wine. For many, this was their first exposure to NMR spectroscopy and observing a NMR instrument. Three attendees reported prior experience with NMR from undergraduate coursework. Materials were purchased and wine samples were prepared beforehand. Fourteen wines representing diverse chemical space, grape cultivar, terroir, and flavor/aroma were chosen in consultations with local wine experts. Participants were given the opportunity to interact by visualization, smelling of different wines, chatting with scientists, choosing which wines to analyze, and identifying wine fraud. An interactive module was designed since similar approaches in lab courses enhance content understanding and retention.^29,85^ Apart from sample preparation and data acquisition time, the demonstration takes approximately 30–45 min to complete per group of participants.

As participants entered the NMR center, they visualized and performed a smell test on the 14 different wines (Figure S2). Smell tests in chemistry laboratories allow students to consider how molecular composition links to sensory properties.^86^ In the context of wine, smell tests allow participants to connect chemical constituents to specific aromas;^87^ for example, esters like ethyl acetate contribute fruity smells, while higher alcohols like isoamyl alcohol contribute banana- or whiskey-like notes. During the smell test, a staff scientist provided a brief history of wine, ties to human health, and grand challenges in the wine industry.^51,88,89^ We highlighted two major challenges. First, wine is a chemically complex mixture with unique flavor and aroma profiles that vary widely among varieties. These properties are determined by chemical composition. Second, wine fraud is a significant issue in both historical and modern contexts where the addition of harmful chemicals causes health problems. Identifying wine properties and wine quality is a challenging but important task for scientists. We noted how NMR is an ideal technique for analyzing wine due to its high sensitivity and resolution, which allow researchers to compare molecular fingerprints across different samples.

The demonstration was held in the Georgia Tech NMR center, which houses eight NMR spectrometers (400 MHz/9.4 T to 800 MHz/18.8 T). At a 700 MHz NMR spectrometer, participants interacted with Ph.D. students who introduced the physical principles of NMR together with its uses in academia and industry.^10–12,20,29^ Teaching NMR to nonexperts is feasible without detailed theory by focusing on what NMR peaks correspond to and describing how researchers use the peaks to interpret complex mixtures. First, we prepared a jargon-free presentation introducing the NMR technique is and outlining how NMR is used by scientists to study complex chemical mixtures like wine (Figure 2A and Simplified NMR Theory). Second, we utilized a cutaway of a decommissioned superconducting NMR spectrometer for teaching purposes (Figure 2B). Apart from serving as a wow factor, the NMR spectrometer cutaway highlights the instrument’s engineering, including the superconducting coils and the cryogen chambers, allowing students to connect abstract theoretical concepts to the actual hardware. Third, the strength of large NMR magnetic fields, which are many thousands of times stronger than the Earth’s magnetic field, was demonstrated by tying a large ferromagnetic paper clip to a string and allowing it to be pulled toward an older, unshielded spectrometer (Figure 2C). This visualized the “invisible” magnetic field, helping participants connect the abstract concept of magnetism to a physical effect.^90^

**Figure 2:**
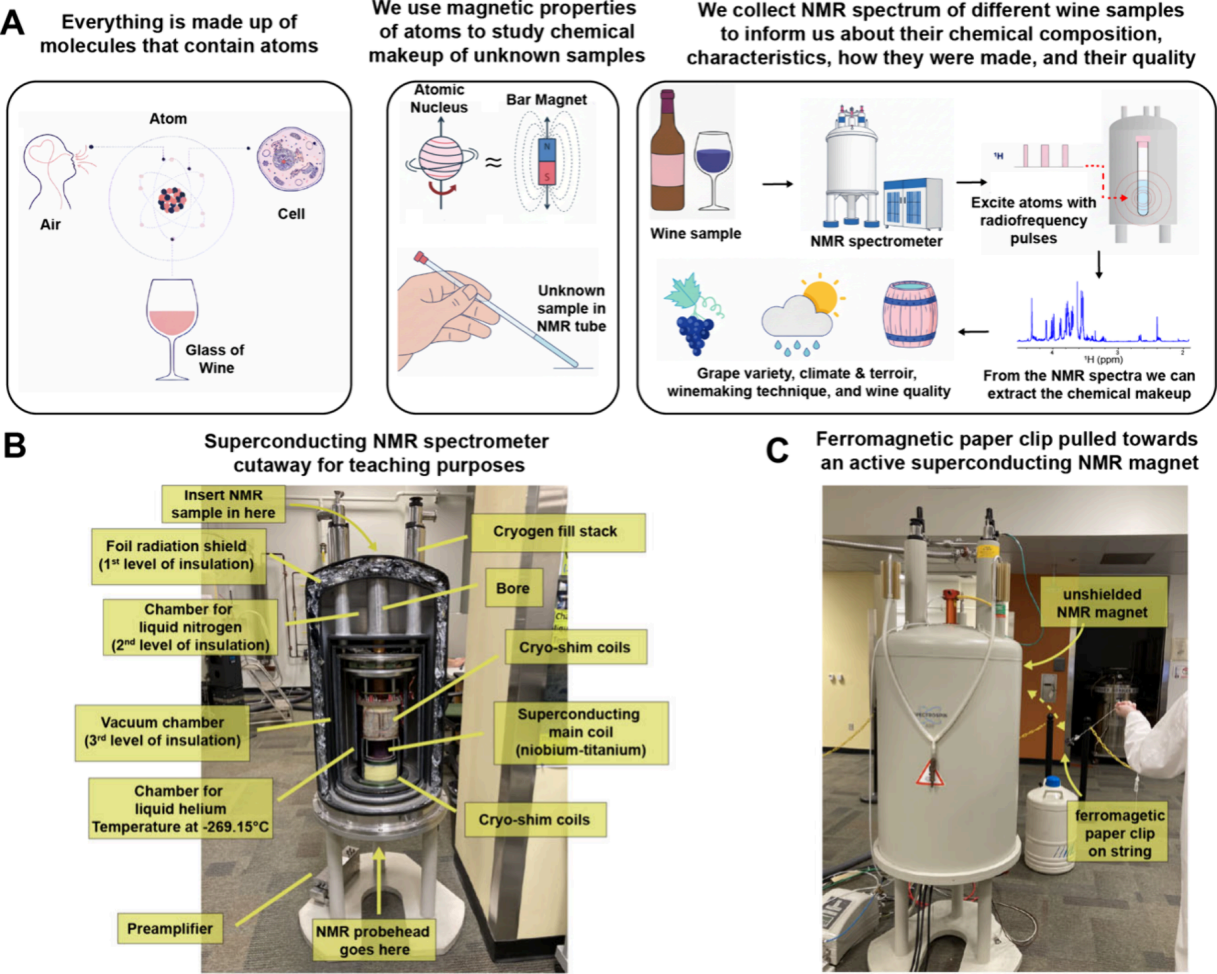
Approach to teaching simplified NMR theory to a public audience without background knowledge of chemistry and physics. (A) Cartoon schematic of the core concepts and workflow for teaching basic NMR theory to lay audiences. An example of a full script is provided in the Simplified NMR Theory file. (B) Annotated photograph of a decommissioned NMR spectrometer cutaway available in the Georgia Tech NMR center. It is used as a tool for teaching both students and visitors. (C) Demonstration of the magnetic strength of an active superconducting NMR spectrometer. A ferromagnetic paper clip attached to a string is pulled toward an older, unshielded NMR spectrometer.

### Data Acquisition and Analysis

A live demonstration of how NMR data is acquired and analyzed was performed with preloaded tubes containing wine samples. Full data acquisition was performed prior to the demonstration, allowing instructors to focus on recording NMR data for a single wine sample. This could be tweaked such that participants record wine samples themselves under instructor supervision. The automated loading of NMR tubes was highlighted with Bruker’s SampleMail and SampleCase where sample transport is controlled by a pneumatic transfer. For data acquisition, a 1D ^1^H NOESY pulse sequence (noesypr1d) was used as it is standard for NMR metabolomics and the recommended choice for MagMet-W.^68,70,79,80^ The activity was structured whereby a team of three Ph.D. students collaboratively led a discussion of data acquisition, processing, and analysis, while a research scientist provided a real-time showcase of the instrument in operation. This approach offered graduate students the chance to practice communicating science to nonexpert audiences while fostering professional identity as scientists.^91^ A real-time view of the workstation computer was projected onto a large screen to enable visualization of the TopSpin software, the resulting NMR spectra, and the data analysis in MagMet-W (Figure S3). Once the NMR data was collected, the raw “fid” files were uploaded to the MagMet-W webserver. MagMet-W automatically processes the NMR data, assigns each NMR peak to one of 70 molecules commonly found in wine, and estimates the concentration of each compound in micromolar (μM) (Figures S4 and S5). The area under the NMR peaks (i.e., the signal intensity) is proportional to the number of hydrogen nuclei such that it can be used to estimate concentration. To convert NMR peak areas into concentrations, MagMet-W uses the reference compound DSS as an internal calibrant.^68,70^ The MagMet-W Web server allows users to navigate which peaks correspond to which compound and to compare metabolite concentrations within a single wine sample. We then used in-house Python scripts to illustrate how to compare the chemical composition across different wine samples. Toward our core learning objectives (Figure 1), we first showed the chemical structures of a simple molecules like ethanol where we highlighted the hydrogen atoms in the molecule. We then linked that molecular structure directly to the signal in the ^1^H NMR spectrum. We illustrated examples of different molecular structures present in wine, highlighting how they are chemically distinct from ethanol, so they have unique NMR signals (PowerPoint). We explained how each compound contributes to wine characteristics (Figure S5). Second, we showed a zoomed out overlay of ^1^H NMR spectra of different wines with peaks corresponding to different compounds annotated (Figure 3, Figures S6–S8, Extended Analysis, and Supplementary Figures). These simple visual representations made it quite easy to understand the most important concepts even if participants do not fully understand how scientists can identify what the NMR peaks are and why NMR peaks show up where they do.

**Figure 3:**
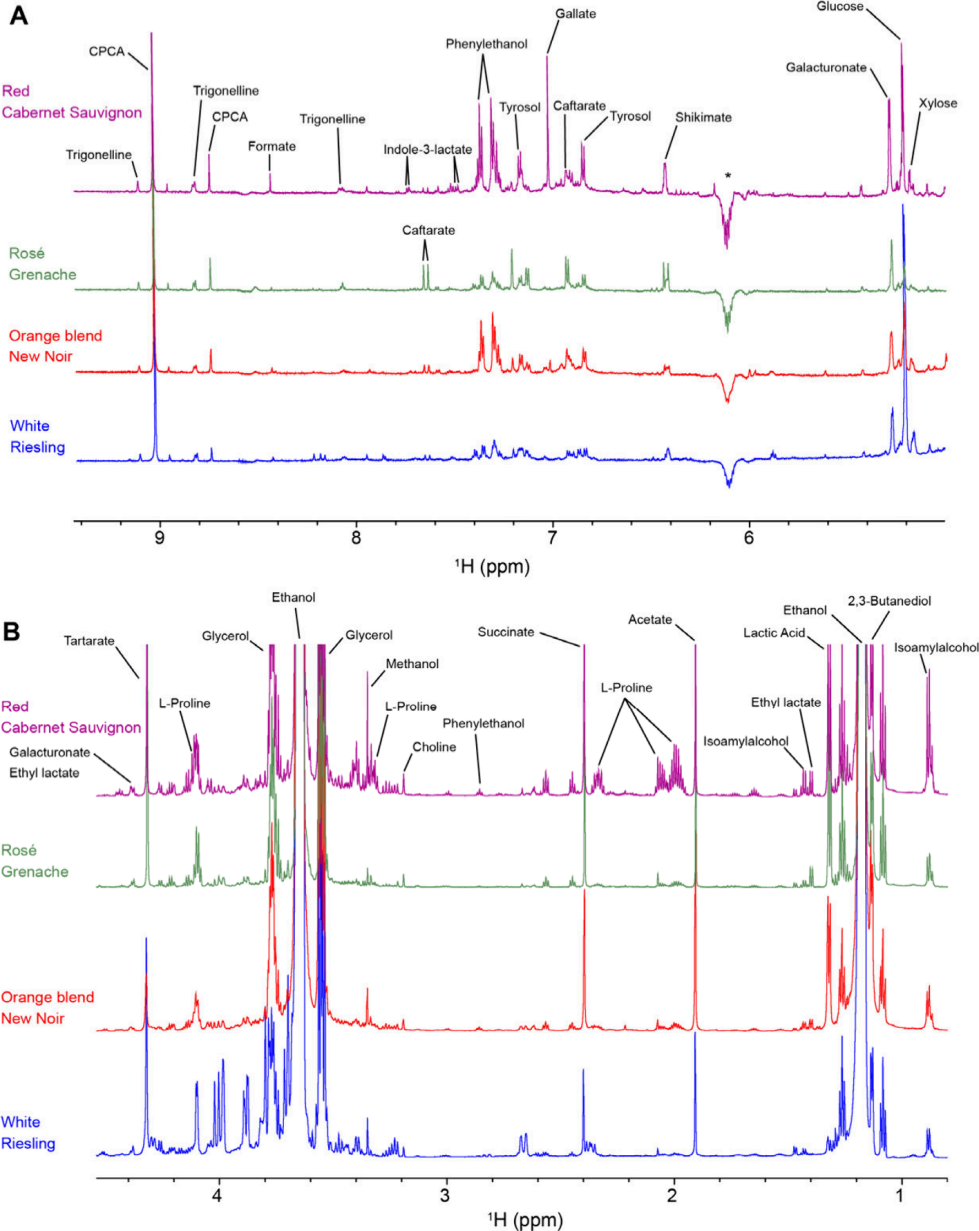
Comparison of 1D ^1^H NMR spectra across red, white, rose, and orange wine types. ^1^H NMR spectra (pulse sequence noesypr1d) of four different types of wines (red, rosé, orange blend, and white) recorded at a ^1^H field of 700 MHz at 25 °C. Spectral overlays are shown at the same intensity level (1:1 scale) zoomed into the (A) aromatic region and (B) aliphatic region. A subset of the MagMet-W identified peaks are annotated. The asterisks denote spectral artifacts.

### Identification of Wine Fraud and Adulteration

Next, we explained that due to its market value wine is frequently subjected to fraud and adulteration where harmful reagents are added as sweeteners, antimicrobials, or stability enhancers.^92,93^ We highlighted three historical examples of wine adulteration. First, the 1980s Austrian wine scandal where diethylene glycol, a toxic component of antifreeze, was added to enhance sweetness.^92,94,95^ Second, the 1980s Italian wine scandal where methanol, a cheaper alcohol than ethanol, was added to increase alcohol content, but resulted in blindness and death.^96,97^ Third, ancient Romans used a lead­(II) acetate-based syrup called *sapa* to sweeten and preserve wine, which resulted in lead poisoning that is hypothesized to be partially responsible for the collapse of the Roman Empire.^76,98,99^ Modern examples of unintentional wine adulteration were discussed, including residual pesticides^93,100,101^ and hazards associated with climate change.^102,103^ These highlighted a call for stringent wine testing and quality control efforts where NMR plays a key role. The historical aspects of wine fraud were presented in oral form by the graduate students, which was engaging and encouraged audience questions.^104^ PowerPoint fatigue was also avoided.^105^

Prior to the demonstration, we deliberately contaminated a Sauvignon Blanc wine with toxic levels of methanol,^74^ diethylene glycol,^75^ and lead­(II) acetate.^106^ Note that both methanol and acetate are naturally present in wine at nontoxic concentrations ranging from 500 to 3500 μM (methanol) and from 3000 to 7000 μM (acetate) (Figures S9 and S10). In unadulterated wine, methanol arises from the hydrolysis of pectins by pectinase enzymes, while acetate is a byproduct of yeast and bacterial metabolism during fermentation.^107^ Participants saw an overlay of three ^1^H NMR spectra: (1) the unadulterated Sauvignon Blanc, (2) the adulterated Sauvignon Blanc, and (3) the reference adulterants. To emphasize the real-world stakes, participants were assigned the role of scientific investigators. Participants were given a mission to save consumers by using the three ^1^H NMR spectra to identify the type of wine fraud before the adulterated wine was distributed. This mission-based framing enhances participant engagement by creating a sense of urgency and personal responsibility.^108^ Successful identification of adulterants was simplified by the presence of several NMR signals with limited chemical shift overlap with endogenous wine compounds: a singlet peak for methanol methyl protons at 3.35 ppm, a multiplet peak for diethylene glycol methylene protons at 3.73 ppm, and a singlet peak for lead­(II) acetate methyl protons at 1.9 ppm (Figure 4A and B and Figure S11).

**Figure 4:**
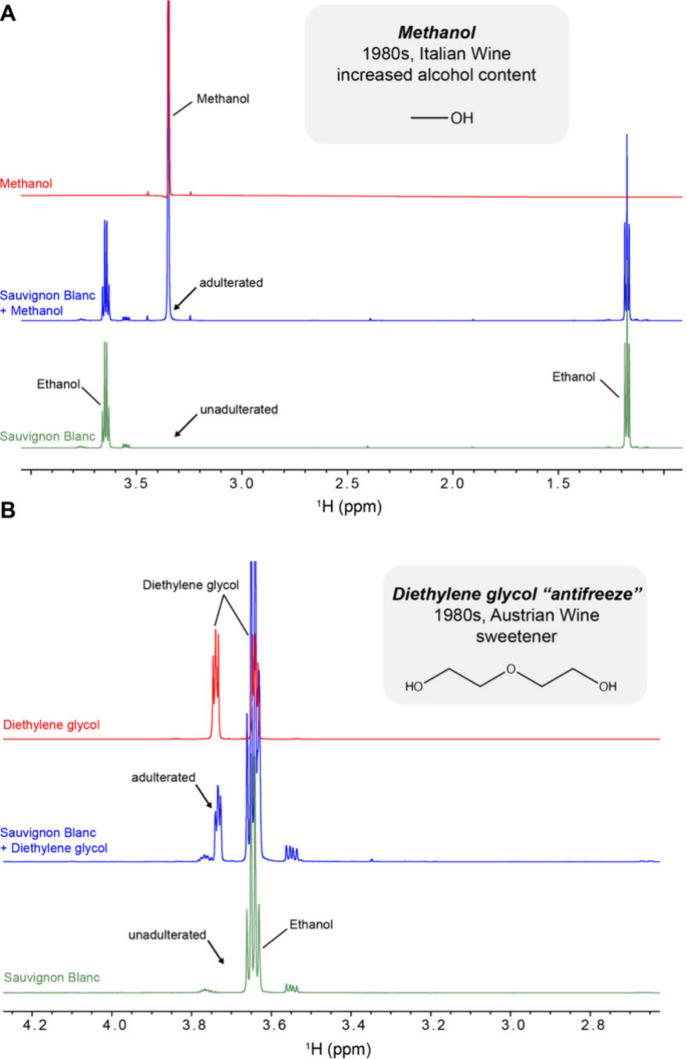
1D ^1^H NMR spectra of adulterated wines. ^1^H NMR spectra (pulse sequence noesypr1d) of reference adulterants (top, red), Sauvignon Blanc with the adulterants (blue), and Sauvignon Blanc without adulterants (bottom, green) for (A) 10% (v/v) methanol and (B) 10% (v/v) diethylene glycol recorded at a ^1^H field of 700 MHz at 25 °C. Spectral overlays are shown at the same intensity level zoomed into aliphatic region.

### Assessment of Learning and Public Excitement

Following the demonstration, participants left the NMR center where they were met by two additional Ph.D. students who assessed learning and excitement. Participants were given the choice to participate in a low-stakes assessment.^109^ Two summative assessments were developed (one for K–12 students and one for adults) with learning outcomes in mind (Learning Assessments). Assessments were not piloted, but were inspired by analogous assessments from other STEM outreach events.^36,110^ The assessments were implemented on pen and paper or online evaluations via QR codes to increase response rates.^111^ Two mechanisms were provided as motivators to complete the assessments. First, because promotional items enhance interest and participation in STEM, we illustrated, printed, and freely distributed in-house stickers that promote NMR research at Georgia Tech (Figure S12). Second, we distributed non-alcoholic wines and juices for additional sensory experiences. All K–12 participants attended under close supervision of the parents and were clearly told that wine is an alcoholic adult beverage. Most children were aware that they cannot drink alcoholic beverages, so this was not new information. Most parents allowed their child to do the smell test. Non-alcoholic wine choices matched wines tested in the demonstration. Adult participants appreciated the ability to share their favorite wines in a kid-friendly setting. Apart from the formal assessments, participants were also asked about their experience via informal word-of-mouth methods.^112,113^ Word-of-mouth methods are informal ways of gathering feedback that rely on low-stakes personal conversation rather than structured surveys, tests, or written assessments. The results learning assessments were quantified separately to compare impact across different age groups (Materials and Procedures).

Two multiple choice questions were given to K–12 participants: “How was the NMR instrument used to study wine today?” and “Which of the following chemicals can be found in many wines?”. Each question included both correct and incorrect options. K–12 participants identified 75% (3 of 4) or 100% (4 of 4) of the correct answers, suggesting some retention of NMR concepts and chemical components of wines (Figure 5A). Curiosity and excitement was assessed with the short response question “What do you think was the coolest or funnest part of the demo?” where answers were grouped into sensory, cognitive, misunderstanding, or unclear responses. K–12 excitement was primarily derived from sensory experiences (i.e., the sample being loaded into the spectrometer via the pneumatic flow apparatus, smell tests, the binder clip being pulled toward a magnetic field) or cognitive experiences (i.e., learning about the chemical components of wine) (Figure 5B).

**Figure 5:**
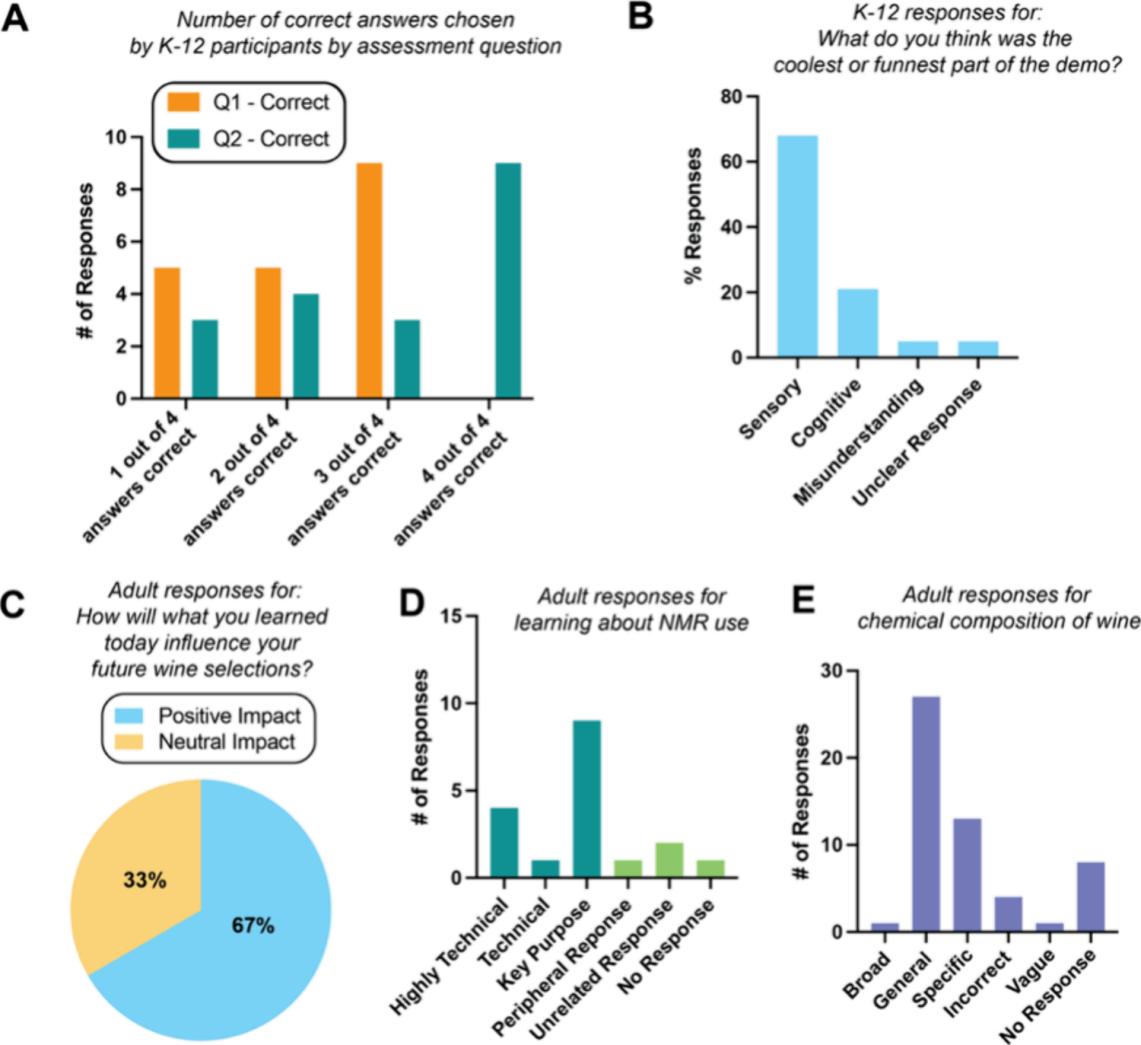
Results of assessing learning goals and outcomes for K–12 and adult participants. (A) Number of correct answers chosen by K–12 participants for multiple choice question 1 “How was the NMR instrument used to study wine today?” and question 2 “Which of the following chemicals can be found in many wines?” (B) Classification of the percentage responses by K–12 participants for the question “What do you think was the coolest or funnest part of the demo?” Answers were grouped into sensory, cognitive, misunderstanding, or unclear responses. (C) Quantification of positive or neutral impacts of the learning module by classification of adult responses to the question “How will what you learned today influence your future wine selections” (D) Classification of adult responses to the question “What did you learn about how NMR spectroscopy can be used to study wine?” Answers were grouped into highly technical, technical, key purposes, peripheral response, unrelated response, and no response. (E) Classification of adult responses to the question “List 3 chemical components of the wine you learned about from the profiling experiments”. Answers were grouped into broadly correct, general, specific, incorrect, vague, and no response (Materials and Procedures).

Example responses to the question “What do you think was the coolest or funnest part of the demo” included

- “the magnet and the paper clip” [elementary school age]
- “the magnet next to the clip” [middle school age]
- “seeing the big magnet” [middle school age]
- “watching the machine work” [middle school age]
- “understanding the process of determining the components of wine” [high school age]
- “learning about the superconductor and liquid helium” [high school age]

Adult curiosity and excitement was assessed with the short response question “How will what you learned today influence your future wine selections” where 67% of respondents were positive (Figure 5C). Learning of NMR in research was probed with the short response question “What did you learn about how NMR spectroscopy can be used to study wine?” where answers were grouped into highly technical, technical, key purposes, peripheral response, unrelated response, and no response categories, with 78% of responses being highly technical, technical, or serving key purposes (Figure 5D). Finally, learning of the chemical components of wine was assessed with the short answer question “List 3 chemical components of the wine you learned about from the profiling experiments” where answers were grouped into broadly correct, general, specific, incorrect, vague, and no response categories, with 74% of responses being general (i.e., alcohols, sugars, and amino acids) or specific (i.e., ethanol, fructose, and l-proline) (Figure 5E).

Example responses to the question “How will you learned today influence your future wine selections” included

- “It made me feel good about my current selection since its higher in amino acids and lower in sugar and ethanol”
- “I don’t drink, but next time I see my parents with a glass of wine, I’ll start spitting facts about ancient Rome and wine poisoning”
- “It was neat to see that wines grown in areas with drought produced more antioxidants”
- “I now understand better why red wines have more polyphenols than white wines”

## Discussion

### Practical Considerations, Perspectives, and Future Efforts

One consideration in either an outreach or coursework setting is sample preparation and data acquisition time. Here, samples were prepared, acquired, and processed beforehand to allow real-time demonstration (∼30–45 min per group). However, in a university coursework setting, instructors can purchase a selection of wines and provide prelab lectures (or prelab assignments) that enable students to develop testable hypotheses. For example, students with red wine may hypothesize the presence of higher concentrations of polyphenol compounds that impart color and aroma,^114^ while students with white wines may hypothesize higher concentrations of organic acids that impart enhanced acidity.^115^ Sample preparation is estimated at 15–30 min, and multiple samples can be prepared simultaneously. Data collection for a single wine takes 10 min of setup time plus 15 min for data acquisition. The runtime of MagMet-W is 7 min. The Python-based automated processing scripts and multivariate analyses can be performed in less than 10 min. The workflow is affordable. A bottle of wine costs on average ∼ $15 USD.^116^ At our university, the 700 MHz NMR spectrometer time is $5 per wine sample. All software used here for data processing are free and require only a Python-compatible computer.

Delivery of the module was challenged by making sure it was accessible to different age groups from K–12 to adults with different backgrounds. Participants under the age of 13 had some difficulties engaging fully with the demonstration. For example, none of the younger participants were able to identify all four of the ways that NMR was used to study wine. For the question “Which of the following chemicals can be found in many wines?”, younger participants always chose the incorrect answer for inorganic acids, such as hydrochloric acid. Since other chemicals with names containing the word “acid” are found in wines, younger participants struggle with discerning differences. However, most participants exhibited excitement upon seeing NMR instruments function and meeting scientists. The learning assessments seemed to be able to measure general knowledge retention and engagement by combining multiple-choice questions to assess factual recall with short-answer questions to capture interest. However, since the assessments were not piloted, results may be biased; future use of pre/post-tests and rubrics could improve reliability.

### Requirements for Replication and Limitations

We recommend instructors familiarize themselves with (1) Moreno-Arribas et al’s review of the biochemistry of wine making,^49^ (2) Moore et al’s introduction to food fraud,^92^ (3) Huang et al’s review on NMR to study complex mixtures,^63^ and (4) Lee et al’s implementation of MagMet-W.^68^ If materials are limited, one wine from each class would be sufficient to achieve the learning objectives (Figure 3). Commonly available wines are Provence rosé, Chardonnay white, and Cabernet Sauvignon red. All procedures and software are freely available and outlined here for replication (Tutorial). To further support reproducibility, we created a YouTube video that highlights what chemical educators need to do to implement the demonstration (Data Availability section). YouTube videos are pedagogical aids in STEM education, where they increase content accessibility, visualize abstract concepts, and enhance student engagement.^117,118^ The video could be shown to the public (in an outreach setting) or students (in a coursework setting) to give participants a stronger background knowledge. The video also provides additional opportunities to educate the public.

One limitation is instrument availability. MagMet-W requires data acquisition on a high-field 700 MHz spectrometer not available at all universities, especially primarily undergraduate institutions.^68^ The authors of MagMet-W note that they are working on a future implementation suitable with lower field spectrometers.^68^ We attempt to alleviate this limitation by providing raw NMR data and MagMet-W processed data (Data Availability section). This will allow instructors to adapt the module for course work or outreach events even in the absence of resources. A second limitation is incomplete profiling due to incompleteness of the MagMet-W compound library. This is not expected to be a major limitation since the majority of the NMR peaks present in wines were identified by MagMet-W (Figure S4).

In the absence of a high-field 700 MHz spectrometer, the module could be implemented as follows: (1) participants visit any available NMR magnet, (2) participants interact with students, research staff, or a faculty member, who highlight the role of NMR in research, (3) participants view a NMR spectrometer working in action, and (4) participants see examples of NMR data and learn how they inform a better understanding of health and everyday life. The data and analysis can come from their measurements, or use data from the in this article. The demonstration can be easily modified for the characterization of other samples of public interest, such as honey, therapeutic drugs, and olive oil.^119–121^

## Supplementary Material















## Data Availability

All 1D ^1^H NMR Bruker raw “fid” data obtained in this study, MagMet-W processed data, and Python scripts to analyze the MagMet-W data are freely available at GitHub (https://github.com/mcshanlab/Capeci_et-al_WineNMR_2025_GitHub). All learning assessment data were collected anonymously with informed consent of the participants. A YouTube video detailing how to perform the sample preparation, data acquisition, and data analysis is available (https://www.youtube.com/watch?v=9_QPgV14mbs).

## Notes

Supplementary Figures (PDF)
Learning Assessments (PDF)
Extended Analysis (PDF)
Materials and Procedures (PDF)
Simplified NMR Theory (PDF)
Tutorial (PDF)
PowerPoint (PDF)
