# Supplementary material for: Automated Chemical Profiling of Wine by Solution NMR Spectroscopy: A Demonstration for Outreach and Education
Source: J Chem Educ. 2026 Jan 6;103(2):833–45. doi: 10.1021/acs.jchemed.5c00652 (PMC12895419; doi:10.1021/acs.jchemed.5c00652)
Supplement: Supplementary file 1 [file ed5c00652_si_001.pdf]

Supplementary Information for

Automated Chemical Profiling of Wine by Solution NMR Spectroscopy: A Demonstration for Outreach and Education

Lily Capeci[1, ‡], Ruoqing Jia[1, ‡], Mary E. Peek[1], Miriam K. Simma[1], Elizabeth A. Corbin[1], FNU Vidya[1], Hongwei Wu[1*], Johannes E. Leisen[1*], Andrew C. McShan[1*]

‡These authors contributed equally to this work.

[1]School of Chemistry and Biochemistry, Georgia Institute of Technology, Atlanta, GA 30332, USA

*Correspondence: Hongwei Wu (hongwei.wu@chemistry.gatech.edu), Johannes E. Leisen (johannes.leisen@chemistry.gatech.edu), and Andrew C. McShan (andrew.mcshan@chemistry.gatech.edu)

Supplementary Figures:

The supplementary file contains Supplementary Figures cited in the main text.

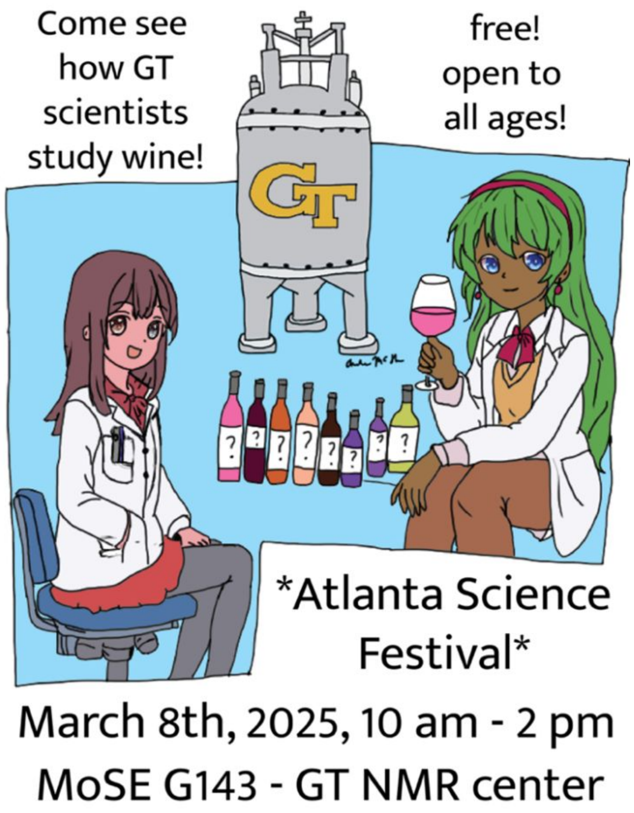

Supplementary Figure 1. Promotional flyer used for the outreach event. Image credit: Andrew McShan. You may modify and redistribute freely.

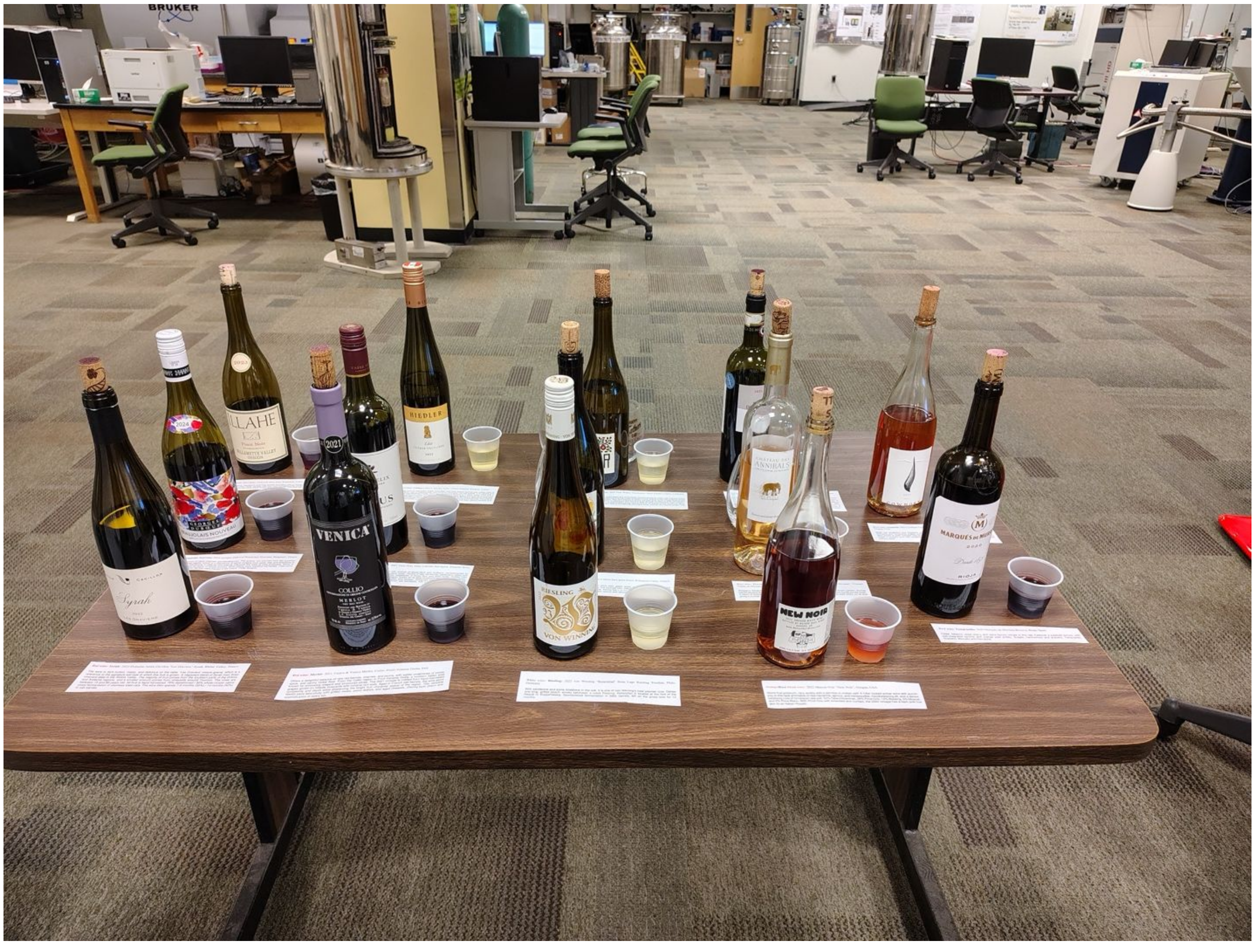

Supplementary Figure 2. Example setup for the wine visualization, history lesson, and smell tests. A total of 14 different wines are shown covering red, rosé, white, and orange wine. Next to each wine is a sample of the wine where participants can (1) visualize the color of each wine and (2) pick up and directly smell the aroma of wine. Since the learning module was implemented in a chemistry lab, no tasting was allowed. Also next to each wine is a short statement about the name, grape cultivar, and wine terroir.

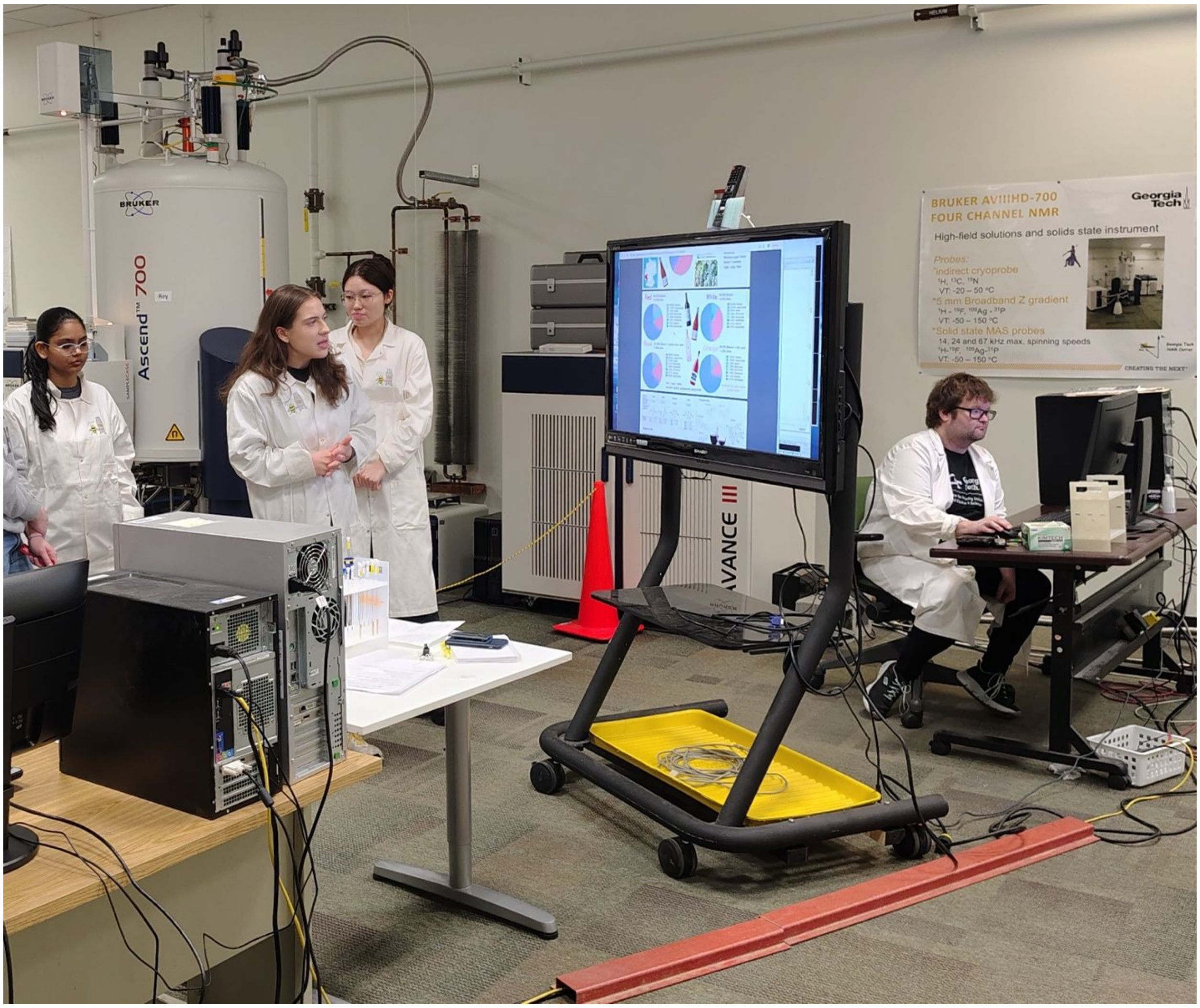

Supplementary Figure 3. Example setup of the NMR data acquisition and real-time visualization of results. Left: Biochemistry Ph.D. students Ms. Vidya, Ms. Capeci, and Ms. Jia leading a discussion of the results. Middle: A projector connected to the spectrometer workstation mirrors the view of the computer allowing for ease of visualization. Right: Dr. McShan acquiring data and analyzing results in real-time at the spectrometer workstation. Public participants are not shown to protect their identity.

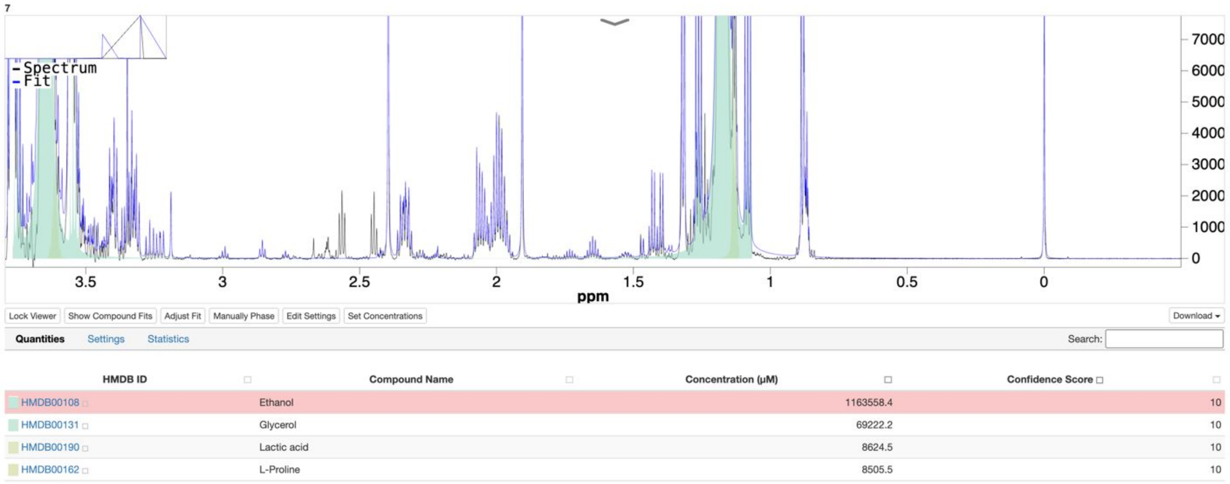

Supplementary Figure 4. Example view of the MagMet-W results window for red wine Cabernet Sauvignon. Top: JSpectraViewer of the Fourier transformed/processed FID (black) versus the MagMet-W fitted spectra (blue). Most peaks are identified and well-fitted. Several NMR peaks are not identified (see region from ~2.48 to 2.6 ppm). The ethanol NMR peaks are highlighted on the screen since the ethanol compound name is selected below. Bottom: Results from the "Quantities tab" that shows a list of Human Metabolome Database (HMDB) accession IDs, Compound Names, MagMet-W determined concentrations (μM), and MagMet-W determined confidence scores (0 to 10 with 10 being highly confident). Note that we observed that several of the HMDB accession numbers link to incorrect entries in the HMDB.

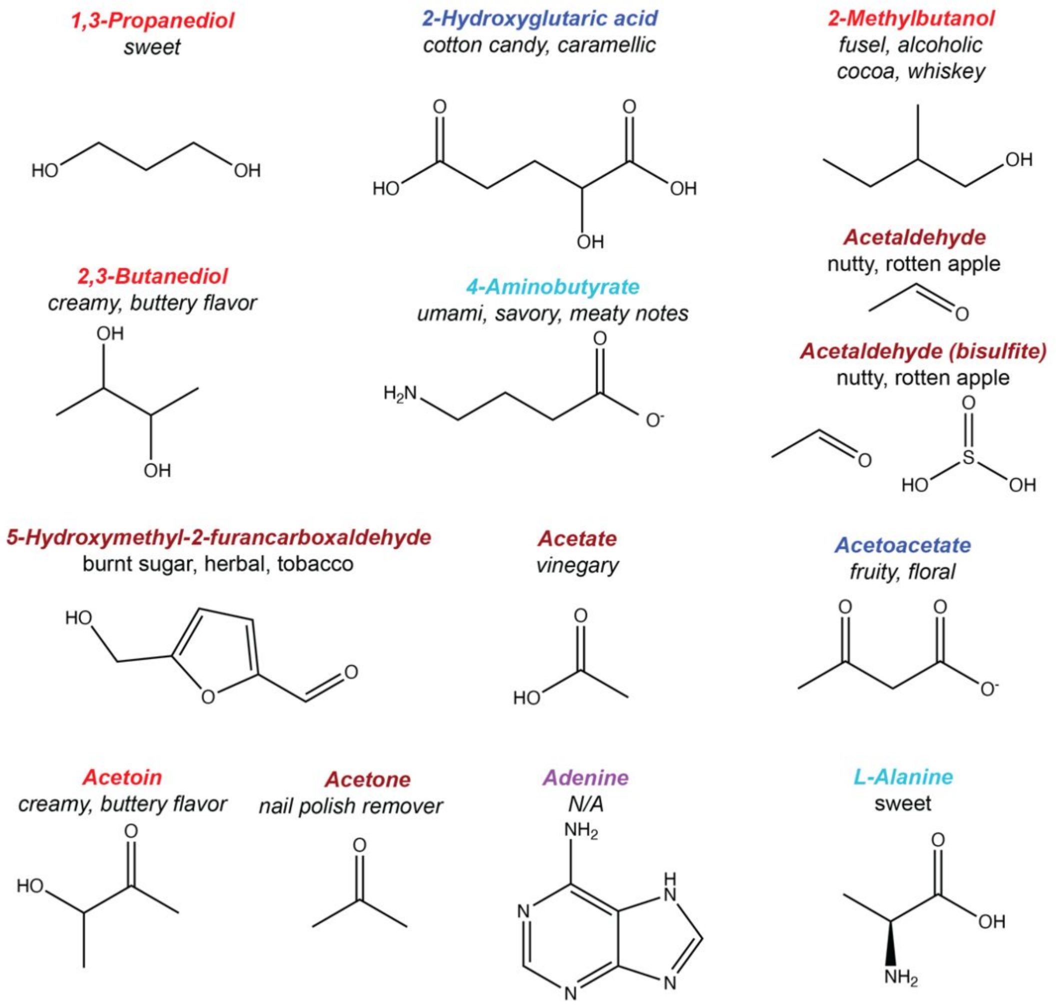

Supplementary Figure 5. Structures of compounds from the MagMet-W Library. Compounds have names colored by class: alcohols in orange, organic acids in blue, amino acids and amino acid derivatives in cyan, wine “faults” in brown, nucleobases in purple, sugars in green, polyphenols in pink, and chemical shift standards/phasing compounds in black. The contributions to wine flavor / aroma profile are noted. N/A = not applicable.

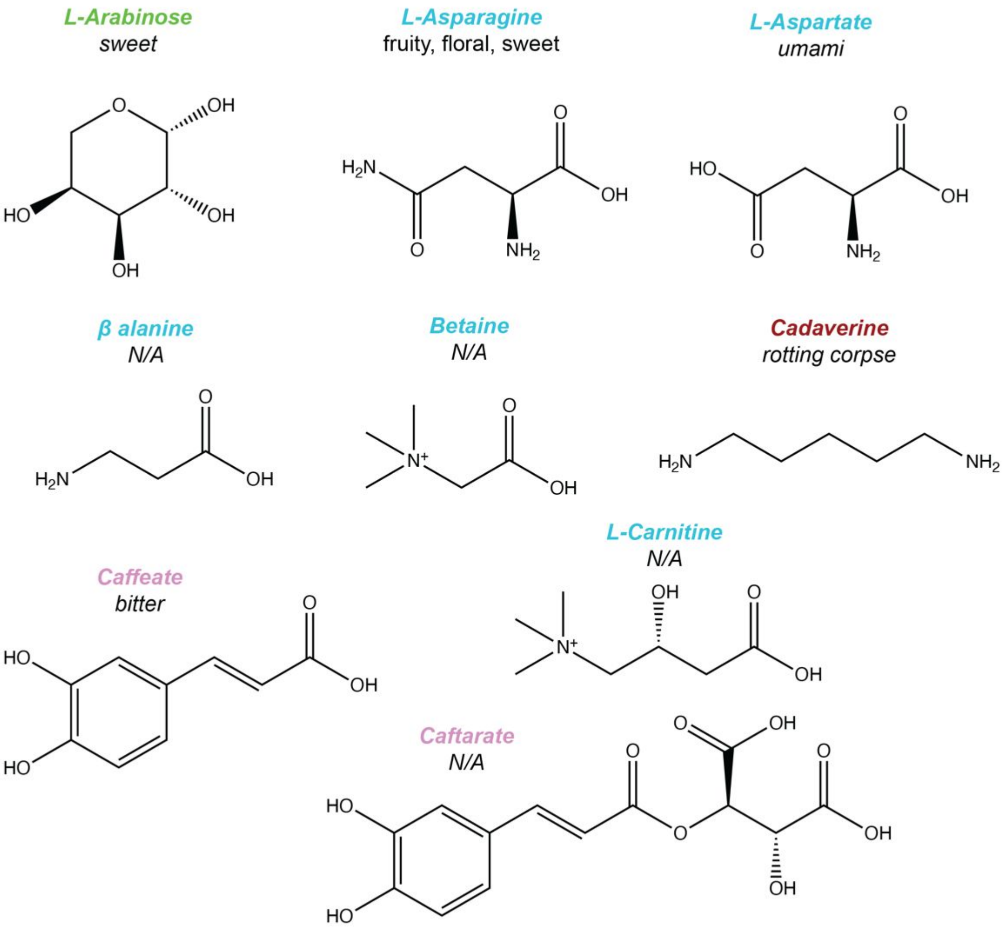

Supplementary Figure 5 cont. Structures of compounds from the MagMet-W Library. Compounds have names colored by class: alcohols in orange, organic acids in blue, amino acids and amino acid derivatives in cyan, wine “faults” in brown, nucleobases in purple, sugars in green, polyphenols in pink, and chemical shift standards/phasing compounds in black. The contributions to wine flavor / aroma profile are noted. N/A = not applicable.

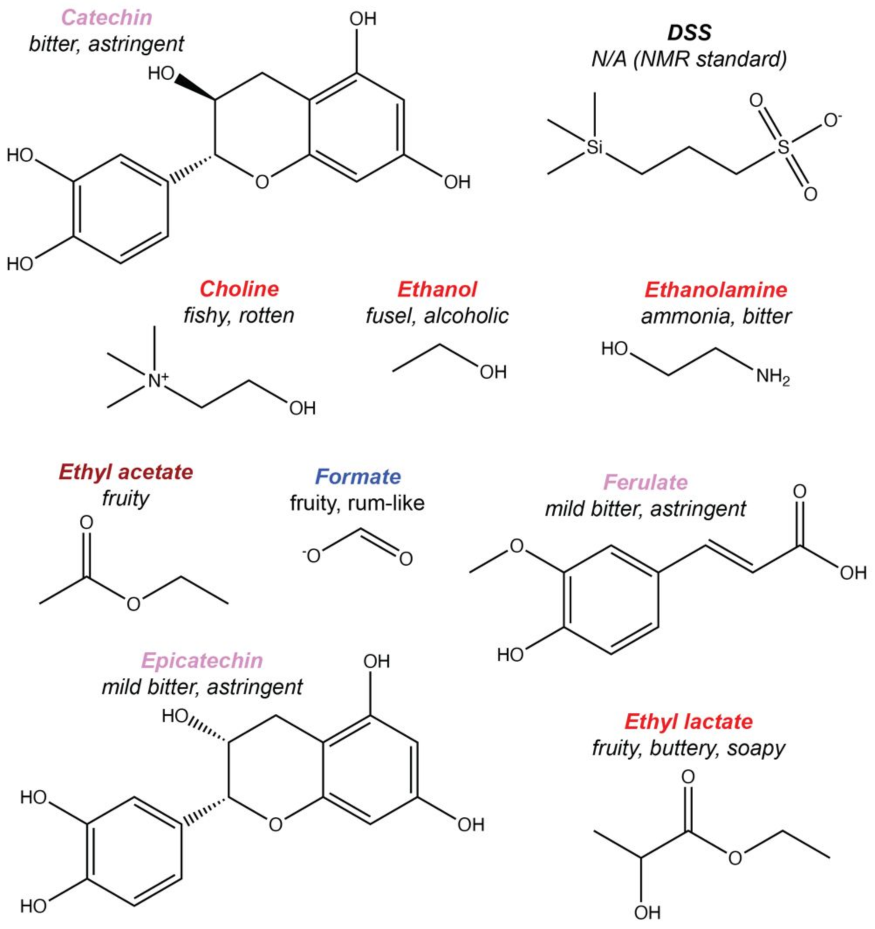

Supplementary Figure 5 cont. Structures of compounds from the MagMet-W Library. Compounds have names colored by class: alcohols in orange, organic acids in blue, amino acids and amino acid derivatives in cyan, wine "faults" in brown, nucleobases in purple, sugars in green, polyphenols in pink, and chemical shift standards/phasing compounds in black. The contributions to wine flavor / aroma profile are noted. N/A = not applicable.

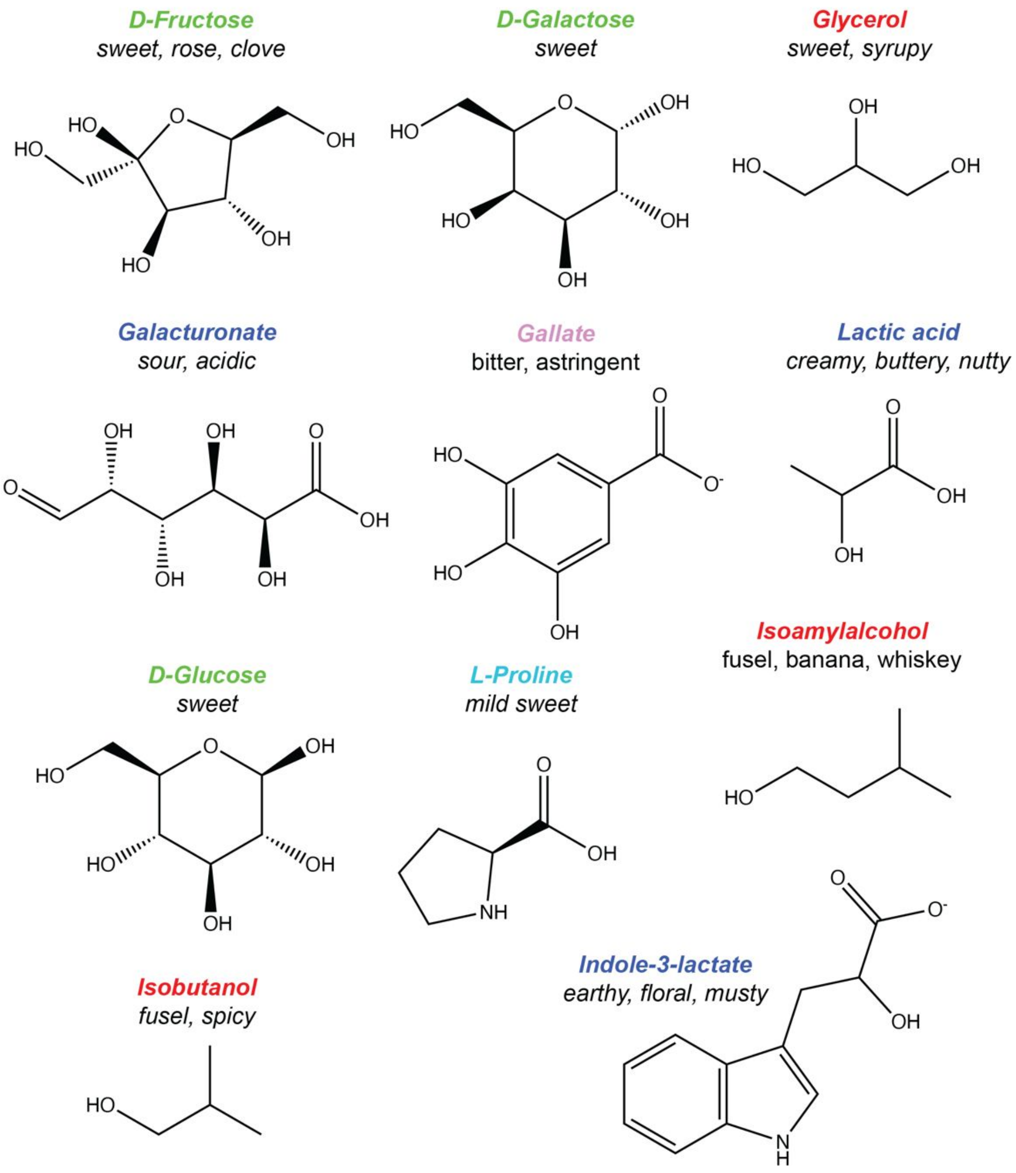

Supplementary Figure 5 cont. Structures of compounds from the MagMet-W Library. Compounds have names colored by class: alcohols in orange, organic acids in blue, amino acids and amino acid derivatives in cyan, wine "faults" in brown, nucleobases in purple, sugars in green, polyphenols in pink, and chemical shift standards/phasing compounds in black. The contributions to wine flavor / aroma profile are noted. N/A = not applicable.

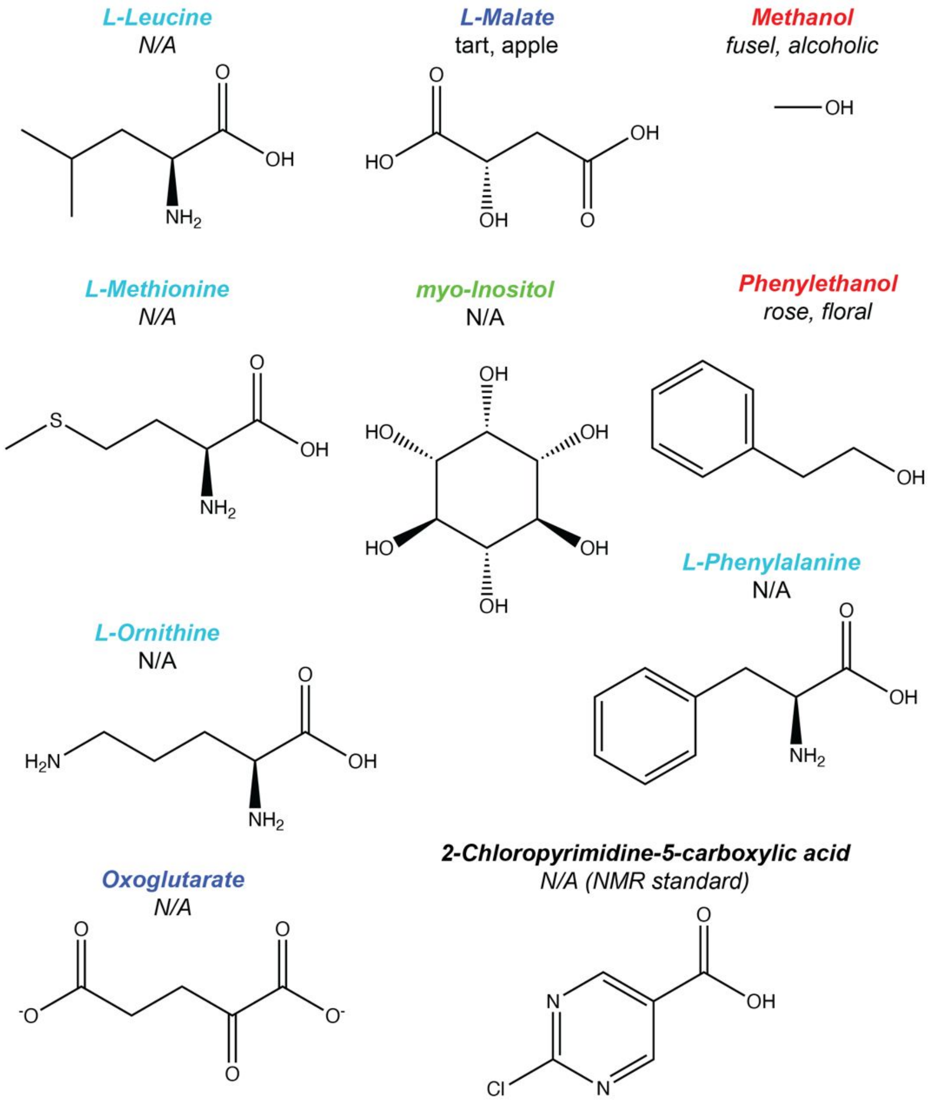

Supplementary Figure 5 cont. Structures of compounds from the MagMet-W Library. Compounds have names colored by class: alcohols in orange, organic acids in blue, amino acids and amino acid derivatives in cyan, wine "faults" in brown, nucleobases in purple, sugars in green, polyphenols in pink, and chemical shift standards/phasing compounds in black. The contributions to wine flavor / aroma profile are noted. N/A = not applicable.

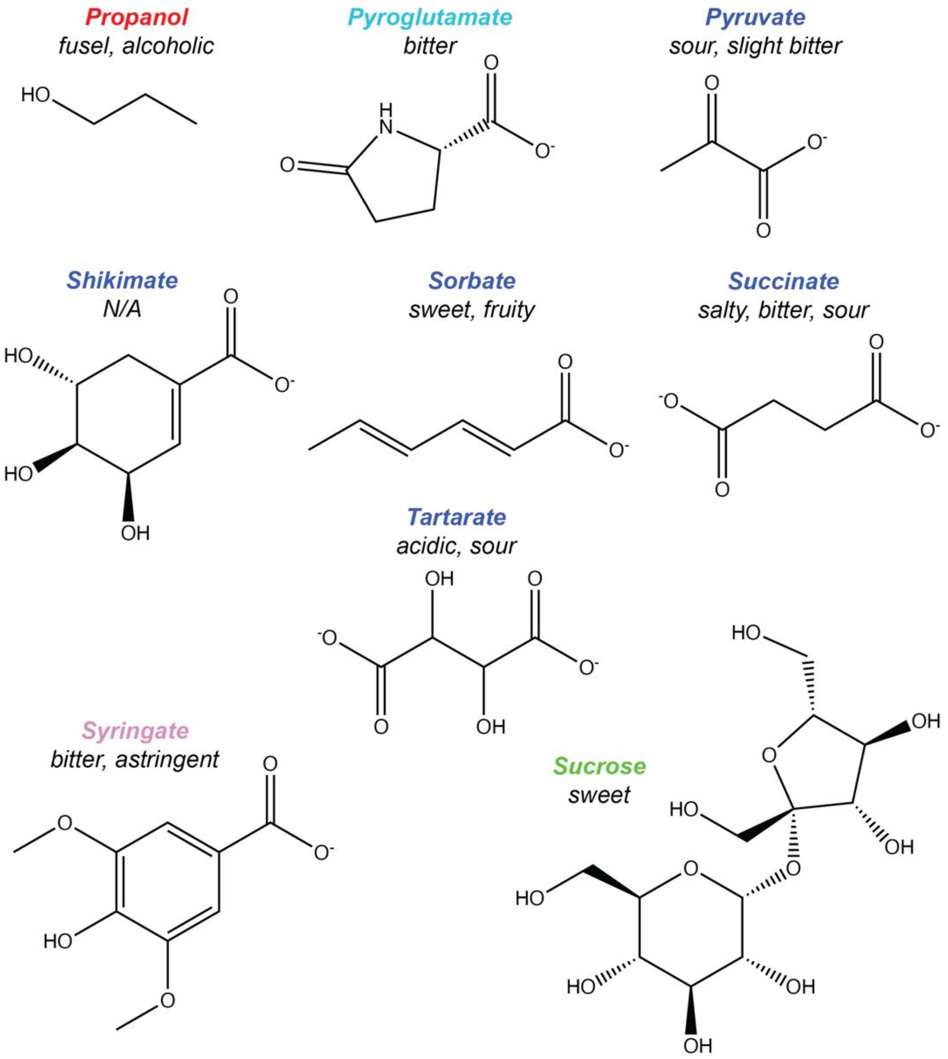

Supplementary Figure 5 cont. Structures of compounds from the MagMet-W Library. Compounds have names colored by class: alcohols in orange, organic acids in blue, amino acids and amino acid derivatives in cyan, wine "faults" in brown, nucleobases in purple, sugars in green, polyphenols in pink, and chemical shift standards/phasing compounds in black. The contributions to wine flavor / aroma profile are noted. N/A = not applicable.

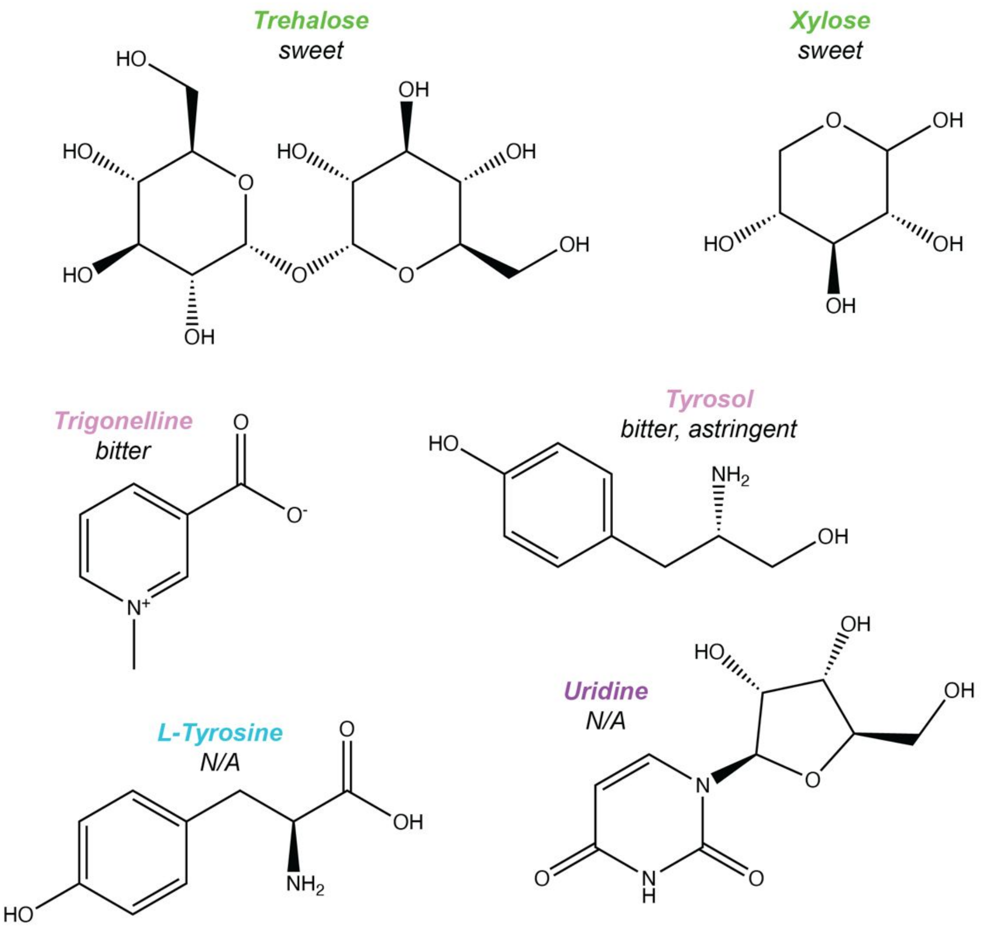

Supplementary Figure 5 cont. Structures of compounds from the MagMet-W Library. Compounds have names colored by class: alcohols in orange, organic acids in blue, amino acids and amino acid derivatives in cyan, wine "faults" in brown, nucleobases in purple, sugars in green, polyphenols in pink, and chemical shift standards/phasing compounds in black. The contributions to wine flavor / aroma profile are noted. N/A = not applicable.

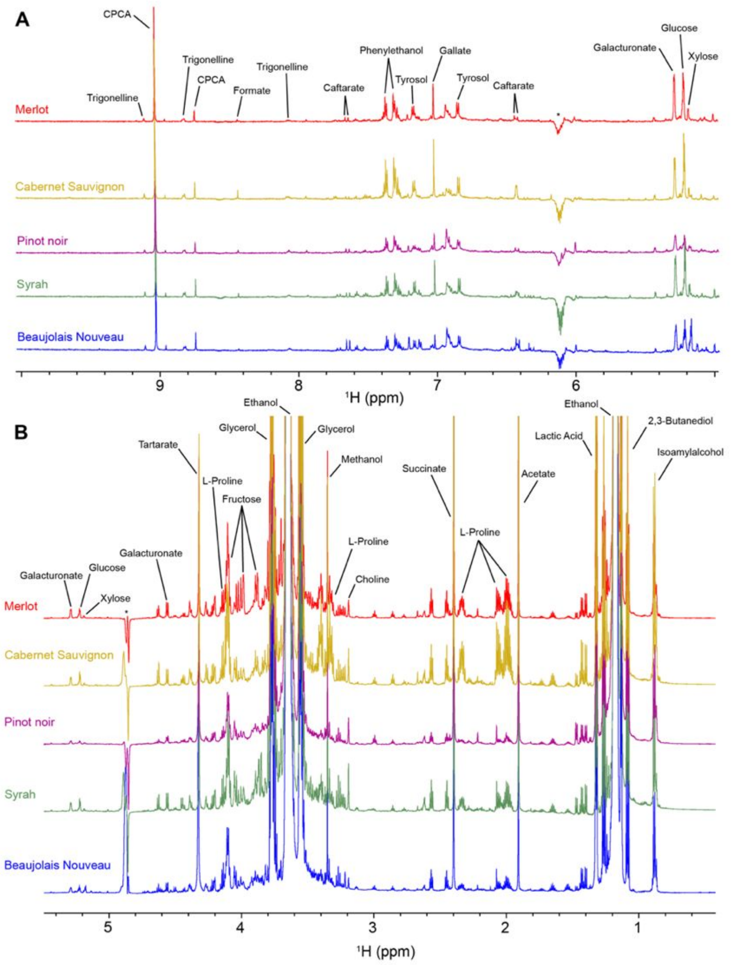

Supplementary Figure 6. Example comparison of 1D ^{1}H NMR spectra across red wine types. ^{1}H NMR spectra (pulse sequence noesypr1d) of five different red wines recorded at a ^{1}H field of 700 MHz at 25 °C. Spectral overlays are shown at the same intensity level (1:1 scale) zoomed into the (A) aromatic region and (B) aliphatic region. A subset of the MagMet-W identified NMR peaks are annotated. The asterisks denote spectral artifacts.

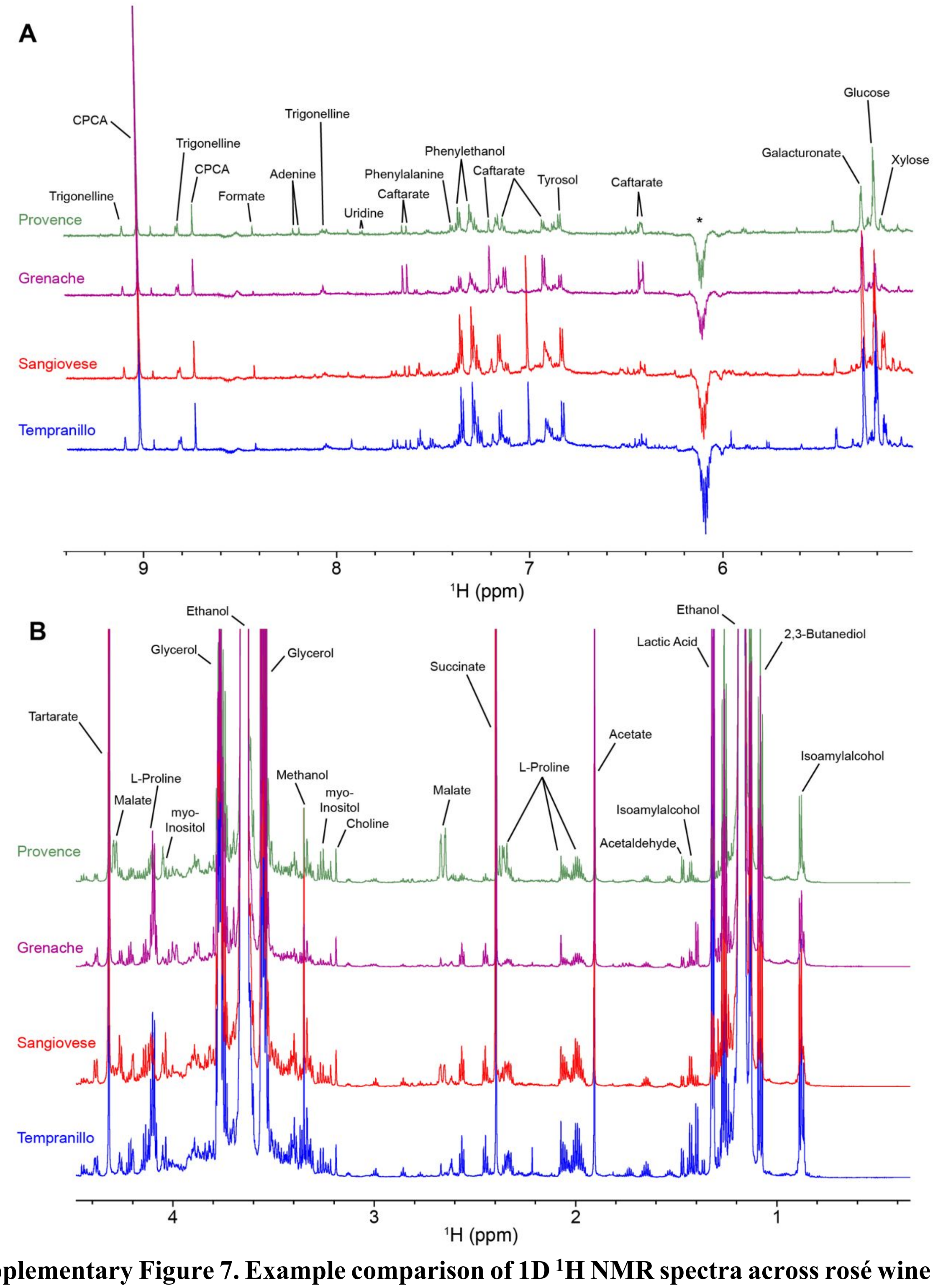

Supplementary Figure 7. Example comparison of 1D ^{1}H NMR spectra across rosé wine types. ^{1}H NMR spectra (pulse sequence noesypr1d) of five different rosé wines recorded at a ^{1}H field of 700 MHz. Spectral overlays are shown at the same intensity level (1:1 scale) zoomed into the (A) aromatic region and (B) aliphatic region. A subset of the MagMet-W identified NMR peaks are annotated. The asterisks denote spectral artifacts

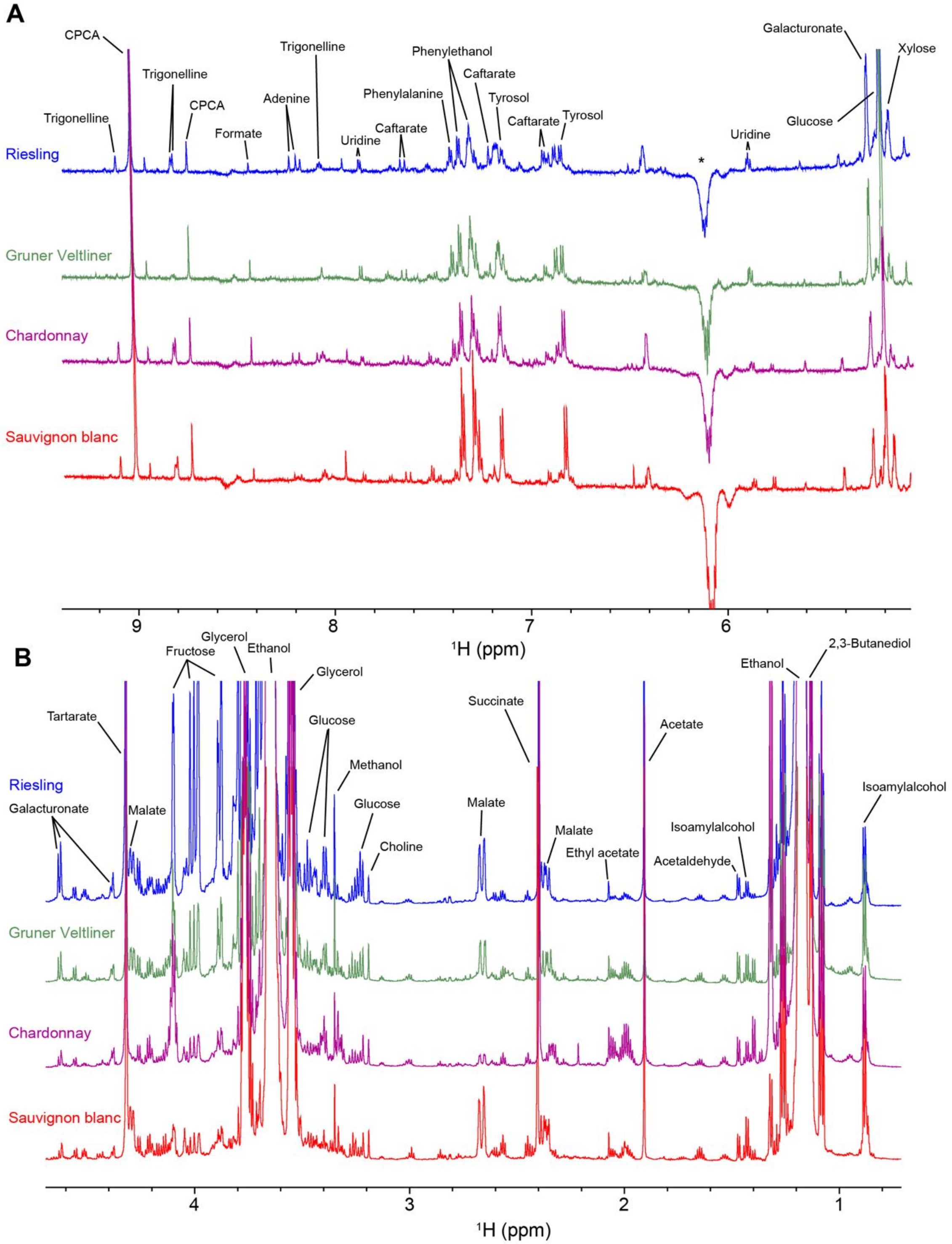

Supplementary Figure 8. Example comparison of 1D ^{1}H NMR spectra across white wine types. ^{1}H NMR spectra (pulse sequence noesypr1d) of five different white wines recorded at a ^{1}H field of 700 MHz. Spectral overlays are shown at the same intensity level (1:1 scale) zoomed into the (A) aromatic region and (B) aliphatic region. A subset of the MagMet-W identified NMR peaks are annotated. The asterisks denote spectral artifacts.

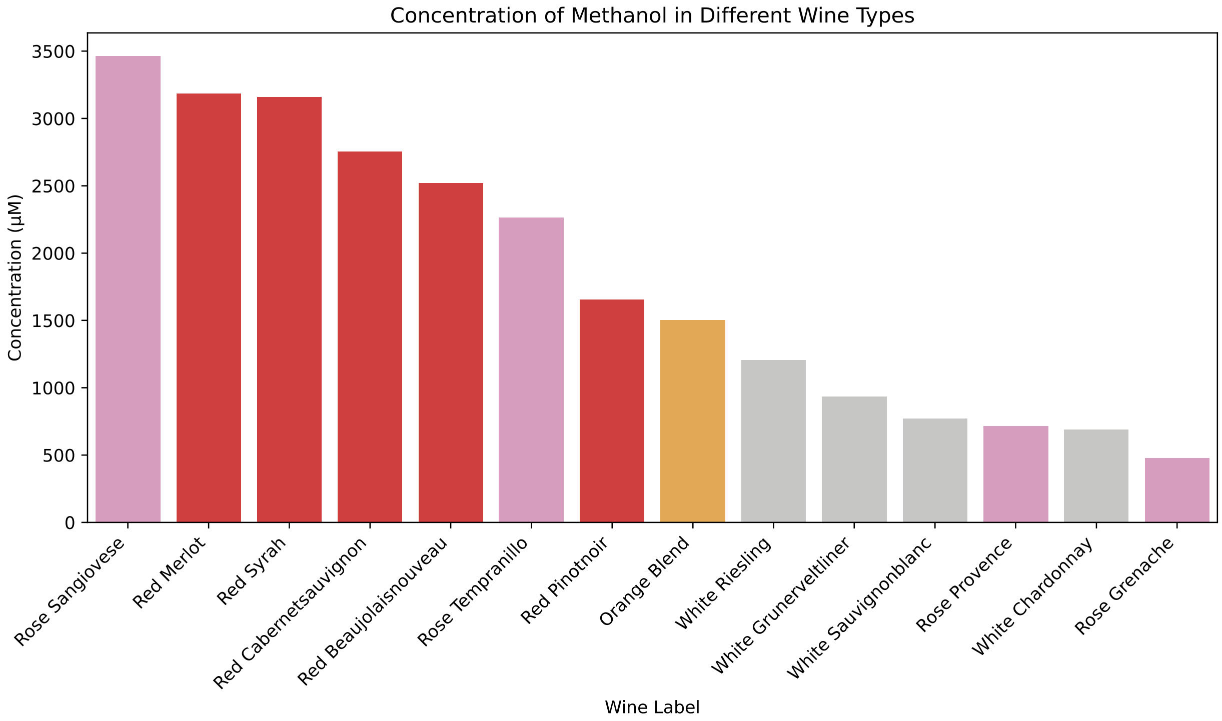

Supplementary Figure 9. Comparison of methanol concentrations obtained from MagMet-W across wines. The bar charts are generated from the Python 3 script bar-graph_one-compound_wine_analysis_from_MagMetW.py

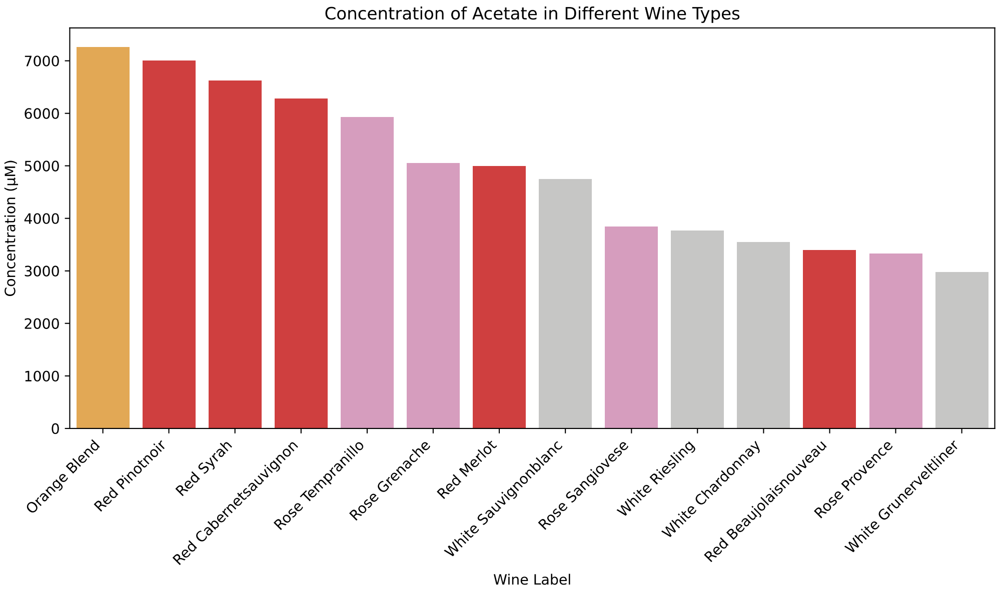

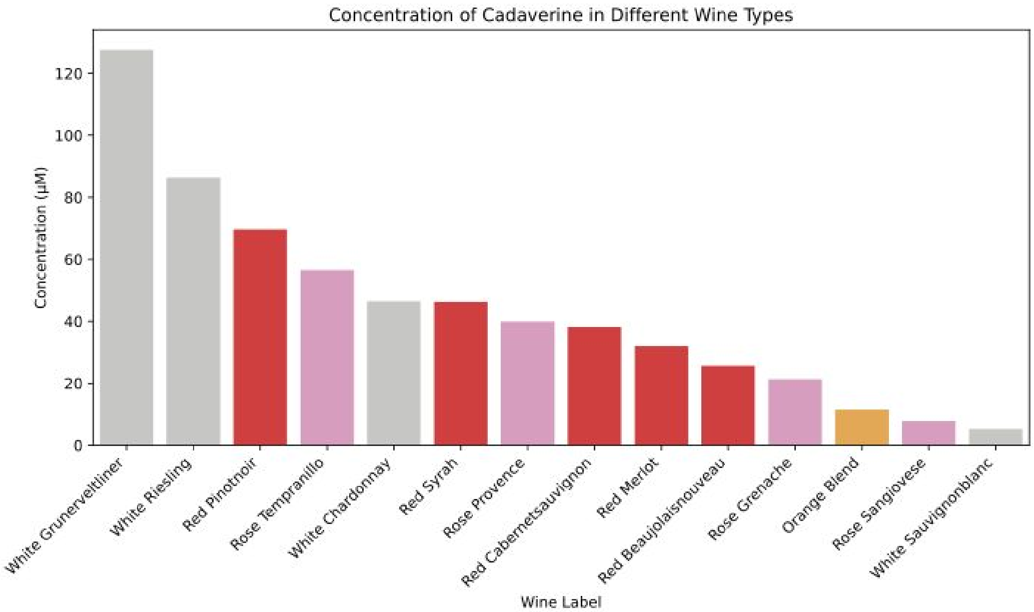

Supplementary Figure 10. Comparison of wine fault (acetate and cadaverine) concentrations obtained from MagMet-W across wines. The bar charts are generated from the Python 3 script bar-graph_one-compound_wine_analysis_from_MagMetW.py

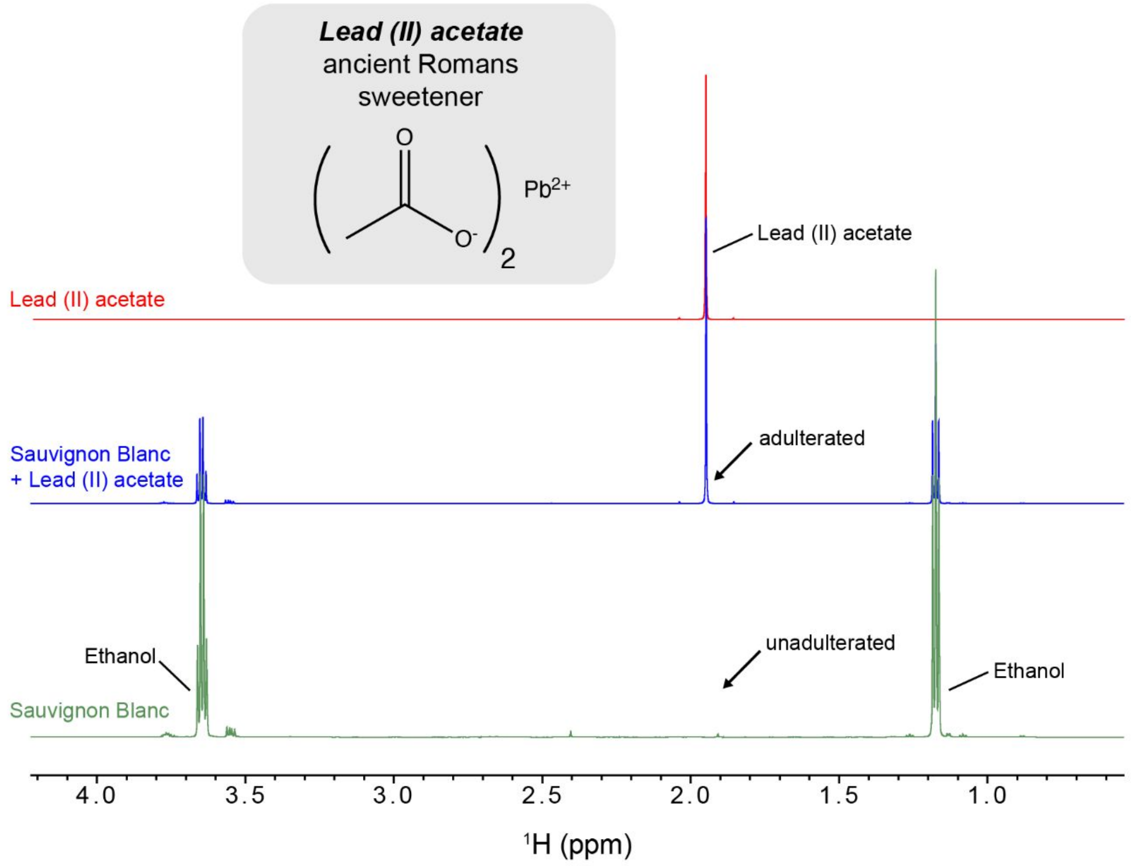

Supplementary Figure 11. 1D ^{1}H NMR spectra of adulterated wines. ^{1}H NMR spectra (pulse sequence noesypr1d) of reference adulterants (top, red), Sauvignon Blanc with the adulterants (blue), and Sauvignon Blanc without adulterants (bottom, green) for 80 mg/L lead (II) acetate recorded at a ^{1}H field of 700 MHz. Spectral overlays are shown at the same intensity level zoomed into the aliphatic region.

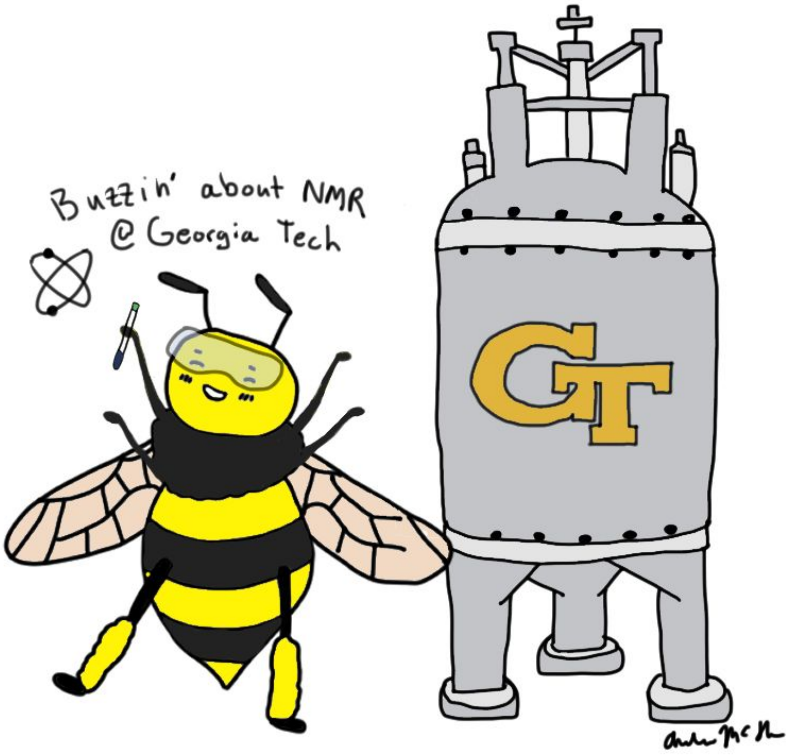

Supplementary Figure 12. Promotional sticker used for the outreach event. Image credit: Andrew McShan. You may modify and redistribute freely.
