# Supplementary material for: Automated Chemical Profiling of Wine by Solution NMR Spectroscopy: A Demonstration for Outreach and Education
Source: J Chem Educ. 2026 Jan 6;103(2):833–45. doi: 10.1021/acs.jchemed.5c00652 (PMC12895419; doi:10.1021/acs.jchemed.5c00652)
Supplement: Supplementary file 2 [file ed5c00652_si_003.pdf]

Supplementary Information for

Automated Chemical Profiling of Wine by Solution NMR Spectroscopy: A Demonstration for Outreach and Education

Lily Capeci[1, ‡], Ruoqing Jia[1, ‡], Mary E. Peek[1], Miriam K. Simma[1], Elizabeth A. Corbin[1], FNU Vidya[1], Hongwei Wu[1*], Johannes E. Leisen[1*], Andrew C. McShan[1*]

[‡]These authors contributed equally to this work.

[1]School of Chemistry and Biochemistry, Georgia Institute of Technology, Atlanta, GA 30332, USA

*Correspondence: Hongwei Wu (hongwei.wu@chemistry.gatech.edu), Johannes E. Leisen (johannes.leisen@chemistry.gatech.edu), and Andrew C. McShan (andrew.mcshan@chemistry.gatech.edu)

Learning Assessments:

The supplementary file contains the learning assessments used for the demonstration.

Learning Assessment for Outreach
Wine Profiling by NMR – Ages 18 and older

<u>Note:</u> Data collected is anonymous
and will be used for research purposes.

1. What did you learn about how NMR spectroscopy can be used to study wine?

2. List 3 chemical components of the wine you learned about from the profiling experiments:

a. ____

b. ____

c. ____

3. How will what you learned today influence your future wine selections?

Learning Assessment for Outreach
Wine Profiling by NMR – Ages 17 and younger

<u>Note:</u> Data collected is anonymous
and will be used for research purposes (with parental support).

1. **Circle all that apply**: how was the NMR instrument used to study wine today?
   a. to classify wines by region and wine type
   b. to determine the pH of different wines
   c. to understand the chemical components of wine that give it its flavor, aroma, and stability
   d. to determine the cell counts in different wines
   e. to check for wine quality and chemical contamination

2. **Circle all that apply**: which of the following chemicals can be found in many wines?
   a. inorganic acids, such as hydrochloric acid
   b. alcohols, such as ethanol
   c. sugars, such as glucose
   d. amino acids, such as proline
   e. organic acids, such as acetate
   f. organic solvents, such as chloroform

3. Short answer: What do you think was the coolest or funnest part of the demo?
