# Supplementary material for: Automated Chemical Profiling of Wine by Solution NMR Spectroscopy: A Demonstration for Outreach and Education
Source: J Chem Educ. 2026 Jan 6;103(2):833–45. doi: 10.1021/acs.jchemed.5c00652 (PMC12895419; doi:10.1021/acs.jchemed.5c00652)
Supplement: Supplementary file 3 [file ed5c00652_si_005.pdf]

Supplementary Information for

Automated Chemical Profiling of Wine by Solution NMR Spectroscopy: A Demonstration for Outreach and Education

Lily Capeci[1, ‡], Ruoqing Jia[1, ‡], Mary E. Peek[1], Miriam K. Simma[1], Elizabeth A. Corbin[1], FNU Vidya[1], Hongwei Wu[1*], Johannes E. Leisen[1*], Andrew C. McShan[1*]

[‡]These authors contributed equally to this work.

[1]School of Chemistry and Biochemistry, Georgia Institute of Technology, Atlanta, GA 30332, USA

*Correspondence: Hongwei Wu (hongwei.wu@chemistry.gatech.edu), Johannes E. Leisen (johannes.leisen@chemistry.gatech.edu), and Andrew C. McShan (andrew.mcshan@chemistry.gatech.edu)

Extended Analysis:

The supplementary extended analysis section describes specific examples of how 1D ^1H NMR data of wine can be analyzed, interpreted, and linked to wine properties.

Examples of data interpretation

To promote active engagement, participants choose a wine of interest to visualize the NMR spectra and MagMet-W results of. The white wine Sauvignon Blanc, considered to have one of the "best" aromas and common at local restaurants, was the most popular choice. The primary chemical components of the chosen wines were highlighted: ethanol, other alcohols, organic acids, sugars, wine faults, amino acids, polyphenols, vitamins, and nucleobases. A short PowerPoint that summarized the major findings was presented where participants asked questions relating to the results and their implications (Supplementary PowerPoint). A full extended analysis of how ^1H NMR data can be used to link the chemical composition of wine to wine characteristics and properties is presented in the supplementary information.

We described how the wine making process involves a regimen of biochemical processes driven by enzymes from a host of different microbial species, including yeast and lactic acid bacteria [1]. The resulting wine contains a mixture of biomolecules and metabolites, including alcohols (i.e., ethanol, methanol), organic acids, amino acids, sugars, nucleobases/nucleotides, vitamins, polyphenols, and wine "faults" [2–4]. Wine faults are compounds that negatively influence wine characteristics, such as taste, smell, or appearance [5]. We compared ^1H NMR spectral overlays containing MagMet-W peak annotations between similar wine types: red (Supplementary Figure 6), rosé (Supplementary Figure 7), and white (Supplementary Figure 8). Participants were surprised to observe that the spectral features of wines within the same class were strikingly similar, although differences were noted. We highlighted ethanol peaks, which were dominant in all spectra with chemical shift values at ~1.18 and ~3.65 ppm corresponding to the methyl proton triplet and methylene proton quartet, respectively.

As examples of similarities, all red wines contained comparable concentrations of the phenol trigonelline responsible for bitter and spicy flavors (91.1 ± 15.2 μM) [2,6], the alcohol 2,3-butanediol responsible for creamy/buttery flavor (5884.9 ± 846.7 μM) [7,8], and the organic acid tartaric acid that regulates wine characteristics and imparts a tart or sour taste (3947.1 ± 724.8 μM) [7,9] (Supplementary Figure 6). As a representative example of differences, the phenolic compound caftarate (i.e., caftaric acid) that serves as a monitor of oxidative stress (193 μM in Beaujolais Nouveau versus 51.8 μM in Cabernet Sauvignon) and the organic acid oxoglutarate that has a detrimental effect on wine aroma and stability (416.1 μM in Pinot Noir versus 1.6 μM in Syrah) differed across red wines [10–13] (Supplementary Figure 6). In contrast, ^{1}H NMR spectral features of wines within different classes were more distinguished from each other (Figure 3A,B). As examples of similarities, red, white, and rosé wines each contained comparable concentrations of trigonelline (79.4 ± 8.5 μM). Several compounds, such as the amino acid L-proline, the organic acid lactic acid, the organic acid ethyl lactate, the sugar fructose, and the organic acid galacturonate exhibited strikingly different concentrations between classes (Figure 3A,B).

We found that most participants had no problem understanding the most important concepts of a 1D ^{1}H NMR spectrum of wine:

(1) Each peak in the NMR spectrum represents a hydrogen atom attached to one of the molecules. Most molecules have many hydrogen atoms attached to them, which results in multiple NMR peaks for a single molecule.
(2) There are many different types of molecules in a single wine sample, so there are many different NMR peaks.
(3) Scientists can analyze the NMR peak position and peak patterns to figure out which molecules are present and then link those molecules to wine properties. Automated softwares like MagMet-W allow even non-experts to quickly process and analyze NMR data to profile wine.
(4) NMR spectra are like "fingerprints" that allow researchers to compare and contrast the features of different wines and identify wine fraud.

Using multivariate analysis to group wines by chemical composition

Multivariate analysis is a statistical approach that examines multiple variables to uncover patterns and relationships among samples based on specific features. In the context of NMR data, these features may include spectral patterns or concentrations of different compounds present in the sample. We reasoned that multivariate analysis would be an excellent educational tool to highlight how similarities and differences between NMR samples can classify different types of wines by comparing spectral features [14–16]. While we do not focus on it here, multivariate analysis of NMR data also provides opportunities for students and instructors to explore how the different algorithms function (for details on the pedagogical application of multivariate analysis to compare and contrast NMR samples see refs [17–19]).

To perform the multivariate analysis, we used MetaboAnalyst 6.0, a free, open-source software that allows users to normalize data and apply a suite of statistical analyses, including sparse partial least squares-discriminant analysis (sPLS-DA) [20]. MetaboAnalyst allows non-experts the ability to readily explore different types of multivariate analyses without the need for coding expertise. The MagMet-W determined compound concentration table for each wine sample was provided as the input to MetaboAnalyst. Full details of how to perform the analysis are outlined in the

Supplementary Tutorial file. Due to time constraints, the multivariate analysis was carried out in advance by the Ph.D. student facilitators. The results were then presented to participants as an example of how the wine samples could be clustered and classified based on NMR spectral features.

MetaboAnalyst outputs a 2D scores plot (Extended Figure 1). The X-axis (component 1, 24.8%) represents a linear combination of the 70 compound concentrations selected by the sPLS-DA algorithm to maximize discrimination between wine classes. The percentage indicates the proportion of variance in class separation explained by component 1. The Y-axis (component 2, 7%) is another weighted combination of the 70 compounds, orthogonal to component 1, that captures additional variance useful for distinguishing wine classes. Each point in the scores plot represents a wine sample that has been re-expressed in terms of component 1 and component 2, which are the new variables created by combining information from all 70 compounds. This projection into 2D space makes it easier to see how samples group together based on similarities and differences in their overall chemical profiles. The sPLS-DA 2D scores plot shows excellent grouping of red, white, and rosé wines based on the concentration of the 70 compounds annotated by MagMet-W (Extended Figure 1).

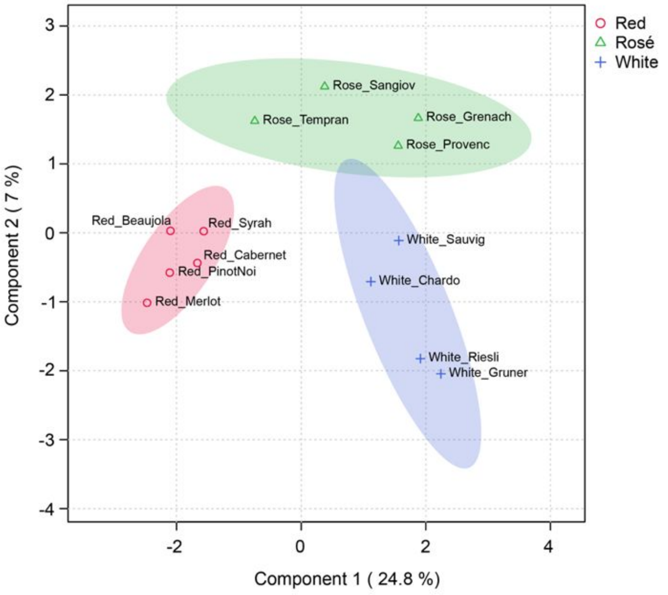

Extended Figure 1. Classification of wine type by multivariate analysis based on MagMet-W identified spectral features. An automated Sparse Partial Least Squares Discriminant Analysis (sPLS-DA) was performed using the MetaboAnalyst 6.0 webserver. The input for the analysis was the concentrations (in µM) for each of the 70 compounds determined from MagMet-W for each of the wine samples. In the scores plot, the X-axis (component 1, 24.8%) runs from –2 to 4 and shows the main direction along which the wine samples differ based on their chemical profiles. The Y-axis (component 2, 7%) runs from –4 to 3 and shows an additional, smaller direction of difference between the samples. The values (–2 to 4 on Component 1 and –4 to 3 on Component 2) are called scores. They do not correspond to raw measurements like µM concentrations. Instead, they are new coordinates for each wine sample after the sPLS-DA algorithm has transformed the original 70 compound concentrations into new axes (components). Together, these two components

summarize much of the variation in the data, making it easier to see how red, white, and rosé wines separate into groups. Wines are colored by class: red wine in red, white wine in blue, and rosé wine in green. The orange wine is not included, since only one sample was obtained for that class. The colored ellipses represent the 95% confidence levels of discrimination. The multivariate analysis was carried out as outlined in the Supplementary Tutorial file.

Connecting chemical profiling to wine terroir, grape cultivar, fermentation process, and wine characteristics

A major motivation for performing automated chemical profiling is to directly connect identified compounds to wine terroir, grape cultivar, the fermentation process, and wine characteristics [2,4,7,10,21,22]. To guide future implementations of the learning module for outreach and chemical education, examples of how MagMet-W analyzed NMR data obtained across different wines can enable discovery of these connections is described below.

Overview

MagMet-W is an automated NMR data processing and analysis workflow. It takes in raw NMR data of different wine samples, processes the spectra, and fits them. MagMet-W automatically outputs the estimated concentrations of 70 different compounds in micromolar (μM) units. These concentrations can be obtained from the downloaded CSV results file from MagMet-W. In-house Python 3 scripts were used to compare and contrast the concentration of each compound or compound class across different wine samples. The Python scripts use the MagMet-W results CSV file as input. The Python 3 script named "complete_wine_analysis_from_MagMetW.py" compares the percentage of different compound classes across different wine samples (Extended Figure 2). The Python 3 script named "bar-graph_one-compound_wine_analysis_from_MagMetW.py" compares the concentration of a specific compound across different wine samples. Full details of determining percentages of compounds in wine using the above Python 3 scripts are noted in the Supplementary Tutorial and Materials and Procedures files.

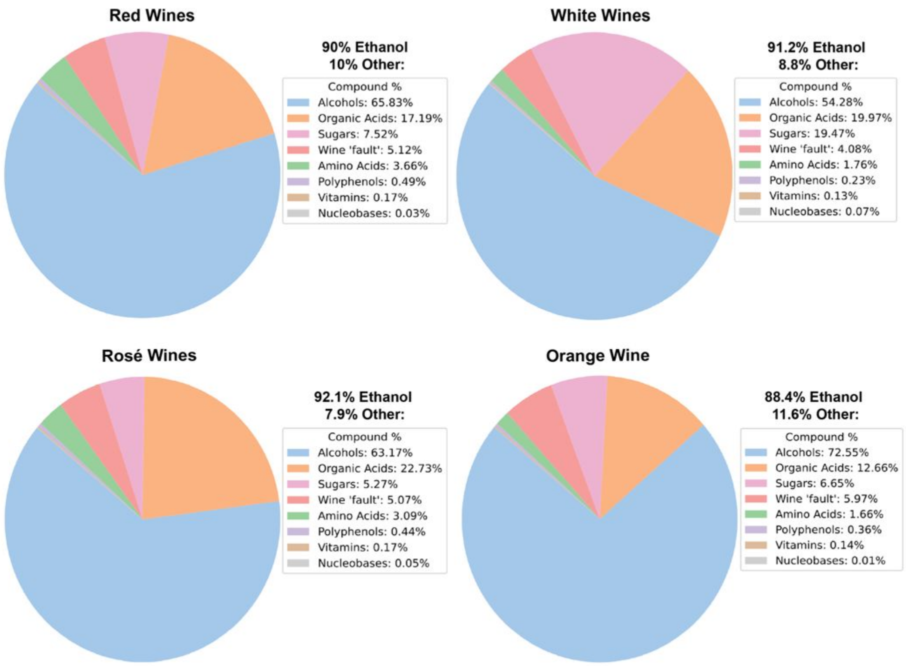

Extended Figure 2. Summary of chemical components detected in the 1D ^{1}H NMR spectra of 14 different wines (by percentage) automatically identified and quantified by MagMet-W. The average percentage of ethanol determined across red, rosé, orange, and white wines is noted to the right. The pie charts summarize of "Other" non-ethanol compounds. The pie charts highlight the average percentage of each type of compound (i.e., non-ethanol alcohols, organic acids, sugars, wine faults, amino acids, polyphenols, vitamins, and nucleobases) across different wine types. The pie charts are generated from the Python 3 script complete_wine_analysis_from_MagMetW.py.

Ethanol

Apart from water, ethanol is the primary chemical component of wine where it is generated through glycolysis and pyruvate conversion during yeast fermentation [23,24]. Ethanol is a simple organic molecule that can be easily detected in ^{1}H NMR since (i) it is one of the most abundant compounds and its signal towers over other compounds, and (ii) it shows three distinct NMR peaks: a triplet around 1.18 ppm for the methyl proton (CH3) group, a quartet around 3.65 ppm for the methylene proton (CH2) group, and a singlet for the OH proton, which is typically broadened or absent in aqueous solvent due to solvent exchange. Because ethanol's NMR spectrum is simpler than many compounds, it is a nice system to explain the concept of splitting patterns and chemical shift values [25]. Ethanol consumption influences a myriad of biological processes including cognition, energy metabolism, cellular structures, and immunological responses [26–29]. Ethanol also influences the taste and aroma of wine by eliciting taste, smell, and chemesthetic responses [29,30].

Consistent with previous reports, ethanol percentages in the wines tested here were consistently high and represented 88 to 93 percent of all MagMet-W quantified compounds [2,31] (Extended Figure 2). Ethanol concentrations in wine are the result of several factors including grape sugar content, yeast strains, and other details of the fermentation process [32]. Several of the rosé wines characterized in our demonstration were noted to have been generated in stainless-steel controlled

fermentation environments that can enhance yeast growth and metabolism, leading to more efficient conversion of sugars into ethanol [33] (Extended Figure 3). In contrast, a portion of the red wines sampled underwent open barrel fermentation, an approach more susceptible to environmental variability. In addition to allowing for ethanol evaporation, open vessels may introduce wild yeast strains and expose yeast to additional stressors, influencing ethanol and secondary metabolite production [33,34].

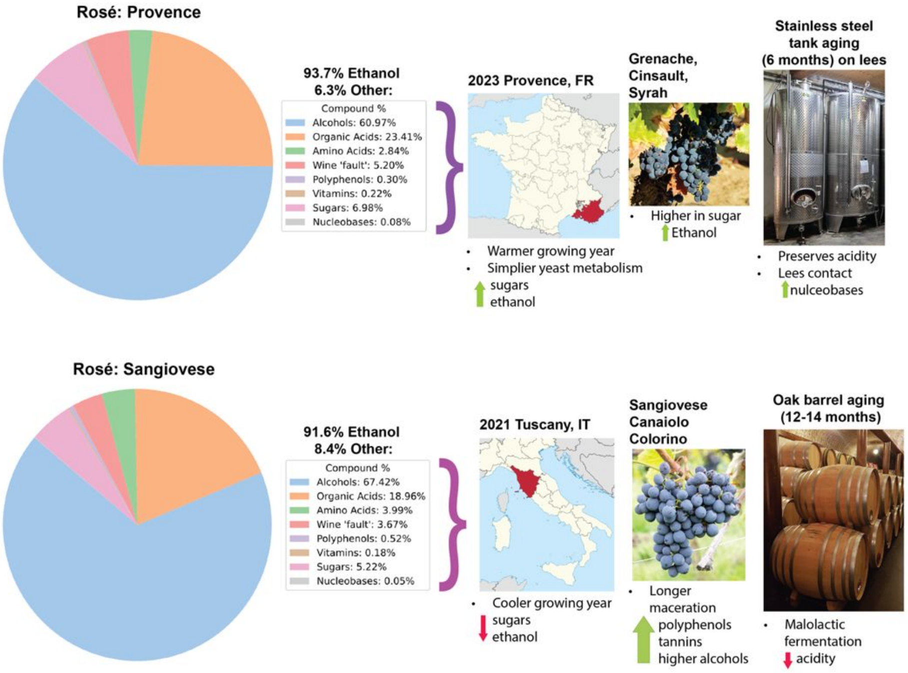

Extended Figure 3. Example of how information obtained from MagMet-W provides insights into wine terroir and characteristics. Pie chart summaries of “other” compounds for two rosé wines are shown. The pie charts highlight the average percentage of each type of compound (i.e., non-ethanol alcohols, organic acids, sugars, wine faults, amino acids, polyphenols, vitamins, and nucleobases) across different wines. The observed quantities of specific compounds (i.e., sugars, ethanol, polyphenols, nucleobases, and organic acids) can be tied to 1. the geographic region and associated climate, 2. the type of grape cultivar, and 3. conditions for wine aging. The pie charts are generated from the Python 3 script complete_wine_analysis_from_MagMetW.py.

Alcohols

Non-ethanol alcohols are also produced by yeast during the fermentation from sugars and amino acids, and they influence wine taste and aroma [35]. In our dataset, concentrations of non-ethanol alcohols were highest in red, orange, and rosé wines, but relatively low in white wines (Extended Figure 2). As a representative example, red and rosé wines trended towards higher concentrations of fusel alcohols, such as isobutanol, isoamyl alcohol, and 2-methylbutanol (Extended Figure 4).

This is consistent with red and rosé wines being fermented with grape skin maceration, which stimulates yeast amino acid metabolism (via the Ehrlich pathway) from which fusel alcohols are derived [36]. Given the limited involvement of grape-skin maceration in white wine fermentation processes, fusel alcohol byproducts would be expected to be reduced. Prior studies suggest that fusel alcohol content can positively affect wine characteristics by enhancing the perception of fruity, floral, and spicy sensory profiles [36,37].

Low concentrations of methanol are natively found in some wines [38]. Like fusel alcohols, methanol concentrations were on average observed to be highest in red and rosé wines, but lowest in white wines (Supplementary Figure 9). Methanol levels may be higher due to fermention with grape skin maceration where pectin methylesterase enzymes in the skins release methanol as a byproduct from de-esterification of pectin polysaccharides [39]. In addition, pectinase enzymes that generate methanol may be exogenously added during the fermentation processes to stabilize and filter wines [40].

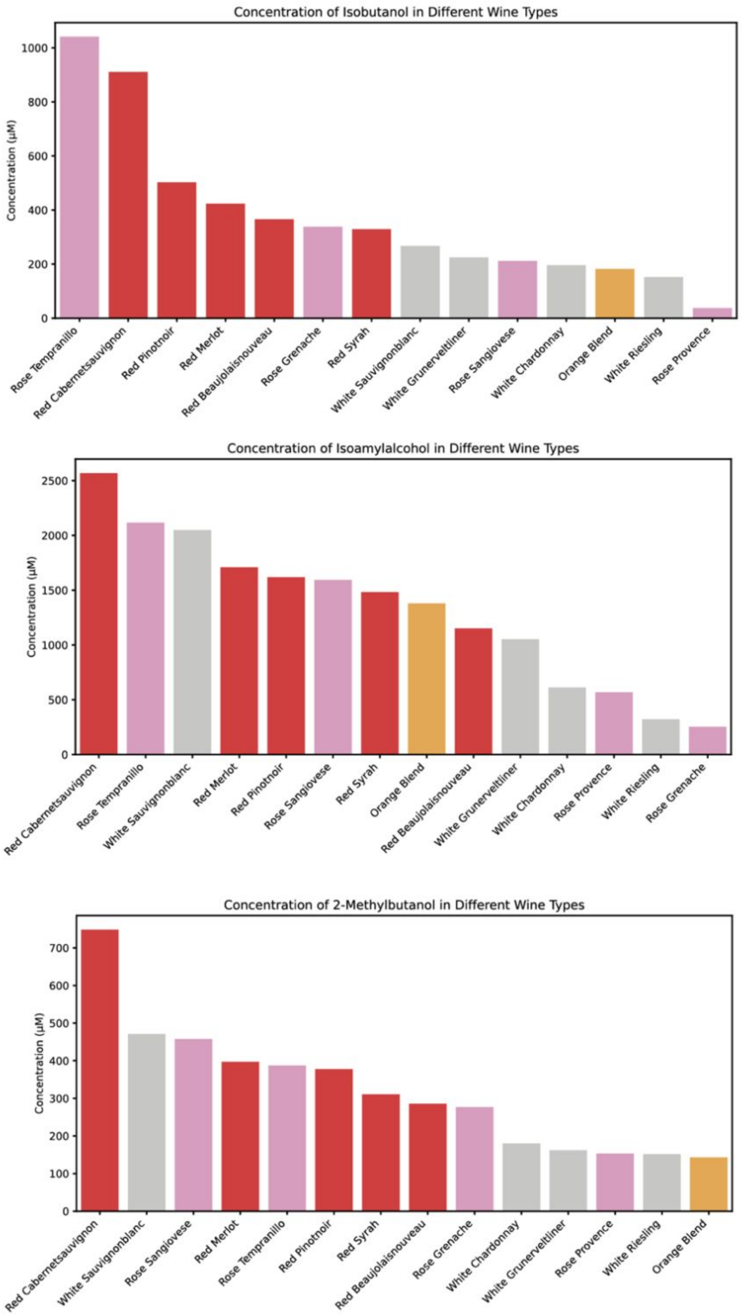

Extended Figure 4. Comparison of fusel alcohol concentrations obtained from MagMet-W across wines. The bar charts are generated from the Python 3 script bar-graph_one-compound_wine_analysis_from_MagMetW.py

Organic acids

Organic acids in wine are formed through various means, from natural grape metabolism during and after alcoholic and malolactic acid fermentation, and ultimately contribute to wine taste and

aroma [41]. Variations in organic acid profiles among different wine types result from complex differences in grape viticulture, microbial activity, and winemaking style [42]. An interesting trend reflected in our data was that red and orange wines exhibited the lowest overall acidity, while rosé wines showed the greatest acidity (Extended Figure 2). This might be attributed to differences in the application of malolactic fermentation, which is more common in red wines than rosé wines [43,44]. Malolactic fermentation is a process in which lactic acid bacteria convert dicarboxylic L-malic acid into monocarboxylic L-lactic acid [44,45]. This conversion reduces acidity and contributes to a smoother, fuller mouthfeel, which is an effect often desired in red wines [44,45]. In contrast, malolactic fermentation is typically less sought after or deliberately suppressed in white and rosé wines [46]. Consistent with this hypothesis, MagMet-W identified a general trend where malate and lactic acid were inversely correlated across red, rosé, and white wines (Extended Figure 5).

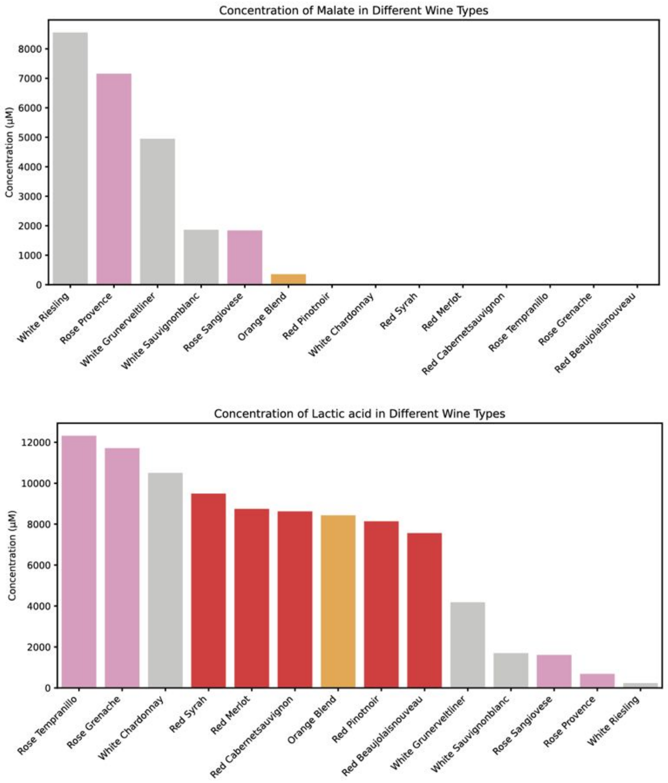

Extended Figure 5. Comparison of malate and lactic acid concentrations obtained from MagMet-W across wines. The bar charts are generated from the Python 3 script bar-graph_one-compound_wine_analysis_from_MagMetW.py

Sugars

Sugars influence wine characteristics by dictating sweetness, acidity, and alcohol content [24,47]. Glucose and fructose are the dominant sugars in most grape varieties [48]. While some grapes are naturally higher in fructose and glucose than others, both red and white grapes can be cultivated and processed by different yeast strains that produce either sweet (high sugar) or dry (low sugar) wines [49]. The final residual sugar content after fermentation is often deliberately adjusted by winemakers to achieve balance with acidity and align with desired wine characteristics. In our dataset, most wines analyzed fell on the drier end of the spectrum, although white wines displayed the highest average sugar content across all categories (Extended Figure 2). Notably, this average is skewed by the inclusion of a single Riesling sample, which exhibited an elevated sugar content attributed to high concentrations of fructose and glucose (Extended Figure 6). Previous studies have also reported high glucose and fructose ratios for Riesling wines [50,51]. Apart from grape cultivar, elevated sugars in white wines may reflect grapes harvested at advanced ripeness and fermented for a shorter duration, resulting in elevated residual sugar. In contrast, the red, rosé, and orange wines appear to have undergone more complete fermentation, consistent with drier styles and longer sugar-to-ethanol conversion periods.

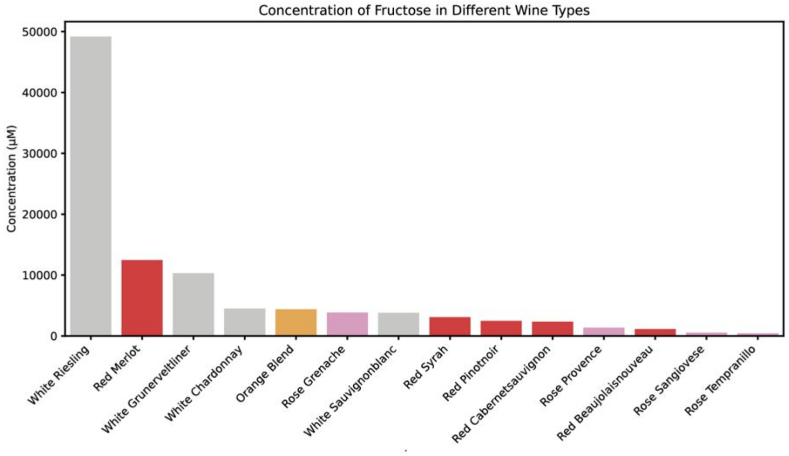

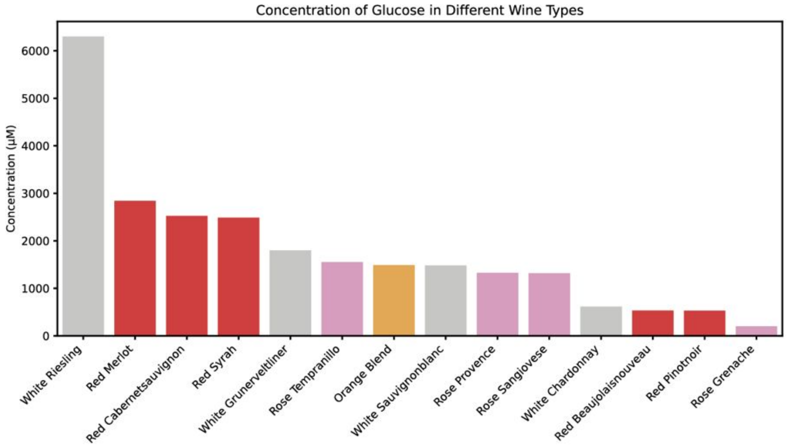

Extended Figure 6. Comparison of fructose and glucose concentrations obtained from MagMet-W across wines. The bar charts are generated from the Python 3 script bar-graph_one-compound_wine_analysis_from_MagMetW.py

Wine faults

Wine faults are undesirable compounds that typically contribute to sour taste and spoiled smell. Wine faults are often produced by microbial activity at various stages of the winemaking process [5]. For example, unhealthy or damaged grapes can attract acetic acid bacteria, while stressful fermentation conditions may lead to the formation of acetone and acetate [52]. Additionally, certain bacteria present during storage are known to produce rotting smelling biogenic amines, such as cadaverine [53]. MagMet-W contains wine faults, such as acetaldehyde, acetate, acetone, and cadaverine, whose concentration can be automatically and quantitatively determined, which allows one to link the presence of wine faults to enjoyment or dislike of different types of wines (Supplementary Figure 5).

The overall amount of wine faults was found to be 4 to 6% of the total compounds across different wine types (Extended Figure 2). Among detected faults, acetate was the most prevalent with particularly elevated levels observed in red and orange wine samples (Supplementary Figure 10). Acetate can arise as a byproduct of both yeast-driven alcoholic fermentation and malolactic fermentation, as well as through oxidative pathways involving bacteria or the oxidation of acetaldehyde [44]. While low concentrations of acetate are considered acceptable, excessive levels result in unpleasant vinegary aromas and off-flavors [54]. The elevated acetate concentrations in the orange and red wine categories are consistent with their production methods, which often include extended skin contact, prolonged fermentation, malolactic fermentation, and barrel aging. These conditions collectively provide more opportunities for microbial activity and oxygen exposure, both of which can contribute to acetate accumulation.

Another example of a wine fault detected by MagMet-W is the biogenic amine cadaverine, which is produced by lactic acid bacteria and is associated with poor sanitary practices [55]. High concentrations of cadaverine present in wines can lead to a rotten aroma similar to decaying meat or fish. We found that one of the least favorite wines of participants during the smell test (the Grüner Veltliner white wine) contained the highest concentration of cadaverine (Supplementary Figure 10). However, the presence of a single wine fault at high concentration may or may not negatively influence the aroma or taste of wines. This is complicated by all the different types of sensations, both positive, neutral, or negative, induced from a complex mixture of chemicals [5].

Amino acids

Amino acids, the building blocks of proteins and key metabolic intermediates, can influence the taste, mouthfeel and flavor of wine [56]. Their concentration and composition in wine are influenced by viticulture, maceration, yeast metabolism, and the use of yeast nutrients [57]. L-proline is a dominant amino acid in freshly crushed fruit juice and remains largely unconsumed during fermentation because under standard conditions most yeast strains do not consume proline as a primary nitrogen source [58]. However, under certain conditions, the addition of yeast nutrients can

enhance proline metabolism, potentially reducing its final concentration in wine [57]. In contrast, maceration increases L-proline [59]. Differences in grape climate conditions, soil composition, water availability, and foliar fertilization could be at play since L-proline is a known osmoprotectant and accumulates during stress [60] (Extended Figure 7A). MagMet-W identified that red wines undergoing the longest maceration period had the highest amino acid content. Notably, the red Cabernet Sauvignon wine had a significantly elevated L-proline content, aligning with the vendor's vintage description of "a year of peculiar weather where our commitment to organic viticulture was vital to the successful growth of quality, balanced fruit" (Extended Figure 7B,C). This was in stark contrast to the lack of L-proline for the white Riesling wine (Extended Figure 7B,C).

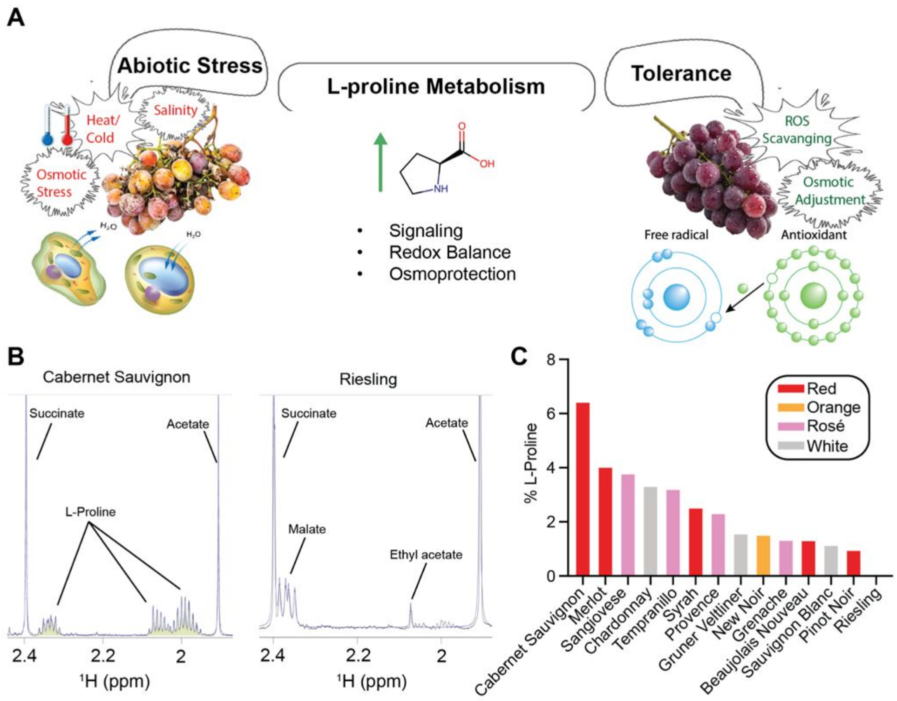

Extended Figure 7. MagMet-W identifies L-proline accumulation in response to grape stress. (A) Cartoon schematic of how grape stress leads to L-proline accumulation. Environmentally driven abiotic stress (i.e., osmotic changes, heat/cold changes, salinity changes) results in upregulation of L-proline metabolism. L-proline helps the grape cope with stress through reactive oxygen species (ROS) scavenging and maintenance of osmotic balance. (B) Screenshots from the MagMet-W results windows in the L-proline region (chemical shift range 1.9 to 2.4 ppm) for Cabernet Sauvignon (left) and Riesling (right). The JSpectraViewer shows the Fourier transformed/processed "fid" (black) versus the MagMet-W fitted spectra (blue). MagMet-W identified NMR peaks are labeled with the L-proline NMR peaks highlighted in yellow. (C) Quantification of the percentage of L-proline present in each wine obtained from MagMet-W. The percentage of L-proline is calculated relative to all wine components except for ethanol. Wines are colored by wine-type in the bar graph. The bar charts are generated from the Python 3 script bar-graph_one-compound_wine_analysis_from_MagMetW.py

Polyphenols

Polyphenols are a diverse class of plant secondary metabolites characterized by the presence of one or more hydroxyl (–OH) groups attached to aromatic hydrocarbon rings [61]. The polyphenols detected in wine are naturally occurring compounds found in the skin, seeds, and pulp of grapes, where they contribute to wine's color, aroma, flavor, and antioxidant properties [62]. Polyphenols are known for their antioxidant and anti-inflammatory properties, which contribute to a range of health benefits including improved cardiovascular health, anti-cancer effects, support for gut microbiota health, and diabetes prevention [63]. Red grape varieties are inherently richer in polyphenols, and prolonged skin contact during maceration further increases their extraction into the wine matrix [62]. MagMet-W analysis of our wine dataset aligns with those findings: polyphenol concentrations were highest in red wines, which undergo extended maceration of red grape skins (Extended Figure 2). Rosé wines showed moderately high levels, reflecting their shorter maceration period. Orange wines, produced from white grapes with extended skin contact, followed, while white wines, typically fermented with minimal or no skin contact, had the lowest polyphenol content. Among the polyphenols identified, tyrosol was the most abundant across the red, orange, and white wine categories (Extended Figure 8). Tyrosol is a non-flavonoid antioxidant present in both red and white grapes and contributes to oxidative stability and wine aroma [62]. Interestingly, rosé wines displayed slightly elevated levels of caftarate, a compound known to be particularly abundant in Grenache grapes (Extended Figure 8) [64,65]. Caftarate has been reported to undergo enzymatic oxidation, forming pigment complexes with anthocyanins and potentially enhancing color stability in rosé wines [66].

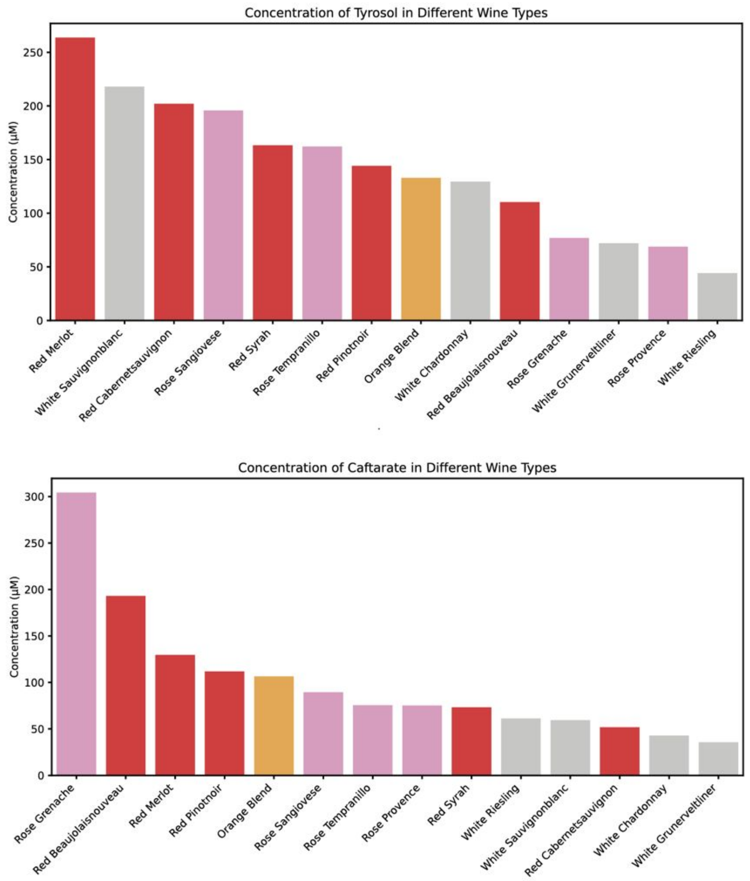

Extended Figure 8. Comparison of tyrosol and caftarate concentrations obtained from MagMet-W across wines. The bar charts are generated from the Python 3 script bar-graph_one-compound_wine_analysis_from_MagMetW.py

Extended References

(1) Moreno-Arribas, M. V.; Polo, M. C. Winemaking Biochemistry and Microbiology: Current Knowledge and Future Trends. *Crit. Rev. Food Sci. Nutr.* **2005**, *45* (4), 265–286. https://doi.org/10.1080/10408690490478118.

(2) Bl, L.; M, R.; Y, D.; M, L.; M, B.; F, S.; D, B.; A, S.; R, M.; Ds, W. Automatic Chemical Profiling of Wine by Proton Nuclear Magnetic Resonance Spectroscopy. *ACS Food Sci. Technol.* **2024**, *4* (8). https://doi.org/10.1021/acsfoodscitech.4c00298.

(3) Gerginova, D.; Simova, S. Chemical Profiling of Wines Produced in Bulgaria and Distinction from International Grape Varieties. *ACS Omega* **2023**, *8* (21), 18702–18713. https://doi.org/10.1021/acsomega.3c00636.

(4) Mao, I. L.; Costa, G. D.; Richard, T. ^{1}H-NMR Metabolomics for Wine Screening and Analysis. *OENO One* **2023**, *57* (1), 15–31. https://doi.org/10.20870/oeno-one.2023.57.1.7134.

(5) Ailer, Š.; Jakabová, S.; Benešová, L.; Ivanova-Petropulos, V. Wine Faults: State of Knowledge in Reductive Aromas, Oxidation and Atypical Aging, Prevention, and Correction Methods. *Molecules* **2022**, *27* (11), 3535. https://doi.org/10.3390/molecules27113535.

(6) Gerginova, D.; Simova, S. Chemical Profiling of Wines Produced in Bulgaria and Distinction from International Grape Varieties. *ACS Omega* **2023**, *8* (21), 18702–18713. https://doi.org/10.1021/acsomega.3c00636.
(7) Le Mao, I.; Martin-Pernier, J.; Bautista, C.; Lacampagne, S.; Richard, T.; Da Costa, G. 1H-NMR Metabolomics as a Tool for Winemaking Monitoring. *Molecules* **2021**, *26* (22), 6771. https://doi.org/10.3390/molecules26226771.
(8) Bartowsky, E. J.; Henschke, P. A. The "buttery" Attribute of Wine--Diacetyl--Desirability, Spoilage and Beyond. *Int. J. Food Microbiol.* **2004**, *96* (3), 235–252. https://doi.org/10.1016/j.ijfoodmicro.2004.05.013.
(9) Cui, W.; Wang, X.; Han, S.; Guo, W.; Meng, N.; Li, J.; Sun, B.; Zhang, X. Research Progress of Tartaric Acid Stabilization on Wine Characteristics. *Food Chem. X* **2024**, *23*, 101728. https://doi.org/10.1016/j.fochx.2024.101728.
(10) Ali, K.; Maltese, F.; Toepfer, R.; Choi, Y. H.; Verpoorte, R. Metabolic Characterization of Palatinate German White Wines According to Sensory Attributes, Varieties, and Vintages Using NMR Spectroscopy and Multivariate Data Analyses. *J. Biomol. NMR* **2011**, *49* (3–4), 255–266. https://doi.org/10.1007/s10858-011-9487-3.
(11) Hayasaka, Y.; Black, C. A.; Hack, J.; Smith, P. Structural Characterization of Reaction Products of Caftaric Acid and Bisulfite Present in a Commercial Wine Using High Resolution Mass Spectrometric and Nuclear Magnetic Resonance Techniques. *Food Chem.* **2017**, *230*, 99–107. https://doi.org/10.1016/j.foodchem.2017.03.005.
(12) El Rayess, Y.; Nehme, N.; Azzi-Achkouty, S.; Julien, S. G. Wine Phenolic Compounds: Chemistry, Functionality and Health Benefits. *Antioxidants* **2024**, *13* (11), 1312. https://doi.org/10.3390/antiox13111312.
(13) Herzan, J.; Prokes, K.; Baron, M.; Kumsta, M.; Pavlousek, P.; Sochor, J. Study of Carbonyl Compounds in White Wine Production. *Food Sci. Nutr.* **2020**, *8* (11), 5850–5859. https://doi.org/10.1002/fsn3.1855.
(14) Lê Cao, K.-A.; Boitard, S.; Besse, P. Sparse PLS Discriminant Analysis: Biologically Relevant Feature Selection and Graphical Displays for Multiclass Problems. *BMC Bioinformatics* **2011**, *12* (1), 253. https://doi.org/10.1186/1471-2105-12-253.
(15) Hu, B.; Cao, Y.; Zhu, J.; Xu, W.; Wu, W. Analysis of Metabolites in Chardonnay Dry White Wine with Various Inactive Yeasts by 1H NMR Spectroscopy Combined with Pattern Recognition Analysis. *AMB Express* **2019**, *9* (1), 140. https://doi.org/10.1186/s13568-019-0861-y.
(16) Herbert-Pucheta, J. E.; Lozada-Ramírez, J. D.; Ortega-Regules, A. E.; Hernández, L. R.; Anaya de Parrodi, C. Nuclear Magnetic Resonance Metabolomics with Double Pulsed-Field-Gradient Echo and Automatized Solvent Suppression Spectroscopy for Multivariate Data Matrix Applied in Novel Wine and Juice Discriminant Analysis. *Mol. Basel Switz.* **2021**, *26* (14), 4146. https://doi.org/10.3390/molecules26144146.
(17) Anderson, S. L.; Rovnyak, D.; Strein, T. G. Identification of Edible Oils by Principal Component Analysis of 1H NMR Spectra. *J. Chem. Educ.* **2017**, *94* (9), 1377–1382. https://doi.org/10.1021/acs.jchemed.7b00012.
(18) Sandusky, P. O. Introducing Undergraduate Students to Metabolomics Using a NMR-Based Analysis of Coffee Beans. *J. Chem. Educ.* **2017**, *94* (9), 1324–1328. https://doi.org/10.1021/acs.jchemed.6b00559.
(19) Gauthier, J. R.; Burns, D.; Sheng, J.; D'eon, J. C. Exploring the Composition and Authenticity of Honey and Syrup Samples Using Quantitative NMR Spectroscopy and

Principal Component Analysis in an Upper-Year Undergraduate Analytical Environmental Course. *J. Chem. Educ.* **2023**, *100* (1), 161–169. https://doi.org/10.1021/acs.jchemed.2c00501.
(20) Pang, Z.; Lu, Y.; Zhou, G.; Hui, F.; Xu, L.; Viau, C.; Spigelman, A. F.; MacDonald, P. E.; Wishart, D. S.; Li, S.; Xia, J. MetaboAnalyst 6.0: Towards a Unified Platform for Metabolomics Data Processing, Analysis and Interpretation. *Nucleic Acids Res.* **2024**, *52* (W1), W398–W406. https://doi.org/10.1093/nar/gkae253.
(21) Denchai, S.; Sasomsin, S.; Prakitchaiwattana, C.; Phuenpong, T.; Homyog, K.; Mekboonsonglarp, W.; Settachaimongkon, S. Influence of Different Types, Utilization Times, and Volumes of Aging Barrels on the Metabolite Profile of Red Wine Revealed by 1H-NMR Metabolomics Approach. *Molecules* **2023**, *28* (18), 6716. https://doi.org/10.3390/molecules28186716.
(22) Viskić, M.; Bandić, L. M.; Korenika, A.-M. J.; Jeromel, A. NMR in the Service of Wine Differentiation. *Foods* **2021**, *10* (1), 120. https://doi.org/10.3390/foods10010120.
(23) Martinez, P.; Kerr, W. C.; Subbaraman, M. S.; Roberts, S. C. M. New Estimates of the Mean Ethanol Content of Beer, Wine, and Spirits Sold in the U.S. Show a Greater Increase in per Capita Alcohol Consumption than Previous Estimates. *Alcohol. Clin. Exp. Res.* **2019**, *43* (3), 509–521. https://doi.org/10.1111/acer.13958.
(24) Chambers, P. J.; Pretorius, I. S. Fermenting Knowledge: The History of Winemaking, Science and Yeast Research. *EMBO Rep.* **2010**, *11* (12), 914–920. https://doi.org/10.1038/embor.2010.179.
(25) Lawson, I. J.; Ewart, C.; Kraft, A.; Ellis, D. Demystifying NMR Spectroscopy: Applications of Benchtop Spectrometers in the Undergraduate Teaching Laboratory. *Magn. Reson. Chem. MRC* **2020**, *58* (12), 1256–1260. https://doi.org/10.1002/mrc.5055.
(26) Valenzuela, C. F. Alcohol and Neurotransmitter Interactions. *Alcohol Health Res. World* **1997**, *21* (2), 144–148.
(27) Neafsey, E. J.; Collins, M. A. Moderate Alcohol Consumption and Cognitive Risk. *Neuropsychiatr. Dis. Treat.* **2011**, *7*, 465–484. https://doi.org/10.2147/NDT.S23159.
(28) Wilson, D. F.; Matschinsky, F. M. Ethanol Metabolism: The Good, the Bad, and the Ugly. *Med. Hypotheses* **2020**, *140*, 109638. https://doi.org/10.1016/j.mehy.2020.109638.
(29) Harris, R. A.; Trudell, J. R.; Mihic, S. J. Ethanol's Molecular Targets. *Sci. Signal.* **2008**, *1* (28), re7. https://doi.org/10.1126/scisignal.128re7.
(30) Nolden, A. A.; Hayes, J. E. Perceptual Qualities of Ethanol Depend on Concentration, and Variation in These Percepts Associates with Drinking Frequency. *Chemosens. Percept.* **2015**, *8* (3), 149–157. https://doi.org/10.1007/s12078-015-9196-5.
(31) M, R.; M, L.; Bl, L.; M, B.; N, A.; Rv, F.; As, C.; Y, D.; M, J.; H, S.; V, G.; T, S.; E, O.; H, P.; R, M.; Ds, W. MagMet: A Fully Automated Web Server for Targeted Nuclear Magnetic Resonance Metabolomics of Plasma and Serum. *Magn. Reson. Chem. MRC* **2023**, *61* (12). https://doi.org/10.1002/mrc.5371.
(32) Gonzalez, R.; Guindal, A. M.; Tronchoni, J.; Morales, P. Biotechnological Approaches to Lowering the Ethanol Yield during Wine Fermentation. *Biomolecules* **2021**, *11* (11), 1569. https://doi.org/10.3390/biom11111569.
(33) Eliodório, K. P.; Cunha, G. C. de G. E.; Müller, C.; Lucaroni, A. C.; Giudici, R.; Walker, G. M.; Alves, S. L.; Basso, T. O. Advances in Yeast Alcoholic Fermentations for the Production of Bioethanol, Beer and Wine. *Adv. Appl. Microbiol.* **2019**, *109*, 61–119. https://doi.org/10.1016/bs.aambs.2019.10.002.

(34) Pfahl, L.; Catarino, S.; Fontes, N.; Graça, A.; Ricardo-da-Silva, J. Effect of Barrel-to-Barrel Variation on Color and Phenolic Composition of a Red Wine. *Foods* **2021**, *10* (7), 1669. https://doi.org/10.3390/foods10071669.
(35) de-la-Fuente-Blanco, A.; Sáenz-Navajas, M.-P.; Ferreira, V. On the Effects of Higher Alcohols on Red Wine Aroma. *Food Chem.* **2016**, *210*, 107–114. https://doi.org/10.1016/j.foodchem.2016.04.021.
(36) Hazelwood, L. A.; Daran, J.-M.; van Maris, A. J. A.; Pronk, J. T.; Dickinson, J. R. The Ehrlich Pathway for Fusel Alcohol Production: A Century of Research on Saccharomyces Cerevisiae Metabolism. *Appl. Environ. Microbiol.* **2008**, *74* (8), 2259–2266. https://doi.org/10.1128/AEM.02625-07.
(37) Ofoedu, C. E.; Ofoedu, E. O.; Chacha, J. S.; Owuamanam, C. I.; Efekalam, I. S.; Awuchi, C. G. Comparative Evaluation of Physicochemical, Antioxidant, and Sensory Properties of Red Wine as Markers of Its Quality and Authenticity. *Int. J. Food Sci.* **2022**, *2022*, 8368992. https://doi.org/10.1155/2022/8368992.
(38) Shen, J.; Huang, W.; You, Y.; Zhan, J. Controlling Strategies of Methanol Generation in Fermented Fruit Wine: Pathways, Advances, and Applications. *Compr. Rev. Food Sci. Food Saf.* **2024**, *23* (6), e70048. https://doi.org/10.1111/1541-4337.70048.
(39) Lin, J.; Massonnet, M.; Cantu, D. The Genetic Basis of Grape and Wine Aroma. *Hortic. Res.* **2019**, *6*, 81. https://doi.org/10.1038/s41438-019-0163-1.
(40) Espejo, F. Role of Commercial Enzymes in Wine Production: A Critical Review of Recent Research. *J. Food Sci. Technol.* **2021**, *58* (1), 9–21. https://doi.org/10.1007/s13197-020-04489-0.
(41) Robles, A.; Fabjanowicz, M.; Chmiel, T.; Płotka-Wasylka, J. Determination and Identification of Organic Acids in Wine Samples. Problems and Challenges. *TrAC Trends Anal. Chem.* **2019**, *120*, 115630. https://doi.org/10.1016/j.trac.2019.115630.
(42) Chidi, B. S.; Bauer, F. F.; Rossouw, D. Organic Acid Metabolism and the Impact of Fermentation Practices on Wine Acidity - A Review. *South Afr. J. Enol. Vitic.* **2018**, *39* (2), 315–329. https://doi.org/10.21548/39-2-3172.
(43) Virdis, C.; Sumby, K.; Bartowsky, E.; Jiranek, V. Lactic Acid Bacteria in Wine: Technological Advances and Evaluation of Their Functional Role. *Front. Microbiol.* **2021**, *11*, 612118. https://doi.org/10.3389/fmicb.2020.612118.
(44) Fu, J.; Wang, L.; Sun, J.; Ju, N.; Jin, G. Malolactic Fermentation: New Approaches to Old Problems. *Microorganisms* **2022**, *10* (12), 2363. https://doi.org/10.3390/microorganisms10122363.
(45) Mendes Ferreira, A.; Mendes-Faia, A. The Role of Yeasts and Lactic Acid Bacteria on the Metabolism of Organic Acids during Winemaking. *Foods* **2020**, *9* (9), 1231. https://doi.org/10.3390/foods9091231.
(46) Dimopoulou, M.; Troianou, V.; Paramithiotis, S.; Proksenia, N.; Kotseridis, Y. Evaluation of Malolactic Starters in White and Rosé Winemaking of Moschofilero Wines. *Appl. Sci.* **2022**, *12* (11), 5722. https://doi.org/10.3390/app12115722.
(47) Reboredo-Rodríguez, P.; González-Barreiro, C.; Rial-Otero, R.; Cancho-Grande, B.; Simal-Gándara, J. Effects of Sugar Concentration Processes in Grapes and Wine Aging on Aroma Compounds of Sweet Wines—a Review. *Crit. Rev. Food Sci. Nutr.* **2015**, *55* (8), 1053–1073. https://doi.org/10.1080/10408398.2012.680524.
(48) Zhong, H.; Yadav, V.; Wen, Z.; Zhou, X.; Wang, M.; Han, S.; Pan, M.; Zhang, C.; Zhang, F.; Wu, X. Comprehensive Metabolomics-Based Analysis of Sugar Composition and Content

in Berries of 18 Grape Varieties. *Front. Plant Sci.* **2023**, *14*, 1200071. https://doi.org/10.3389/fpls.2023.1200071.

(49) Berthels, N. J.; Cordero Otero, R. R.; Bauer, F. F.; Thevelein, J. M.; Pretorius, I. S. Discrepancy in Glucose and Fructose Utilisation during Fermentation by Saccharomyces Cerevisiae Wine Yeast Strains. *FEMS Yeast Res.* **2004**, *4* (7), 683–689. https://doi.org/10.1016/j.femsyr.2004.02.005.

(50) Kliewer, W. M. The Glucose-Fructose Ratio of Vitis Vinifera Grapes. *Am. J. Enol. Vitic.* **1967**, *18* (1), 33–41. https://doi.org/10.5344/ajev.1967.18.1.33.

(51) Cornehl, L.; Gauweiler, P.; Zheng, X.; Krause, J.; Schwander, F.; Töpfer, R.; Gruna, R.; Kicherer, A. Non-Destructive Quantification of Key Quality Characteristics in Individual Grapevine Berries Using near-Infrared Spectroscopy. *Front. Plant Sci.* **2024**, *15*, 1386951. https://doi.org/10.3389/fpls.2024.1386951.

(52) Bartowsky, E. J.; Xia, D.; Gibson, R. L.; Fleet, G. H.; Henschke, P. A. Spoilage of Bottled Red Wine by Acetic Acid Bacteria. *Lett. Appl. Microbiol.* **2003**, *36* (5), 307–314. https://doi.org/10.1046/j.1472-765x.2003.01314.x.

(53) Toit, M. du; Pretorius, I. S. Microbial Spoilage and Preservation of Wine: Using Weapons from Nature's Own Arsenal -A Review. *South Afr. J. Enol. Vitic.* **2000**, *21* (1), 74–96. https://doi.org/10.21548/21-1-3559.

(54) Vilela-Moura, A.; Schuller, D.; Mendes-Faia, A.; Silva, R. D.; Chaves, S. R.; Sousa, M. J.; Côrte-Real, M. The Impact of Acetate Metabolism on Yeast Fermentative Performance and Wine Quality: Reduction of Volatile Acidity of Grape Musts and Wines. *Appl. Microbiol. Biotechnol.* **2011**, *89* (2), 271–280. https://doi.org/10.1007/s00253-010-2898-3.

(55) Costantini, A.; Vaudano, E.; Pulcini, L.; Carafa, T.; Garcia-Moruno, E. An Overview on Biogenic Amines in Wine. *Beverages* **2019**, *5* (1), 19. https://doi.org/10.3390/beverages5010019.

(56) Espinase Nandorfy, D.; Watson, F.; Likos, D.; Siebert, T.; Bindon, K.; Kassara, S.; Shellie, R.; Keast, R.; Francis, I. l. Influence of Amino Acids, and Their Interaction with Volatiles and Polyphenols, on the Sensory Properties of Red Wine. *Aust. J. Grape Wine Res.* **2022**, *28* (4), 621–637. https://doi.org/10.1111/ajgw.12564.

(57) Marks, V. D.; van der Merwe, G. K.; van Vuuren, H. J. J. Transcriptional Profiling of Wine Yeast in Fermenting Grape Juice: Regulatory Effect of Diammonium Phosphate. *FEMS Yeast Res.* **2003**, *3* (3), 269–287. https://doi.org/10.1016/S1567-1356(02)00201-5.

(58) Huang, H. L.; Brandriss, M. C. The Regulator of the Yeast Proline Utilization Pathway Is Differentially Phosphorylated in Response to the Quality of the Nitrogen Source. *Mol. Cell. Biol.* **2000**, *20* (3), 892–899.

(59) Stines, A. p.; Grubb, J.; Gockowiak, H.; Henschke, P. a.; Høj, P. b.; van HEESWIJCK, R. Proline and Arginine Accumulation in Developing Berries of Vitis Vinifera L. in Australian Vineyards: Influence of Vine Cultivar, Berry Maturity and Tissue Type. *Aust. J. Grape Wine Res.* **2000**, *6* (2), 150–158. https://doi.org/10.1111/j.1755-0238.2000.tb00174.x.

(60) Meena, M.; Divyanshu, K.; Kumar, S.; Swapnil, P.; Zehra, A.; Shukla, V.; Yadav, M.; Upadhyay, R. S. Regulation of L-Proline Biosynthesis, Signal Transduction, Transport, Accumulation and Its Vital Role in Plants during Variable Environmental Conditions. *Heliyon* **2019**, *5* (12), e02952. https://doi.org/10.1016/j.heliyon.2019.e02952.

(61) Zagoskina, N. V.; Zubova, M. Y.; Nechaeva, T. L.; Kazantseva, V. V.; Goncharuk, E. A.; Katanskaya, V. M.; Baranova, E. N.; Aksenova, M. A. Polyphenols in Plants: Structure,

Biosynthesis, Abiotic Stress Regulation, and Practical Applications (Review). *Int. J. Mol. Sci.* **2023**, *24* (18), 13874. https://doi.org/10.3390/ijms241813874.
(62) Gutiérrez-Escobar, R.; Aliaño-González, M. J.; Cantos-Villar, E. Wine Polyphenol Content and Its Influence on Wine Quality and Properties: A Review. *Molecules* **2021**, *26* (3), 718. https://doi.org/10.3390/molecules26030718.
(63) Buljeta, I.; Pichler, A.; Šimunović, J.; Kopjar, M. Beneficial Effects of Red Wine Polyphenols on Human Health: Comprehensive Review. *Curr. Issues Mol. Biol.* **2023**, *45* (2), 782–798. https://doi.org/10.3390/cimb45020052.
(64) Leborgne, C.; Lambert, M.; Ducasse, M.-A.; Meudec, E.; Verbaere, A.; Sommerer, N.; Boulet, J.-C.; Masson, G.; Mouret, J.-R.; Cheynier, V. Elucidating the Color of Rosé Wines Using Polyphenol-Targeted Metabolomics. *Molecules* **2022**, *27* (4), 1359. https://doi.org/10.3390/molecules27041359.
(65) Leborgne, C.; Meudec, E.; Sommerer, N.; Masson, G.; Mouret, J.-R.; Cheynier, V. Untargeted Metabolomics Approach Using UHPLC-HRMS to Unravel the Impact of Fermentation on Color and Phenolic Composition of Rosé Wines. *Molecules* **2023**, *28* (15), 5748. https://doi.org/10.3390/molecules28155748.
(66) Leborgne, C.; Lambert, M.; Ducasse, M.-A.; Meudec, E.; Verbaere, A.; Sommerer, N.; Boulet, J.-C.; Masson, G.; Mouret, J.-R.; Cheynier, V. Elucidating the Color of Rosé Wines Using Polyphenol-Targeted Metabolomics. *Mol. Basel Switz.* **2022**, *27* (4), 1359. https://doi.org/10.3390/molecules27041359.
