# Supplementary material for: Automated Chemical Profiling of Wine by Solution NMR Spectroscopy: A Demonstration for Outreach and Education
Source: J Chem Educ. 2026 Jan 6;103(2):833–45. doi: 10.1021/acs.jchemed.5c00652 (PMC12895419; doi:10.1021/acs.jchemed.5c00652)
Supplement: Supplementary file 4 [file ed5c00652_si_007.pdf]

Supplementary Information for

Automated Chemical Profiling of Wine by Solution NMR Spectroscopy: A Demonstration for Outreach and Education

Lily Capeci[1, ‡], Ruoqing Jia[1, ‡], Mary E. Peek[1], Miriam K. Simma[1], Elizabeth A. Corbin[1], FNU Vidya[1], Hongwei Wu[1*], Johannes E. Leisen[1*], Andrew C. McShan[1*]

‡These authors contributed equally to this work.

[1]School of Chemistry and Biochemistry, Georgia Institute of Technology, Atlanta, GA 30332, USA

*Correspondence: Hongwei Wu (hongwei.wu@chemistry.gatech.edu), Johannes E. Leisen (johannes.leisen@chemistry.gatech.edu), and Andrew C. McShan (andrew.mcshan@chemistry.gatech.edu)

Materials and Procedures:

The supplementary file contains additional information on materials and procedures used in the demonstration.

Materials

Sterile nuclease free grade water (from a Milli-Q purification system or Fisher Scientific #BP248450). NaH_2PO_4 (MilliporeSigma #S0751). Na_2HPO_4 (MilliporeSigma #S9763). Deuterium oxide (D_2O, 99.9%, Cambridge Isotope Laboratories # DLM-4-100). Deuterated 2,2-dimethyl-2-silapentane-5 sulfonate (DSS-d_6, Cambridge Isotope Laboratories #MilliporeSigma 613150). Amicon Ultra-0.5 Centrifugal Filters with 3 kDa molecular weight cut-off (MilliporeSigma #UFC5003). 2-Chloropyrimidine-5-carboxylic acid (CPCA, Thermo Scientific #429470050). 5 mm NMR tubes (Norelle #NOR509UP7). Lead (II) acetate (MilliporeSigma #467863). Diethylene glycol (MilliporeSigma #93171). Methanol (Fisher Scientific #A412). 0.22 µm PES filters (Fisher Scientific #13-100-106). 20 mL syringes (Fisher Scientific #14-829-21B). 15 mL falcon tubes (Fisher Scientific #14-959-53A). 1.5 mL microcentrifuge tubes (Fisher Scientific #05-408-129).

Wines

Red wines

- **Cabernet Sauvignon**: 2021 Vasse Felix Filius Cabernet Sauvignon, Margaret River, Australia

- **Merlot:** 2021 Venica & Venica Merlot, Collio, Friuli-Venezia Giulia, Italy

- **Pinot noir:** 2023 Illahe Vineyards Pinot Noir, Willamette Valley, Oregon, USA

- **Syrah:** 2023 Domaine Julien Cecillon "Les Graviers" Syrah, Rhône Valley, France

- **Beaujolais Nouveau:** 2024 Georges Duboeuf Beaujolais Nouveau, Burgundy, France

White wines

- **Chardonnay:** 2023 Tyler Winery Chardonnay, Santa Barbara County, California, USA

- **Sauvignon blanc:** 2023 Patricia Green Sauvignon Blanc, Willamette Valley, Oregon, USA

- **Riesling:** 2022 von Winning "Reiterpfad" Erste Lage Riesling Trocken, Pfalz, Germany

- **Grüner Veltliner:** 2022 L. Hiedler "Löss" Grüner Veltliner, Kamptal, Austria

Rosé wines

- **Grenache:** 2023 Cowhorn Vineyard & Garden Rosé, Applegate Valley, Rogue Valley, Oregon, USA

- **Sangiovese:** 2021 Rocca di Montegrossi, Vin Santo del Chianti Classico, Tuscany, Italy

- **Tempranillo:** 2020 Marqués de Murrieta Reserva, Rioja, Spain

- **Provence:** 2023 Château des Annibals Rosé "Suivez-Moi-Jeune-Homme", Coteaux Varois en Provence, Provence, France

Orange blend wine

- 2022 Maison Noir "New Noir", Oregon, USA

Each wine was purchased locally in Atlanta, Georgia, United States and stored corked at room temperature until opened.

Sample Preparation

Samples were prepared according to the Wishart lab's recommendations for the use of the MagMet software (https://www.magmet.ca/spectra_collection) [1,2]. Samples were prepared using freshly opened bottles of wine. First, filtering of samples was performed to remove particulates. Amicon Ultra-0.5 Centrifugal Filters were pre-rinsed seven times with sterile Milli-Q water to remove residual glycerol. Then, 500 µL of each wine was separately filtered through different 3 kDa MWCO Amicon Ultra-0.5 centrifugal filters at 10,000 RPM. Then in a 1.5 mL microcentrifuge tube, 400 µL of the wine filtrate was mixed with 100 µL of 5× NMR buffer (750 mM sodium phosphate buffer pH 7.0, 5 mM DSS-d_6, 5 mM CPCA, 50% v/v D_2O). The final samples were briefly centrifuged at 10,000 RPM for 5 minutes. Then, the supernatant was loaded into 5 mm NMR tubes. For "adulterated wines" the following were added: 80 mg/L lead (II) acetate, 10% vol/vol diethylene glycol, or 10% vol/vol methanol. Full details of the sample preparation are noted in the Supplementary Tutorial file.

NMR Spectroscopy

NMR spectra were acquired according to the Wishart lab's recommendations for the use of the

MagMet software (http://magmet.ca/spectra_collection). Briefly, 1D ^1H NMR spectra were acquired using a Bruker AVIII-HD 700 MHz spectrometer equipped with a 5 mm TCI cryoprobe. Samples were stored briefly in a Bruker SampleCase autosampler at 25°C before insertion into the spectrometer. Data were acquired in TopSpin v3.5pl5. All 1D ^1H NMR spectra were obtained using a 1D ^1H-NOESY pulse sequence (noesypr1d) with a 2 sec recovery delay (d1) with low-power pre-saturation for water suppression, a 50 msec mixing time, and a 4 sec acquisition time. The transmitter frequency was set to 4.7 ppm, and the sweep width was set to 12 ppm. A total of 8 dummy scans were used, after which 128 scans were collected. The same parameters were used for all acquisitions. All experiments were performed at 25° C. Full details of the data collection are noted in the Supplementary Tutorial file.

Automated Chemical Profiling with MagMet-W and MetaboAnalyst

MagMet-W was accessed via the freely available webserver (http://magmet.ca) [1,2]. Each wine was broken up into separate Bruker folders containing the raw NMR data ("fid") and acquisition parameters ("acqus") files. The folders were compressed into ZIP files and uploaded separately to the MagMet server. The Biofluid was set to "Wine (700 only)". NMR Frequency was set to "700 MHz". The Chemical Shift (CS) Reference was set to "DSS". CS Concentration was set to 909.09 µM. Speed was set to Standard (~7 min).

MagMet-W is an automated NMR data analysis workflow. It takes in raw NMR spectra from wine samples, processes, and fits them. MagMet-W automatically outputs the absolute concentrations of ~70 compounds reported in micromolar (µM) units. The MagMet-W results were downloaded and processed using in-house Python 3 scripts as described below. All raw NMR data and processing scripts are provided – see Data Availability statement. Multivariate analysis was performed in MetaboAnalyst 6.0 with a sparse PLS-DA algorithm [3]. Full details of running MagMet-W and MetaboAnalyst 6.0 are noted in the Supplementary Tutorial file.

Quantifying percentages and concentrations of compounds in wine

The MagMet-W analysis described above automatically quantifies the concentration of a total of 70 different compounds for each wine sample. This results in a concentration in µM for each compound present in each wine sample. These concentrations can be obtained from the downloaded CSV results file from MagMet-W. In-house Python 3 scripts were used to compare and contrast the concentration of each compound or compound class across different wine samples using the MagMet-W results file as input.

The Python 3 script named "complete_wine_analysis_from_MagMetW.py" compares the percentage of different compound classes across different wine samples. This script analyzes metabolite composition data from the MagMet-W result CSV file for each wine and generates both pie chart visualizations and summary tables. It works by first mapping each metabolite to a defined compound class (i.e., ethanol, alcohols, organic acids, amino acids, etc). Then, it reads in concentration data to determine the percentage of ethanol versus all non-ethanol compounds. Next, the script further breaks down the non-ethanol compounds into percentages of each functional class relative to the total of non-ethanol compounds. The script outputs two pie charts per CSV file: one comparing the percentage of ethanol vs. all other compounds, and another showing the percentage distributions across non-ethanol compounds. Finally, the script aggregates all results into a comparison table (with compounds assigned to their classes) and saves this summary to an Excel file for cross-sample comparison.

The Python 3 script named “bar-graph_one-compound_wine_analysis_from_MagMetW.py” compares the concentration of a specific compound across different wine samples. This script extracts the concentration of a user-specified compound of interest (i.e., Acetate) across multiple MagMet-W result CSV files. It reads each CSV file, searches for the chosen compound, and records its concentration along with the wine’s category (red, white, rosé, orange, or unknown, determined from the filename). The script then assigns each wine category a custom color, and compiles the results into a sorted table. Finally, it generates a bar plot showing the compound’s concentration across wines, colored by wine type, and saves the figure as a PDF.

Full details of determining percentages of compounds in wine using the above Python 3 scripts are noted in the Supplementary Tutorial file.

Learning Assessments

Two summative assessments were developed (one for K-12 students and one for adults) with the specific outreach goals and outcomes in mind (Supplementary Learning Assessments). The assessments were implemented on pen and paper or online evaluations via QR-codes to increase response rates.

For the K-12 participants, two multiple choice questions were given: “How was the NMR instrument used to study wine today?” and “Which of the following chemicals can be found in many wines?”. Each question included multiple options, some of which were correct and others incorrect. For each question, we binned the responses into three categories: all correct, a mix of incorrect / correct answers, or all incorrect answers. We also asked K-12 participants to respond to a short answer question: “What do you think was the coolest or funnest part of the demo?”. We binned these responses into four categories: sensory, cognitive, misunderstanding (i.e., responses are clear but incorrect), and unclear response (we could not read the handwriting or gibberish).

Examples of sensory responses:
- Machine operation: seeing the NMR tube get loaded into the spectrometer
- Ferromagnetic paperclip: seeing magnetism demonstrated
- Smelling the wine
- Seeing the NMR spectrometer cutaway

Examples of cognitive responses:
- Understanding that NMR can be used to identify molecules and determine their concentrations
- Understanding the process of determining the components of wine
- Linking chemical structures to effects on wine characteristics
- The number of chemicals present in wine
- Learning about superconducting magnetics and cryogens

Examples of misunderstanding responses:
- “The entire facility is made for wine!”

For the adult participants, we asked three short response questions: (1) “How will what you learned today influence your future wine selections”, (2) “What did you learn about how NMR spectroscopy can be used to study wine?”, and (3) “List 3 chemical components of the wine you learned about from the profiling experiments”.

For the question "How will what you learned today influence your future wine selections", answers were grouped into positive or neutral impacts. No responses were deemed negative.

Examples of positive impacts:
- "I'm going to try out some of the sample wines now"
- "Now I realize why I dislike the smell of some wines but not others"
- "It made me feel good about my current wine selection since its higher in amino acids and lower in sugars"
- "I don't usually drink but the next time I see my parents with a glass of wine, I'll start spitting facts about ancient Rome and wine poisoning"
- "It was near to see that wines grown in areas with drought produced more antioxidants"

Examples of neutral impacts:
- "It won't"
- "I don't drink wine"

For the question ""What did you learn about how NMR spectroscopy can be used to study wine?", answers were grouped into highly technical, technical, key purposes, peripheral (general) response, unrelated response, and no response categories.

Examples of technical (advanced) responses:
- "How nuclear spin and relaxation results in peaks unique to specific molecules"
- "It measures the electromagnetic spin of molecules to analyze compounds present in wine"
- "NMR spectroscopy utilizes powerful magnetics to orient the spin system to match the magnet. When the atom can return to its own spin state, data through energy gets collected"

Examples of key purposes responses:
- "NMR can identify chemical composition to analyze the quality and differences, both for research and industry"
- "It can identify what chemicals are in wine"
- "It can find harmful substances that have been added. You can tell which wine is healthier"

Examples of peripheral response:
- "Wine is complicated"

For the questions, "List 3 chemical components of the wine you learned about from the profiling experiments" answers were grouped into broadly correct, general, specific, incorrect, vague, and no response categories.

Data Availability
All 1D ^{1}H NMR Bruker raw "fid" data obtained in this study, MagMet-W processed data, and Python scripts to analyze the MagMet-W data are freely available at GitHub https://github.com/mcshanlab/Capeci_et-al_WineNMR_2025_GitHub.

A YouTube video outlining basics of NMR theory, sample preparation, data acquisitions, and data processing is also freely available:
https://www.youtube.com/watch?v=9_QPgV14mbs

Materials and Procedures References

(1) M, R.; M, L.; Bl, L.; M, B.; N, A.; Rv, F.; As, C.; Y, D.; M, J.; H, S.; V, G.; T, S.; E, O.; H, P.; R, M.; Ds, W. MagMet: A Fully Automated Web Server for Targeted Nuclear Magnetic Resonance Metabolomics of Plasma and Serum. *Magn. Reson. Chem. MRC* **2023**, *61* (12). https://doi.org/10.1002/mrc.5371.

(2) Bl, L.; M, R.; Y, D.; M, L.; M, B.; F, S.; D, B.; A, S.; R, M.; Ds, W. Automatic Chemical Profiling of Wine by Proton Nuclear Magnetic Resonance Spectroscopy. *ACS Food Sci. Technol.* **2024**, *4* (8). https://doi.org/10.1021/acsfoodscitech.4c00298.

(3) Pang, Z.; Lu, Y.; Zhou, G.; Hui, F.; Xu, L.; Viau, C.; Spigelman, A. F.; MacDonald, P. E.; Wishart, D. S.; Li, S.; Xia, J. MetaboAnalyst 6.0: Towards a Unified Platform for Metabolomics Data Processing, Analysis and Interpretation. *Nucleic Acids Res.* **2024**, *52* (W1), W398–W406. https://doi.org/10.1093/nar/gkae253.
