# Supplementary material for: Automated Chemical Profiling of Wine by Solution NMR Spectroscopy: A Demonstration for Outreach and Education
Source: J Chem Educ. 2026 Jan 6;103(2):833–45. doi: 10.1021/acs.jchemed.5c00652 (PMC12895419; doi:10.1021/acs.jchemed.5c00652)
Supplement: Supplementary file 5 [file ed5c00652_si_009.pdf]

Supplementary Information for

Automated Chemical Profiling of Wine by Solution NMR Spectroscopy: A Demonstration for Outreach and Education

Lily Capeci[1, ‡], Ruoqing Jia[1, ‡], Mary E. Peek[1], Miriam K. Simma[1], Elizabeth A. Corbin[1], FNU Vidya[1], Hongwei Wu[1*], Johannes E. Leisen[1*], Andrew C. McShan[1*]

[‡]These authors contributed equally to this work.

[1]School of Chemistry and Biochemistry, Georgia Institute of Technology, Atlanta, GA 30332, USA

*Correspondence: Hongwei Wu (hongwei.wu@chemistry.gatech.edu), Johannes E. Leisen (johannes.leisen@chemistry.gatech.edu), and Andrew C. McShan (andrew.mcshan@chemistry.gatech.edu)

Simplified NMR theory:

The supplementary file contains an example script for how teach "lay" K-12 and adult participants about simplified NMR theory and the use of NMR to study complex mixtures, such as wine.

Everything around you, from the cells in your body to the air you breathe to the food you eat, is made of tiny building blocks called atoms. An atom has a center called the nucleus, which contains positively charged protons and neutral neutrons surrounded by negatively charged electrons. These subatomic particles give atoms both chemical and physical properties. When atoms join together, they form molecules with completely new properties. For example, water molecules are made up of two hydrogen atoms linked to one oxygen atom, which gives us H_2O. Molecules are far too small to see with our eyes, so researchers have developed specialized methods to figure out what atoms are present and how they behave. Studying molecules is essential because it helps scientists understand the world around us, uncover the cause of disease, design new medicines, and create many of the products we use on a day to day basis.

One amazing fact is that many atoms behave like tiny bar magnets. Magnets push or pull other magnetic objects because of a force called magnetism, which is the result of the electromagnetic force - one of the four fundamental forces in nature. By taking advantage of the magnetic properties of the central core of atoms, called the nucleus, scientists can study molecules in great detail. A nuclear magnetic resonance (NMR) spectrometer is a powerful instrument, a bit like a hospital MRI machine, that is designed to study molecules instead of people. Here, "nuclear" refers to the nucleus of an atom, not nuclear radiation, so there is no harmful radiation involved in the process. However, in some cases NMR magnets can still be dangerous since their strong magnetic field can pull in metal objects, interfere with certain medical implants, or wipe your credit card. We can demonstrate the power of magnetism with a simple demo: if you hang a paper clip on a string near

an NMR magnet, the clip will suddenly swing toward it. That's because the paper clip's metal atoms are attracted to the magnet's powerful field. Another cool fact is that modern NMR instruments use liquid helium and liquid nitrogen to cool their magnet until it becomes superconducting. This makes the magnet both extremely powerful and very stable, which is essential for detecting even the faintest signal from the molecules that we study.

Scientists love to study things when they don't know exactly what they are made of. They call these unknowns "samples". When a researcher puts a sample inside of a very strong magnet, several hundred thousand times stronger than Earth's magnetic field, the nucleus of the atoms in the sample behave like tiny magnets. The magnets line up either with or against the stronger applied magnetic field, which comes from the NMR magnet. The interaction between the atomic nuclei in the sample and the magnet in the NMR instrument allows scientists to study the sample in incredible detail.

Once the sample is placed inside of the NMR instrument, scientists send carefully tuned signals called radio waves into it. These are the same type of invisible signals also used to send music or the news to your radio. Radio waves carry energy to the atoms and make their tiny magnets wiggle and change directions relative to the NMR magnet. When the radio waves are turned off, the atoms return to their original positions. As they do, they send out faint signals that the instrument can detect. The NMR machine measures these signals and then a computer analyzes them. The computer turns the NMR signals into peaks on a graph – we call this a spectrum. Each peak in the NMR spectrum tells us about one of the many atoms present in the sample. Together, the peaks form a pattern, sort of like a fingerprint, for each chemical present. Scientists use these patterns to figure out what the sample contains, how the atoms are arranged in space, and how those molecules interact with each other.

We believe that studying the chemical composition of wine provides a unique opportunity to showcase the power of NMR. Wine is an alcoholic beverage made from fermented grape juice. Wine is enjoyed by millions of people worldwide and its flavor, smell, and texture come from the complex mix of chemicals. The exact chemical composition is shaped by many factors, including the type of grape used, the soil conditions of growth, the climate during the growth, and details of the fermentation process. More than 60% of the global population over the age of 18 consumes or has consumed wine. But don't worry if you're not 18 yet - there are non-alcoholic versions of wine for everyone to enjoy. Because wine is a multibillion-dollar industry, it also has been the target of fraud. In the 1980s, Austrian producers secretly added a toxic antifreeze ingredient to make wines taste sweeter and richer. Around the same time in Italy, methanol was added to boost their wine's alcohol content, which caused blindness and even death. Even the ancient Romans unknowingly poisoned themselves by sweetening wine with a lead-based syrup. So, studying the chemistry of wine not only helps us appreciate how it was made and why it taste and smells like it does, but it also plays a key role in detecting fraud and keeping consumers safe.

Now consider that a sample of wine that is made of many types of molecules, including water, sugars, acids, alcohols, and other compounds that determine its color, smell, and flavor. Each kind of molecule contains atoms that behave a little differently in the magnet because of their unique surroundings. When we run NMR on a wine sample, the hydrogen signals from all the molecules in the sample create a detailed map of every component of wine. By using designated software,

scientists can figure out exactly which molecules are present and also quantitatively determine how much of each is present. This helps researchers understand wine's flavor, quality, and even where it came from. Even though wine is very complicated, NMR is so sensitive that it can break wine down to the simplest pieces, revealing fingerprints of all its molecules. Each wine has a unique fingerprint in the NMR spectra since each contains a unique chemical makeup.
