# Supplementary material for: Automated Chemical Profiling of Wine by Solution NMR Spectroscopy: A Demonstration for Outreach and Education
Source: J Chem Educ. 2026 Jan 6;103(2):833–45. doi: 10.1021/acs.jchemed.5c00652 (PMC12895419; doi:10.1021/acs.jchemed.5c00652)
Supplement: Supplementary file 6 [file ed5c00652_si_011.pdf]

**Supplementary Information for**

# Automated Chemical Profiling of Wine by Solution NMR Spectroscopy: A Demonstration for Outreach and Education

Lily Capeci[1, ‡], Ruoqing Jia[1, ‡], Mary E. Peek[1], Miriam K. Simma[1], Elizabeth A. Corbin[1], FNU Vidya[1], Hongwei Wu[1*], Johannes E. Leisen[1*], Andrew C. McShan[1*]

‡These authors contributed equally to this work.

[1]School of Chemistry and Biochemistry, Georgia Institute of Technology, Atlanta, GA 30332, USA

*Correspondence: Hongwei Wu (hongwei.wu@chemistry.gatech.edu), Johannes E. Leisen (johannes.leisen@chemistry.gatech.edu), and Andrew C. McShan (andrew.mcshan@chemistry.gatech.edu)

## Tutorial:

*The supplementary tutorial section outlines a detailed tutorial on how to prepare wine samples, record $^1$H NMR spectra, run MagMet-W, and perform downstream data processing.*

## ***Sample preparation***

Prepare the 5× NMR buffer

Prepare 10 mL of 5× NMR buffer at pH 7.0 with the recipe outlined in Supplementary Table 1.

**Supplementary Table 1. 5× NMR buffer pH 7.0 (10 mL)**

| Stock Reagent | Amount Added | Final Concentration |
|---|---|---|
| $Na_2HPO_4$<br>$NaH_2PO_4$ | 634 mg $Na_2HPO_4$<br>363 mg $NaH_2PO_4$ | 750 mM |
| DSS-$d_6$ | 11.21 mg | 5 mM |
| CPCA | 7.93 mg | 5 mM |
| 99.9% $D_2O$ | 5 mL | 50% |

*Details of how to prepare the 5× NMR buffer at pH 7.0*

Measure out 2 mL Milli-Q $H_2O$ (or double distilled $H_2O$) into a 15 mL falcon tube (Fisher Scientific #14-959-53A).

Add 634 mg $Na_2HPO_4$ and 363 mg $NaH_2PO_4$. Vortex to mix and dissolve the powder.

Add 11.21 mg DSS-$d_6$, 7.93 mg CPCA, and 5 mL 99.9% $D_2O$. Vortex to mix and dissolve.

Fill up to 10 mL with Milli-Q $H_2O$ (or double distilled $H_2O$).

Check that the pH remains at 7.0; adjust pH with HCl or NaOH as needed.

Filter the solution into a fresh 15 mL falcon tube through a 0.22 µm PES filters (Fisher Scientific #13-100-106) with a 20 mL syringe (Fisher Scientific #14-829-21B).

*Note: You can use pH paper to measure the pH if needed.*

Prepare the Amicon Filters

Fill 3 kDa MWCO Amicon Ultra-0.5 centrifugal filters (one filter for each wine to be tested) with 0.5 mL of Milli-Q $H_2O$.

Spin for 5 minutes at 10,000 RPM.

Remove the filtrate (i.e., flow through).

Repeat six more times.

Filter the wines

Uncork wine bottles.

Remove 0.5 mL of wine with a micropipette.

Transfer 50 mL of wine into a 50 mL falcon tube beforehand if required.

Fill the above "pre-washed" Amicon filter with the 0.5 mL of wine.

Spin for 10 minutes at 10,000 RPM.

Transfer 0.4 mL of the wine filtrate (i.e., flow through) into a fresh 1.5 mL microcentrifuge tube (Fisher Scientific #05-408-129).

Add 0.1 mL of 5× NMR buffer pH 7.0 into the microcentrifuge tube containing the wine flow through. Gently mix the solution.

Spin the microcentrifuge tube for 5 minutes at 10,000 RPM.

Load the supernatant of the spun-down 0.5 mL wine/buffer solution mixture into a clean 5 mm NMR tube with long NMR pipettes (Norelle #NOR509UP7).

Wine adulteration

Add the following into a fresh 1.5 mL microcentrifuge tube to the desired wines to be adulterated:

- Adulteration with 10% methanol

0.4 mL of wine filtrate (from the above step)

0.1 mL of 5× NMR buffer pH 7.0

50 μL of 100% methanol

Vortex to mix and dissolve

Spin the microcentrifuge tube for 5 minutes at 10,000 RPM.

Load the supernatant of the spun-down 0.5 mL wine/buffer solution mixture into a clean 5 mm NMR tube with long NMR pipettes (Norelle #NOR509UP7).

- Adulteration with 10% (v/v) diethylene glycol

0.4 mL of wine filtrate (from the above step)

0.1 mL of 5× NMR buffer pH 7.0

50 μL of 100% diethylene glycol

Vortex to mix and dissolve

Spin the microcentrifuge tube for 5 minutes at 10,000 RPM

Load the supernatant of the spun-down 0.5 mL wine/buffer solution mixture into a clean 5 mm NMR tube with long NMR pipettes (Norelle #NOR509UP7)

- Adulteration with 80 mg/L lead (II) acetate

0.4 mL of wine filtrate (from the above step)

0.1 mL of 5× NMR buffer pH 7.0

0.04 mg lead (II) acetate {alternatively, make a stock solution of 10 mg/mL lead (II) acetate and dilute as needed}

Vortex to mix and dissolve

Spin the microcentrifuge tube for 5 minutes at 10,000 RPM

Load the supernatant of the spun-down 0.5 mL wine/buffer solution mixture into a clean 5 mm NMR tube with long NMR pipettes (Norelle #NOR509UP7)

## ***Data acquisition***

Load, lock, shim, and tune/match sample

Open the TopSpin 3.5pl5 desktop icon

Load the 5 mm NMR tube containing wine sample into the depth gauge using a sample holder/spinner. The sample should completely cover the 3/5 mm probe reference box. The sample tube should not touch the white gauge at the bottom.

Double check there is not already a sample in the spectrometer.

If your sample is placed directly under the loading position of the SampleCase, insert the sample with the TopSpin command `ij`

If your sample is placed at a position other than directly under the loading position of the SampleCase, load the sample using the TopSpin command `sx N` where N is the position in the SampleCase where the sample is located (i.e., 4).

Show the lock display window with the TopSpin command `lockdisp`

Once the sample is loaded, lock the sample with the TopSpin command `lock`

Choose H2O+D2O (i.e., 90% $H_2O$ / 10% $D_2O$) as the lock solvent
*Note: Do not choose H2O+D2O_salt (i.e., 90% H2O/10%D2O_salt). This is for very salty samples*

Wait until the lock is complete.

Double click on an old 1D experiment (or generate a new 1D dataset as described below)

Tune/match the sample with the TopSpin command `atmm`
*Note: The "atmm" command opens the AtmaControl window and allows the user to manually tune/match each nucleus separately. This method is more accurate than the automated "atma" command, which automatically tunes/matches the probe. The "atmm" command is recommended for fine adjustments and for checking the tuning and matching*

Adjust tuning and matching of $^1H$ until the signal is located near the triangle at the bottom and middle of the graph.

File > Save position after tuning/matching.

When you've tuned and matched, close the AtmaControl window.

Shim the sample with the TopSpin command `topshim gui`

Set the following parameters for shimming:
Dimension: 1D
Optimization: solvent's default
Optimize in: 1H
Use Z6: Checked
Before: Z-X-Y
After: Z-X-Y-XZ-YZ-Z
Only: Unchecked

Click Start

Following shimming, click the "Report" tab. Check for final $B_0$ deviation of < 1 Hz.

If the number is higher, repeat the 1D shimming process or perform a 3D shim.

You must perform the lock then tune/match then shimming procedures every single time you load a sample into the NMR spectrometer.

If needed, set the temperature for the experiment with the TopSpin command `edte` *298.15K = 25°C ("Room temperature")*

<u>Record the 1D $^1$H NOESY spectra with pre-saturation</u>

Click "Create Dataset"

NAME (Date_Protein_Initials)
EXPNO (1)
PROCNO (1)

In the Experiment panel, click "Select" and browse for the parameter set "noesypr1d"
*Links to example parameter sets are also provided in the Data Availability statement*

Select it then click "Set selected item in editor". Then click "OK"

Open the new experiment by doubling clicking the pulse sequence name under the folder you just created.

In the "Title" tab of the experiment, add information on the sample/experiment.

Example:
*400 uL filtered wine + 100 uL 5X NMR buffer*

*Red: Beaujolais Nouveau: 2024 Georges Duboeuf Beaujolais Nouveau, Burgundy, France*
*298.15K*
*noesypr1d: 1D 1H noesy with presaturation water suppression*

Determine the 90 degree pulse power (p1 value) using the TopSpin command `pulsecal`
*Note: The 90 degree pulse power is a constant value for the spectrometer that doesn't change, but the pulse length is sample/buffer dependent.*

Check the acquisition parameters for the 1D $^1$H NMR as follows:

Use the command `eda` to open the basic parameters window

- Set NS (number of scans/transients) to 128. NS can be increased or decreased depending on your desired signal to noise (S/N) ratio.

- Set DS (dummy scans) to 8.

- Set the RG (receiver gain) automatically with command `rga`
*Note: Receiver gain will be much lower than normal due to the intense ethanol peaks found in wine. Expect a range of 4 to 20 for the RG*

- Set Acquisition Time (AQ) to 4 sec

- Set the Spectral Offset/Carrier Position (O1P value) to 4.7 ppm

- Set the Spectral Width (SW) to 12 ppm

Use the command `ased` to open the complex parameters window

- Set pre-saturation (water suppression) based on your p1 value using `edprosol`
*Note: The parameter for this is pl9 and is usually in the range of 40 to 50 dB*

- Set the relaxation/recycle delay (d1) to 2 sec.

- Check the 90 degree pulse power (p1). Double check that p1 (90 degree pulse value) is correct and what you determined using pulsecal above.

Once you're satisfied the acquisition parameters set, use the command `zg` to start the experiment.

The TopSpin command `expt` will tell you how long the experiment will take, approximately 13 to 15 minutes with the above parameters.

Once the experiment has finished, process the spectra by performing a Fourier transform of the FID using the TopSpin command `efp`

Automatically phase the spectra with the command `apk`

*If manual phasing is required, go to the "Process" tab at the top of the TopSpin window. Click "Adjust Phase". Using the mouse, click and hold the "0" while moving the mouse up and down to phase the spectra. When you finish, click the "Save & Return" button. The icon looks like a floppy disk with a blue arrow.*

## ***Downloading the Data from the Manuscript***

Visit the GitHub repository that contains the raw NMR data, the processed NMR data, the analyzed MagMet-W files, and the associated processing scripts.

[https://github.com/mcshanlab/Capeci_et-al_WineNMR_2025_GitHub](https://github.com/mcshanlab/Capeci_et-al_WineNMR_2025_GitHub)

Click on the < > Code button

Then click "Download ZIP" from the dropdown menu

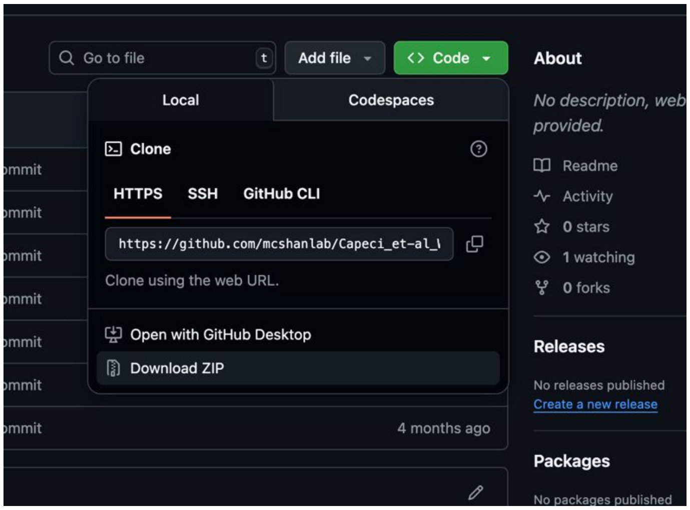


Unzip the downloaded ZIP file

The unzipped folder will contain the following folders:

(1) example_input_metaboanalyst

This folder contains an example .csv file for Multivariate Analysis (see below)

(2) learning-assessment

This folder contains .docx documents of the learning assessments presented in this

manuscript

(3) parameters_wine_nmr_noesypr1d_700MHz_Bruker

This folder contains all the necessary NMR data acquisition parameters to repeat the studies. Please work with an NMR expert or facility manager to implement them if needed.

(4) raw_data_magmet-w

This folder contains examples of MagMet-W processed files and the associated scripts for analysis of the MagMet-W results

(5) raw_data_nmr

This folder contains all the raw and processed NMR data

## ***MagMet-W Analysis***

Separately compress each NMR data folder containing the "fid" and "acqus" files (i.e., "1") into ZIP format

Perform the following steps separately for the data for each wine. Alternatively, MagMet-W allows a limited number of datasets to be processed simultaneously.

Visit the MagMet website
http://magmet.ca/

Create an account and sign in

On the "Home" page, click the "New Submission" button

1. Under the Upload Spectrum option, click the "Choose File" button to upload your ZIP folder (containing the fid and acqus files)

2. For Submission Name, enter the type and name of your wine. For example: "Red Beaujolais Nouveau"

3. Preprocessed (Optional): None

4. Select Biofluid: Wine (700 only)

5. NMR Frequency: 700

6. Chemical Shift (CS) Reference: DSS

7. CS Concentration: 909.09

*Adjust if your DSS standard concentration is different.*

8. Speed: Standard (~7 min)

Click the "Submit" button

The MagMet-W run should take anywhere from 5 to 10 minutes to run

The resulting window will show:

*Top:* JSpectraViewer (JSV) view of the experimental NMR spectra (black – "Spectrum") and the MagMet fitted spectra (blue – "Fit")

*Bottom:* A table with the HMDB ID, Compound Name, Compound Amount (in μM), and Confidence Score.

The HMDB ID is the human metabolome database ID. If you click the link, you can visit a page outlining the structure and function of the compound.

The Confidence Score ranges from 0 (no confidence) to 10 (high confidence).

The table can be sorted by Compound Name or Compound Amount by clicking on header names.

Download the results by click the "Download" button and selecting the "Quantities CSV" option.

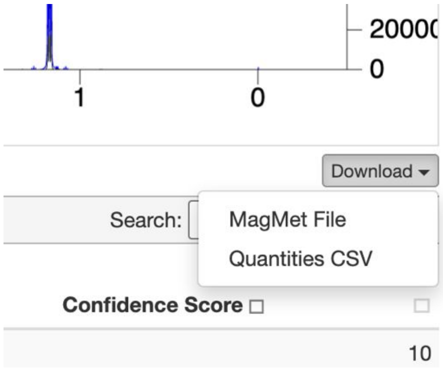


Rename the downloaded file as desired. For example: "Red_BeaujolaisNouveau_magmet_results.csv"

***<u>Data Analysis: Pie Charts of Wine Component Percentages</u>***

Put the .csv file (or several .csv files) downloaded from MagMet-W into a folder.

Each file should have a named formatted like “Red_Merlot_magmet_results.csv”

Make sure the .csv files have unique names.

Execute the script in a Terminal with the command
```
python3 complete_wine_analysis_from_MagMetW.py
```

*Note: the python3 script requires pandas, matplotlib, and seaborn dependencies. These can be installed with pip or conda.*

The script should generate two .pdf files for each MagMet-W dataset that shows the corresponding pie chart summaries. It also generates an excel file with the name compound_comparison_table.xlsx that tabulates all the compound concentrations.

To generate a bar graph to compare specific compounds, edit line 8 of the “bar-graph_one-compound_wine_analysis_from_MagMetW.py” script. Change the name to the desired compound.

Then execute the script in a Terminal with the command
```
python3 bar-graph_one-compound_wine_analysis_from_MagMetW.py
```

The script should generate a .pdf file with a bar graph

## ***Data Analysis: Multivariate Analysis***

The MagMet-W results files (compound vs concentration) should be collated and organized into a separate file called input_metaboanalyst.csv with the following format:

| | A | B | C | D | E | F | G | H | I | J | K |
|---|---|---|---|---|---|---|---|---|---|---|---|
| 1 | Sample | Class | Adenine | Acetate | Betaine | beta-Alanine | Acetoacetat | Carnitine | Citric acid | Choline | Xylose |
| 2 | Red_Beaujol | Red | 7.4 | 3396 | 25.3 | 0 | 2.7 | 81.1 | 0 | 62.6 | 558.[illegible] |
| 3 | Red_Caberne | Red | 6.6 | 6275.1 | 38.2 | 0.1 | 66.6 | 24.2 | 1.7 | 213.7 | 519.[illegible] |
| 4 | Red_Merlot | Red | 6.7 | 4988.9 | 47.9 | 0 | 37.9 | 0 | 0 | 164 | 989.[illegible] |
| 5 | Red_PinotNo | Red | 9.5 | 6996.8 | 27.6 | 0 | 22.8 | 7.7 | 0 | 270.8 | 45[illegible] |
| 6 | Red_Syrah | Red | 7 | 6616.6 | 64.5 | 0 | 29.6 | 13.1 | 6.6 | 299.4 | 630.[illegible] |
| 7 | Rose_Grenac | Rose | 10.6 | 5047.6 | 12.7 | 0 | 8.8 | 58.6 | 25.9 | 172.2 | 82.[illegible] |
| 8 | Rose_Proven | Rose | 28.5 | 3322.9 | 76.8 | 0 | 8.3 | 51.4 | 46.6 | 164.9 | 199.[illegible] |
| 9 | Rose_Sangio | Rose | 9.6 | 3841.9 | 39.3 | 0 | 17.4 | 71.7 | 73.7 | 203.2 | 802.[illegible] |
| 10 | Rose_Tempra | Rose | 0.4 | 5926.9 | 58.9 | 0 | 19.4 | 22.6 | 20.3 | 142.3 | 58[illegible] |
| 11 | White_Charc | White | 29.5 | 3541.6 | 30.3 | 0 | 9.5 | 130 | 3.6 | 130.9 | 166.[illegible] |
| 12 | White_Grune | White | 0 | 2977.9 | 23.8 | 0 | 23.1 | 71.9 | 115.6 | 157.9 | 247.[illegible] |
| 13 | White_Riesli | White | 46.2 | 3765.3 | 75.1 | 0 | 9.9 | 42.2 | 9.3 | 145.1 | 423.[illegible] |
| 14 | White_Sauvig | White | 14.6 | 4745.1 | 48.3 | 0 | 7.3 | 103.3 | 24.7 | 151.4 | 235.[illegible] |
| 15 | | | | | | | | | | | |

*Note: MetaboAnalyst requires at least four items in a class (i.e., at least four red wines, four white wines, and four rose wines) to perform the analysis.*

Visit the MetaboAnalyst 6.0 website at https://www.metaboanalyst.ca/

Click the "Click here to start" button

Click "Continue Analysis"

Choose "Statistical Analysis [one factor]" under the Generic Format option

Data Type: Concentrations

Format: Samples in rows (unpaired)

Data File: + Choose > choose the input_metaboanalyst.csv defined above

Click the "Submit" button to the right

Click the "Proceed" button.

For Sample normalization choose "Normalization by sum"

For Data transformation choose "Log transformation [base 10]"

For Data scaling choose "Auto scaling"

Click the "Normalize" button

Click the "View Results" button

Click the "Proceed" button

Under Chemometrics Analysis option choose "Sparse Partial Least Squares - Discriminant Analysis (sPLS-DA)"

Choose the "2D Scores Plot" tab

Select component for X-axis: 1

Select component for Y-axis: 2

Update font/label size: Increase (++)

Display 95% confidence region: Checked

Display sample names: Checked

Use grey-scale colors: Unchecked

Update the graph visualization
