# Supplementary material for: Automated Chemical Profiling of Wine by Solution NMR Spectroscopy: A Demonstration for Outreach and Education
Source: J Chem Educ. 2026 Jan 6;103(2):833–45. doi: 10.1021/acs.jchemed.5c00652 (PMC12895419; doi:10.1021/acs.jchemed.5c00652)

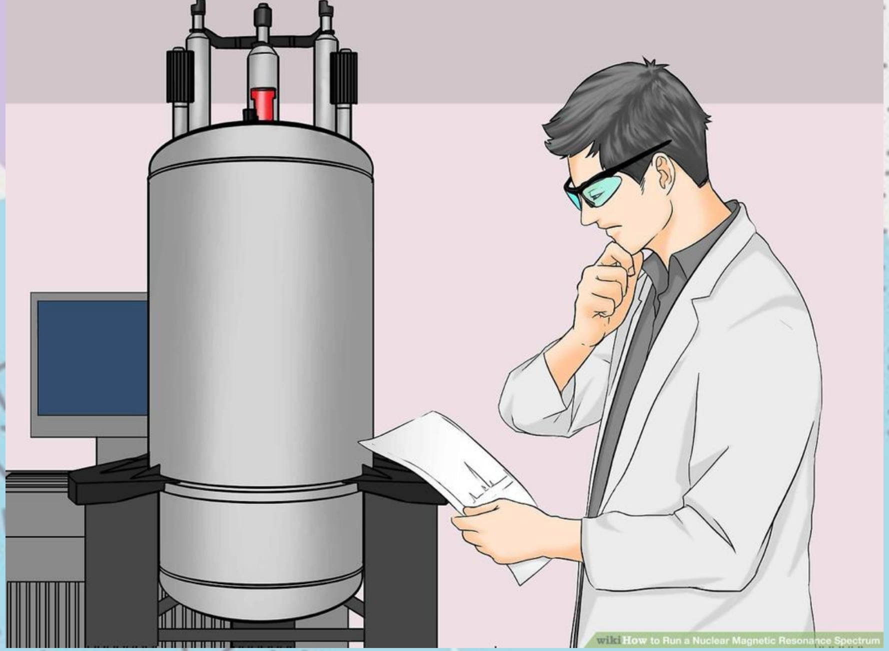

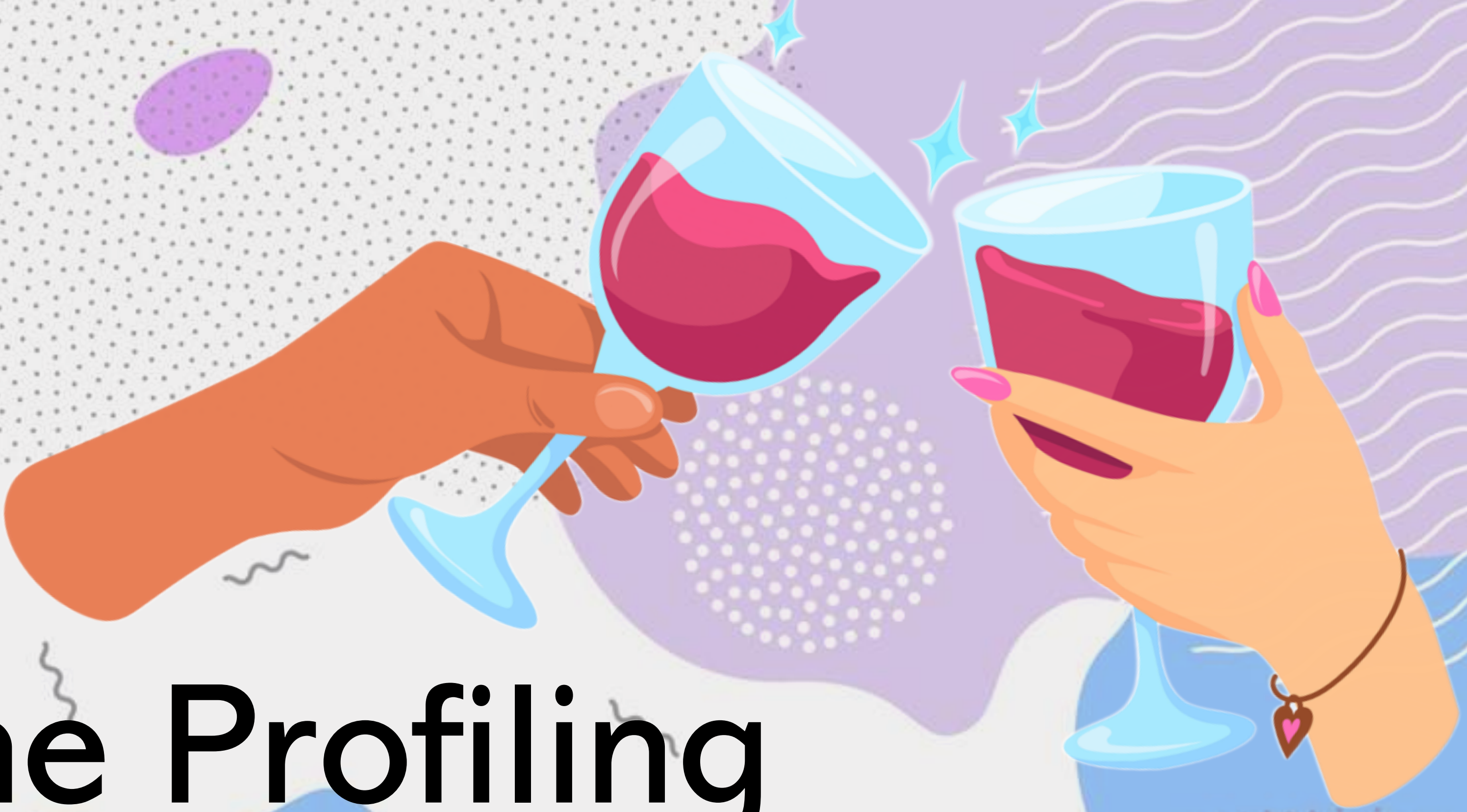

Wine Profiling

NMR Analysis

Anibals

- 93.70% Ethanol
- 6.30% Other

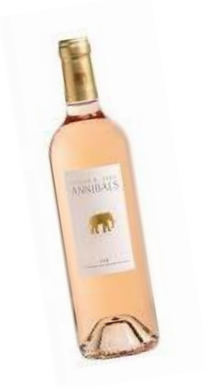

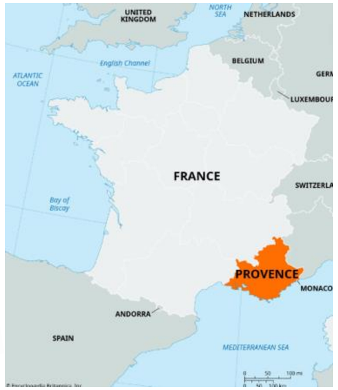

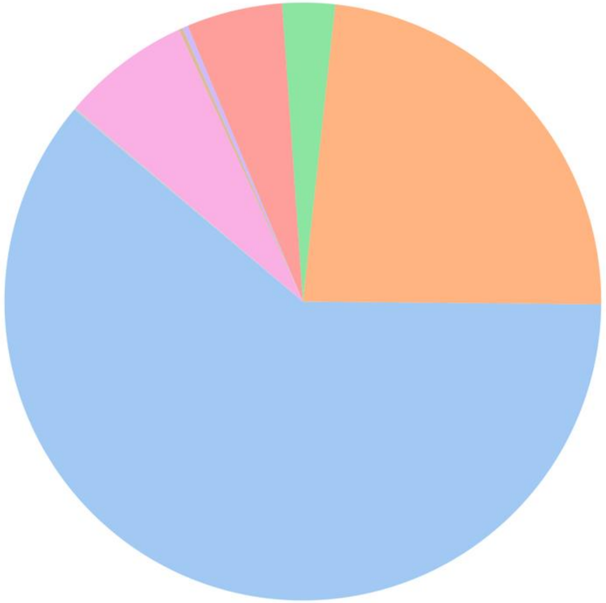

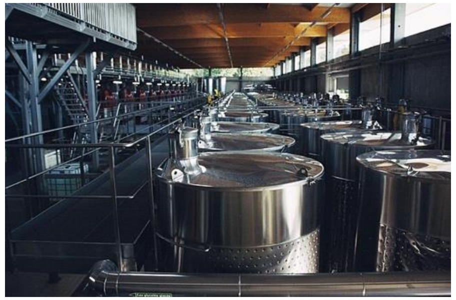

- Cooler fermentation = less unwanted byproducts
- Warmer climate = increase sugar (glucose and fructose) --> alcohol
  - Accumulation during ripening
- 0.5% malate (contributes to a soft and rounder taste)
- Fruity, floral esters

Grenache

- 92.73% Ethanol
- 7.27% Other

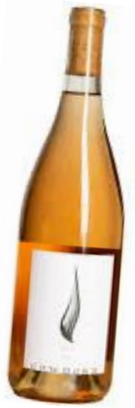

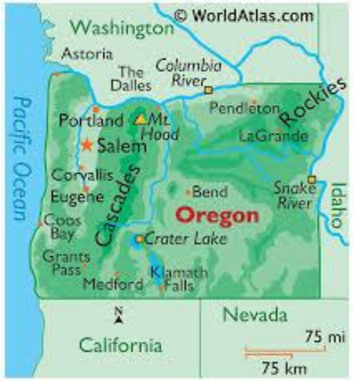

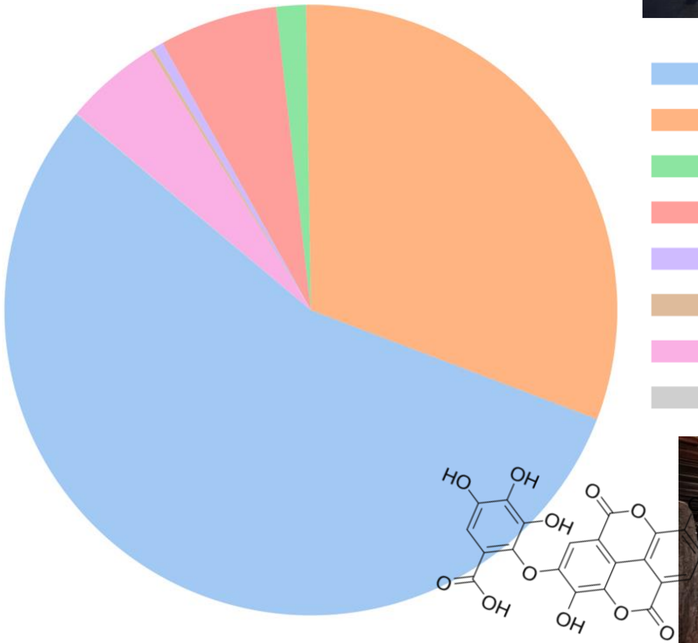

French oak aging! Polyphenols

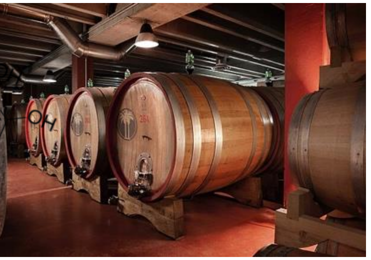

- Warmer fermentation = more unwanted biproduct
- Cooler climate = slower breakdown of acids (drier)
- Less malic acid metabolism before fermentation
- 0% malic acid
  1% lactic acid
  1% tartaric acid (sharper, crisp)

*Malolactic Acid Fermentation by lactic acid bacteria

- Polyphenols = deeper color, spice-like smell

Grenache

92.73% Ethanol
7.27% Other

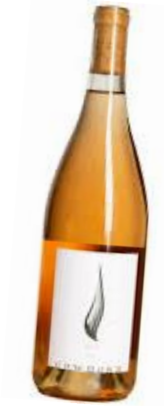

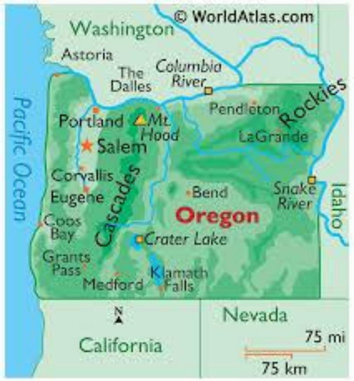

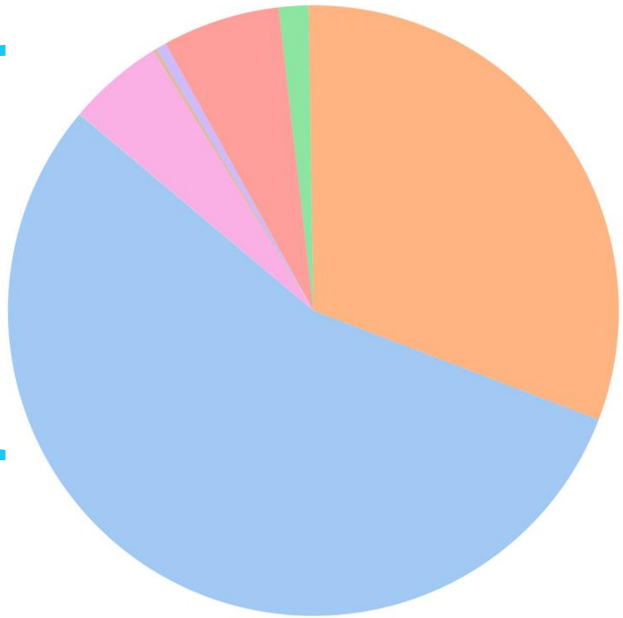

- Brighter, crisp, refreshing, light (higher ethanol, higher acidity)

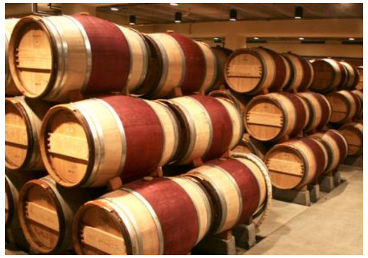

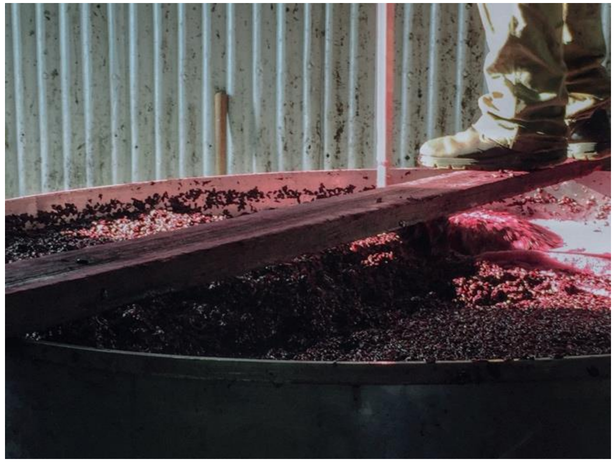

Cabernet

89.91% Ethanol
10.09% Other

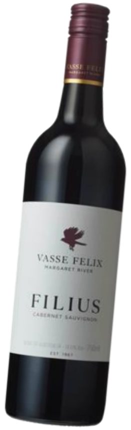

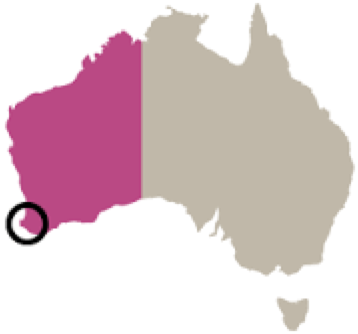

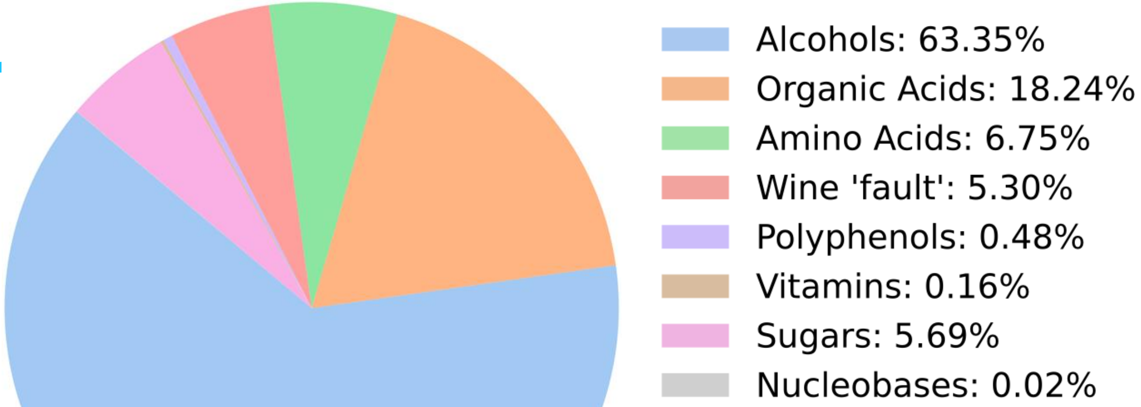

- Complex, rich, structured
- Open barrel fermentation with wild yeast = diversity

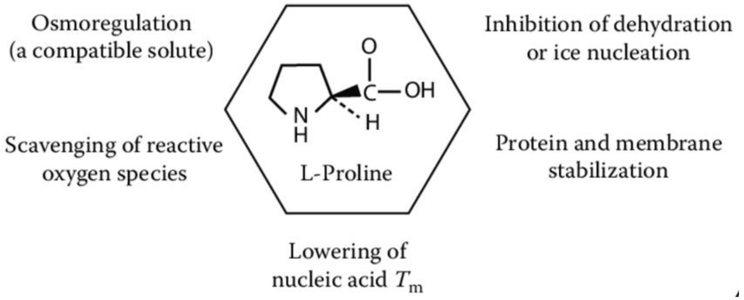

From Takagi, H., Appl. Microbiol. Biotechnol., 81, 211, 2008

"A year of peculiar weather...a crop of naturally resilient grapes"

https://www.vassefelix.com.au/product/2021-Filius-Cabernet-Sauvignon

Cabernet

89.91% Ethanol
10.09% Other

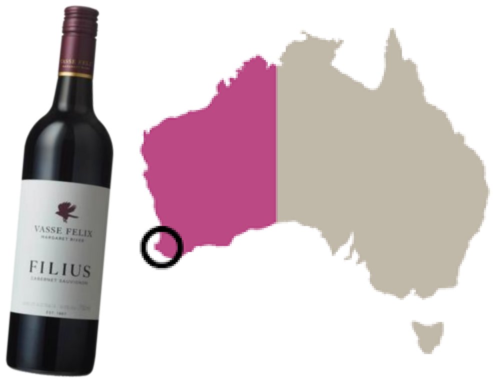

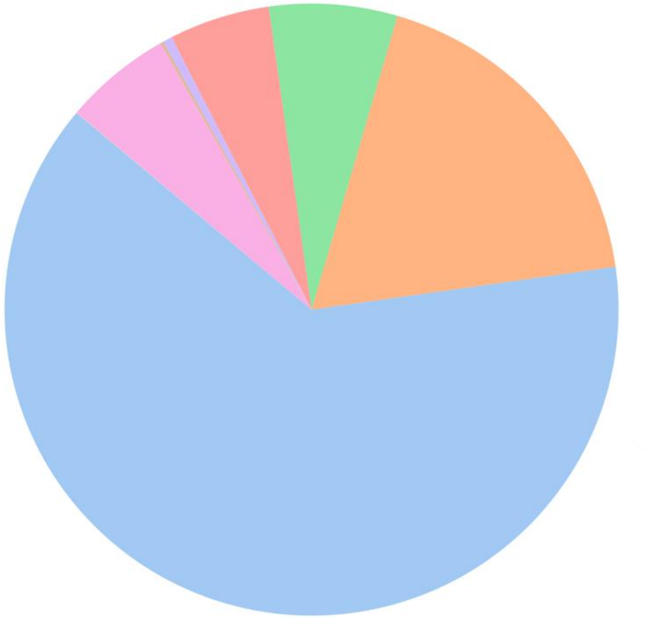

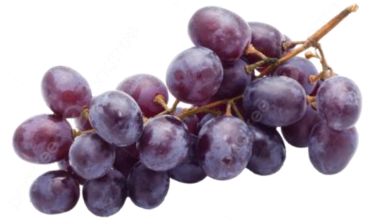

Riesling

85.00% Ethanol
15.00% Other

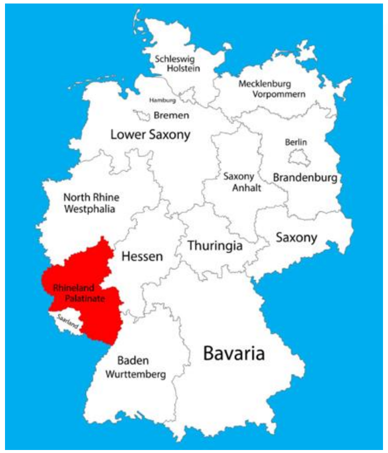

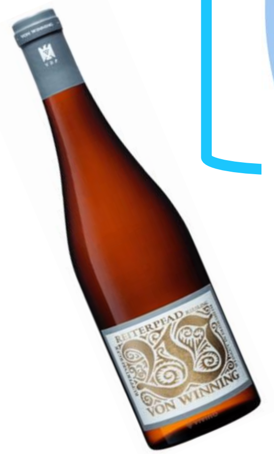

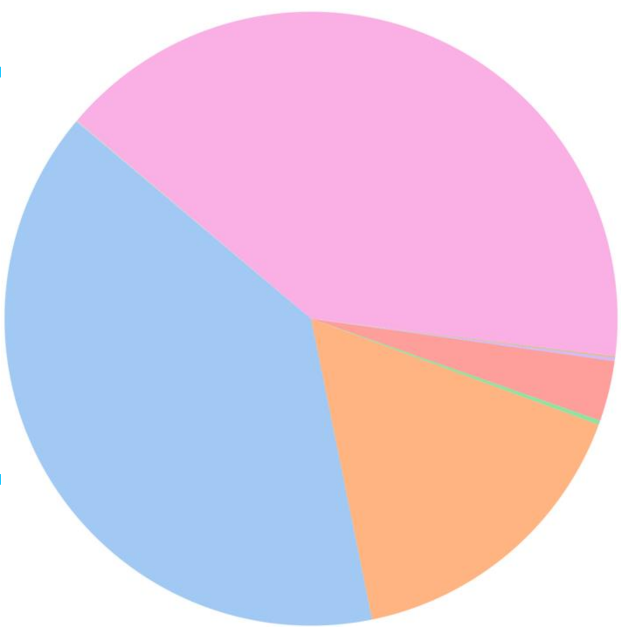

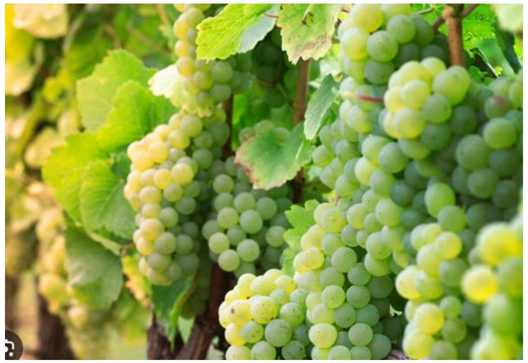

Red

- More polyphenols = darker, structured, rich
- More alcohol, less acid = rounder, fuller
- Less stable over time
- More tannins from grape skin contact during fermentation

White

- More acid = sharper, refreshing
- Residual sugar, lower tannin = sweeter
- Light, crisp, fresh

Red

90.0% Ethanol
10.0% Other

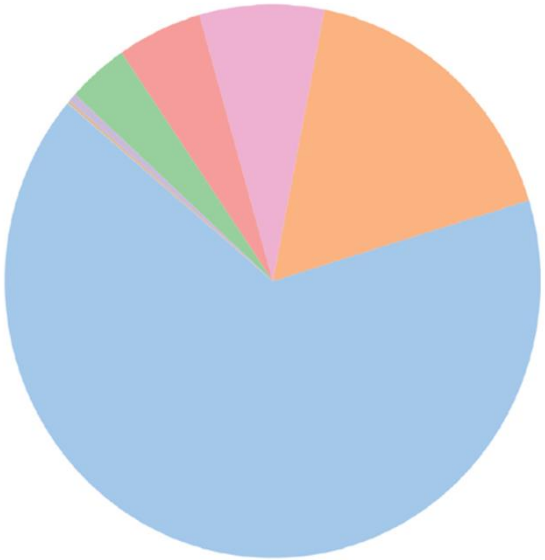

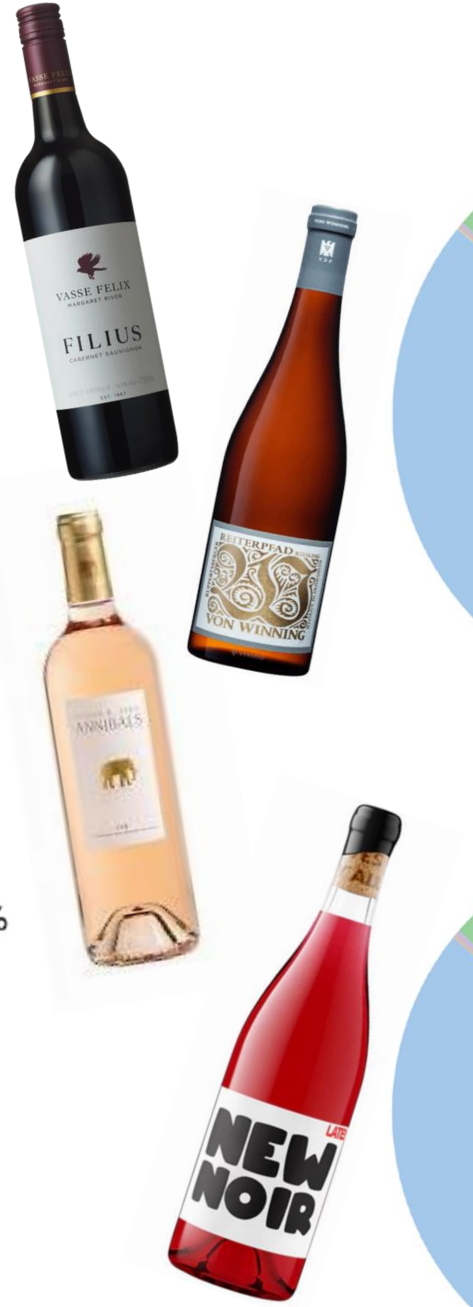

White

89.9% Ethanol
10.1% Other

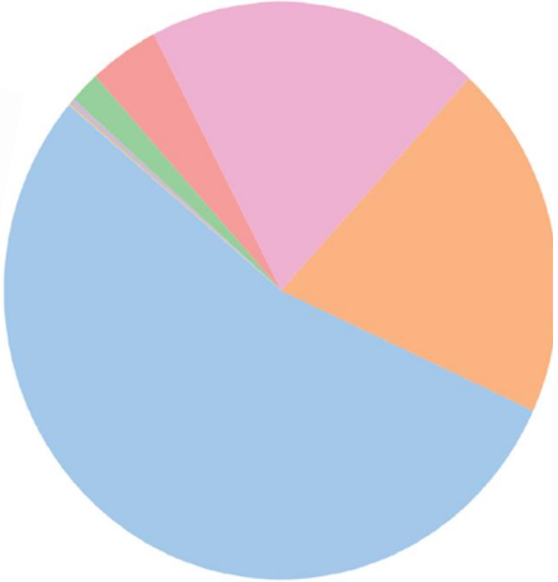

Rosè

92.2% Ethanol = shorter ferm, steel
7.8% Other

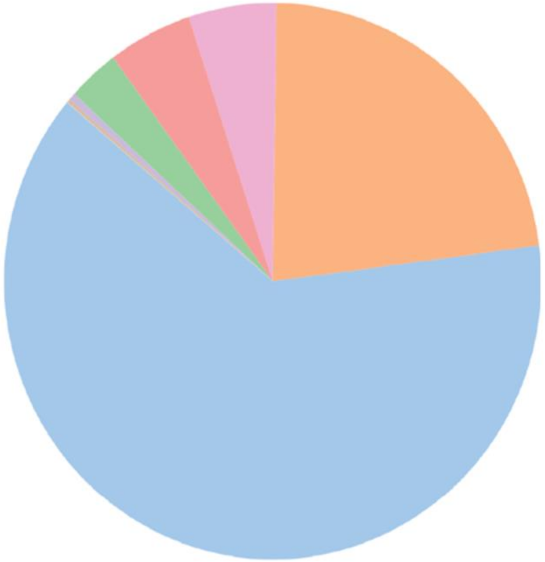

Orange

88.4% Ethanol = wild yeast
11.6% Other

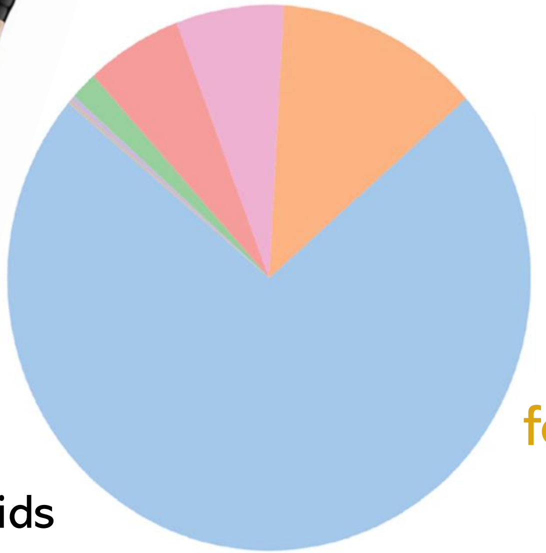

fermented with white grape skins

red < rosè < white
alcohols, acidity, polyphenols, amino acids

Acid of Interest	Lactic	Malic	Tartaric	Citric
Molecular formula	$C_3H_6O_3$	$C_4H_6O_5$	$C_4H_6O_6$	$C_6H_8O_7$
Molecular structure	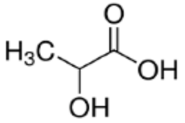	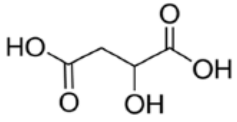	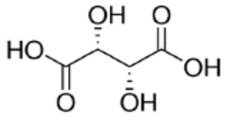	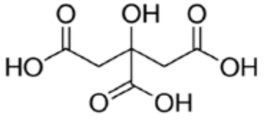
Relative Acidity	$pK_{a1} = 3.86$<br>pK_{a2} = NA	$pK_{a1} = 3.40$<br>$pK_{a2} = 5.20$	$pK_{a1} = 2.89$<br>$pK_{a2} = 4.40$	$pK_{a1} = 3.13$<br>$pK_{a2} = 4.74$<br>$pK_{a3} = 5.40$

Organic Acids

Samarasekara, Dulani et al. “Analysis and Identification of Major Organic Acids in Wine and Fruit Juices by Paper Chromatography.” *Journal of Chemical Education* (2018): n. pag.

Polyphenols

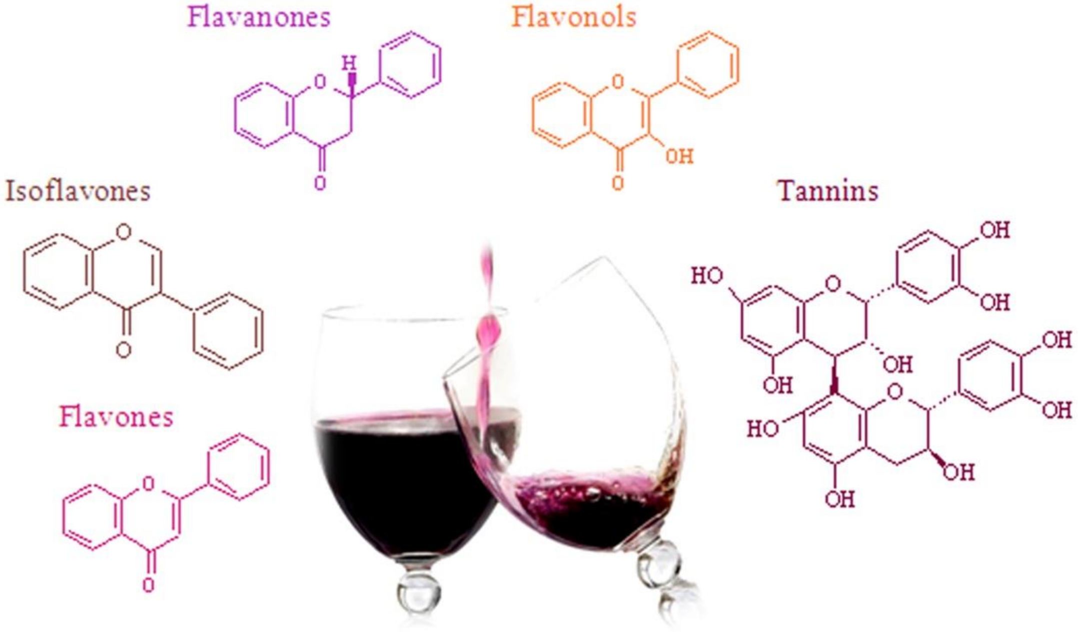

Mitrović, D., Sredović Ignjatović, I., Kozarski, M., & Popović-Đorđević, J. (2024). Wine is more than just a beverage: Chemical diversity, health benefits, and immunomodulating potential of wine polyphenols. *Food Safety and Health*, 2(2), 196–212. https://doi.org/10.1002/fsh3.12036

Sugars

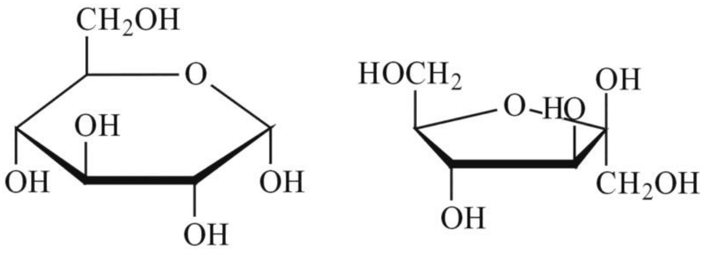

Glucose + Fructose

Faults

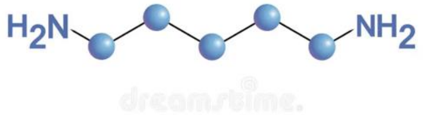

Cadaverine

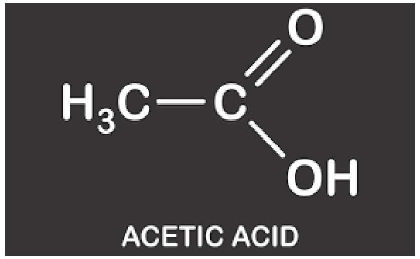

Decarboxylation of amino acids --> cadaverine
Ethanol --> acetic acid acetobacteria

Alcohols

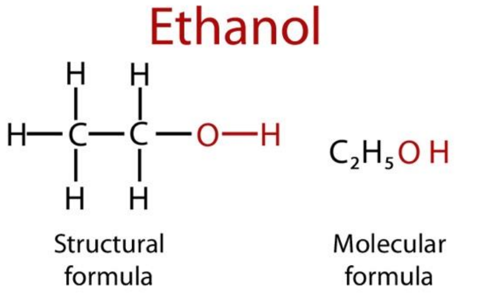

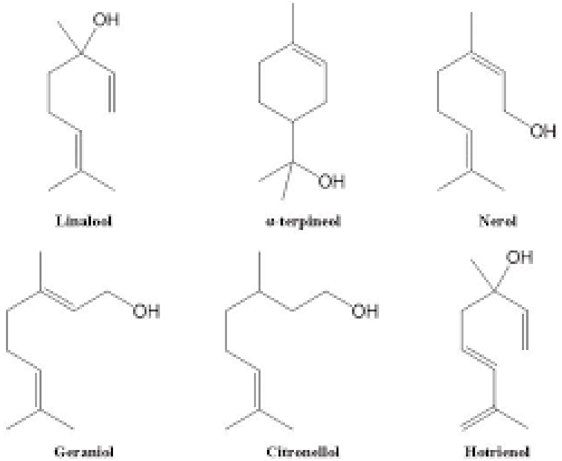

Supplement: Supplementary file 7 [file ed5c00652_si_013.pdf]
